# Cancer origin tracing and timing in two high-risk prostate cancers using multisample whole genome analysis: prospects for personalized medicine

**DOI:** 10.1186/s13073-023-01242-y

**Published:** 2023-10-12

**Authors:** Anssi Nurminen, Serafiina Jaatinen, Sinja Taavitsainen, Gunilla Högnäs, Tom Lesluyes, Naser Ansari-Pour, Teemu Tolonen, Kerstin Haase, Antti Koskenalho, Matti Kankainen, Juho Jasu, Hanna Rauhala, Jenni Kesäniemi, Tiia Nikupaavola, Paula Kujala, Irina Rinta-Kiikka, Jarno Riikonen, Antti Kaipia, Teemu Murtola, Teuvo L. Tammela, Tapio Visakorpi, Matti Nykter, David C. Wedge, Peter Van Loo, G. Steven Bova

**Affiliations:** 1 Faculty of Medicine and Health Technology, Prostate Cancer Research Center, Tampere University and Tays Cancer Center, PO Box 100, 33014 Tampere, Finland; 2 The Francis Crick Institute, London, NW1 1AT UK; 3 MRC Molecular Haematology Unit, Weatherall Institute of Molecular Medicine, University of Oxford, Oxford, UK; 4 Fimlab Laboratories, Department of Pathology, Tampere University Hospital, Tampere, Finland; 5 Charité – Universitätsmedizin Berlin, corporate member of Freie Universität Berlin and Humboldt Universität Zu Berlin, ECRC Experimental and Clinical Research Center, Berlin, Germany; 6 Institute for Molecular Medicine Finland, University of Helsinki, Tukholmankatu 8, Helsinki, 00290 Finland; 7 Imaging Centre, Department of Radiology, Tampere University Hospital, Tampere, Finland; 8 Department of Urology, TAYS Cancer Center, Tampere University Hospital, Tampere, Finland; 9 Manchester Cancer Research Centre, Division of Cancer Sciences, University of Manchester, Manchester, M20 4GJ UK; 10 Department of Genetics, The University of Texas MD Anderson Cancer Center, Houston, TX 77030 USA; 11 Department of Genomic Medicine, The University of Texas MD Anderson Cancer Center, Houston, TX 77030 USA

**Keywords:** Prostate cancer, Cancer evolution, Cancer timing, Cancer anatomic origins, Spatiogenomic evolutionary tracing, Prostate cancer metastasis, Cancer phylogenetics, Cancer genomic drivers, Cancer heterogeneity

## Abstract

**Background:**

Prostate cancer (PrCa) genomic heterogeneity causes resistance to therapies such as androgen deprivation. Such heterogeneity can be deciphered in the context of evolutionary principles, but current clinical trials do not include evolution as an essential feature. Whether or not analysis of genomic data in an evolutionary context in primary prostate cancer can provide unique added value in the research and clinical domains remains an open question.

**Methods:**

We used novel processing techniques to obtain whole genome data together with 3D anatomic and histomorphologic analysis in two men (GP5 and GP12) with high-risk PrCa undergoing radical prostatectomy. A total of 22 whole genome-sequenced sites (16 primary cancer foci and 6 lymph node metastatic) were analyzed using evolutionary reconstruction tools and spatio-evolutionary models. Probability models were used to trace spatial and chronological origins of the primary tumor and metastases, chart their genetic drivers, and distinguish metastatic and non-metastatic subclones.

**Results:**

In patient GP5, *CDK12* inactivation was among the first mutations, leading to a PrCa tandem duplicator phenotype and initiating the cancer around age 50, followed by rapid cancer evolution after age 57, and metastasis around age 59, 5 years prior to prostatectomy. In patient GP12, accelerated cancer progression was detected after age 54, and metastasis occurred around age 56, 3 years prior to prostatectomy. Multiple metastasis-originating events were identified in each patient and tracked anatomically. Metastasis from prostate to lymph nodes occurred strictly ipsilaterally in all 12 detected events. In this pilot, metastatic subclone content analysis appears to substantially enhance the identification of key drivers. Evolutionary analysis’ potential impact on therapy selection appears positive in these pilot cases.

**Conclusions:**

PrCa evolutionary analysis allows tracking of anatomic site of origin, timing of cancer origin and spread, and distinction of metastatic-capable from non-metastatic subclones. This enables better identification of actionable targets for therapy. If extended to larger cohorts, it appears likely that similar analyses could add substantial biological insight and clinically relevant value.

**Supplementary Information:**

The online version contains supplementary material available at 10.1186/s13073-023-01242-y.

## Background

Prostate cancer (PrCa) in 2023 remains a major worldwide stress on patients, their families, and health systems, with 1.4 million newly diagnosed patients and 375,304 patient deaths in 2020 alone [1]. A diverse set of genomic mutation drivers of metastatic prostate cancer (mPrCa) has been reported [2–6]. Mutation heterogeneity in PrCa is likely a root cause of treatment failure [7, 8]. Somatic cancer evolution studies can unravel heterogeneity [9–15], but they have not been applied as a tool to improve outcomes in PrCa.

Unraveling heterogeneity of genomic changes in individual prostate cancers can only occur via evolutionary studies, where the combination of genomic changes present in a set of anatomically localized PrCa cells is compared systematically to genomic changes in a separate set of anatomically localized PrCa cells from the same patient. Collecting the necessary multiple anatomically and time-oriented samples of sufficient quality, obtaining sufficient genomic breadth and depth of coverage, and analyzing the resulting genomic data using appropriate methods are all major challenges to routine evolutionary analysis of primary and metastatic cancers. Recent work relating liquid biopsy-based evolutionary data [13, 16, 17] is exciting and further work is needed to define the relationship between tissue and liquid-biopsy-based evolutionary changes in the context of clinical interventions.

Understanding the evolution of an individual PrCa through serial analysis in space and time may be more important than identifying actionable targets for therapy from single samples as in current practice. The most prominent recent example of this is our recent evolution-based discovery of subclone eradication in a metastatic PrCa [13], with subsequent identification of a novel method to compare eradicated and resistant subclones to better resolve actionable targets for therapy [16]. Evolutionary context was similarly critical to identifying that most mPrCas have monoclonal origins [9, 11], that spread of primary prostate cancer to distant sites often occurs by separate subclones in space and time [11, 12], and that androgen receptor signaling gain occurs multiple times and through multiple mechanisms in the same patient’s mPrCa under the selection pressure of androgen deprivation [7].

Histomorphologic multifocality, where physically separate foci of PrCa are detected in the same prostate, has long been known [18–20]. How often separate histomorphologic foci are also genomically independent clones of PrCA is not yet quantified, but recent studies based on whole genome, exome, FISH, copy number, and immunostaining studies suggest that independent clones of PrCa are likely to exist in many aging prostates [3, 12, 13, 21–31]. Evolutionary analysis of cancer foci in three dimensions in a series of cases will be required for definitive answers to this question.

Better understanding of cancer evolution has been critical to advancing biological understanding and therapeutic targeting in lung, pancreatic, and other cancers [32–35], but it should be emphasized that the total number of cases of PrCa and other cancers studied in detail in an evolutionary context remains small [36, 37], likely due to the aforementioned difficulty of collecting and analyzing the right samples. Just as important, methods for analyzing evolution in cancer cell populations in individual patients are evolving themselves [38] and to date there are no generally accepted benchmarks for testing and proving the various current methods of evolutionary analysis [39]. The early phases of evolutionary analysis of cancer started with cytogenetic observations [40–43] and subsequently advanced through studies of single markers [44], general comparison of genomic content [7, 9], to current methods which rely on multidimensional clustering on whole genome data often using Bayesian methods [13, 45–48], at times including single-cell sequencing [49]. The logical underpinnings of the evolutionary analysis of cancer are strong, since they are based on well-proven genetic principles [50], but much remains to be learned.

Relatively few studies have used multiregional genomic analysis to examine intraprostatic cancer evolution in relation to metastatic capability [10, 12, 13, 51–53], and to our knowledge, this is the first to sample all major cancer foci for analysis based on 3D histology, include multiple metastases in each patient, and use the most recent computational methods for evolutionary analysis. We attempted this in two men with high-risk prostate cancer with the aim of learning how such approaches might be combined with existing clinical methods to potentially improve clinical outcome if applied in larger cohorts.

## Methods

### Patients and tissue dissection

Patients newly diagnosed with PrCa electing radical prostatectomy (RP) with 20% or greater preoperative risk of pelvic lymph node metastasis [54] were eligible for the study under Tampere University Hospital Ethics Committee approval R19074 (Table 1 and Additional file 1: Table S1) and provided written informed consent to participate in the study. Two patients (GP5 and GP12) found to have lymph node (LN) metastases based on post-surgical pathologic analysis were selected for this study. Extended pelvic lymph node dissection was performed in both cases, including lymph nodes dorsal and ventral to obturator vessels, and ventral to internal and external iliac vessels. GP5 was found to have a rare 1 mm positive lymph node in the anterior fibromuscular stroma, and 2/5 left pelvic and 1/7 right pelvic lymph nodes positive. GP12 was found to have 1/9 left pelvic and 1/11 right pelvic lymph nodes positive for mPrCa. Cancer invasion of seminal vesicle (SV) was detected in GP12 but not GP5. Pre- and post-surgical AJCC 7th ed. stages for GP5 and GP12 were T2bNxM0/pT3aN1M0 and T2cNxM0/pT3bN1M0, respectively.

**Table 1:** Patient characteristics

Feature	GP5	GP12
Prostate and urinary history in years prior to PrCa diagnostic biopsy	Age 55.8 symptoms of BPH and PSA 1.8, started on dutasteride. PSA dropped to 0.7 on dutasteride age 58.6, when dutasteride stopped. PSA then steadily rose from 2.8 at age 61, to 12 (percent free 13%) at age 63.6 despite the course of finasteride, just before PrCa diagnostic biopsy	Age 56.2 PSA 2.3 (26% free), Age 58.5 PSA 10 (19% free) led to prostate biopsy
Urinary symptoms just prior to PrCa diagnostic biopsy	Urinary urgency and nocturia	Urinary frequency and decreased urinary stream
Plasma PSA just prior to PrCa diagnostic biopsy	12 ng/mL	10 ng/mL
Family history of cancer in first-degree relatives	History of lethal cancer in father and mother	History of cancer in father
Race/ancestry	White/Finnish	White/Finnish
AJCC 7th edition clinical stage ( in use at study entry)	T2b NxM0	T2c NxM0
Biopsy Gleason Grade Group	5	5
Pathologic stage (after RP)	pT3aN1M0, stage group IVA	pT3bN1M0, stage group IVA
Radical prostatectomy Gleason Grade Group	5	5
Age at PrCa Diagnostic Biopsy/RP	63.6/63.8 years	58.5/58.7 years
Postoperative status (patient follow-up period: up to 6 years after RP)	Received EBRT to prostate fossa in the postoperative period. Put on leuprolide and bicalutamide. PSA nadir post RP 0.3 ng/mL. PSA 6 years postop 0.11 ng/mL. Status M0 at 6 years postop	Received EBRT to prostate fossa and pelvic lymph nodes in the postoperative period. Bone metastasis found 2 years post RP. PSA nadir post-RP 0.9 ng/mL, > 1000 ng/mL at 6 years postop after bicalutamide, orchiectomy, cabazitaxel, carboplatin, and radium 223 treatments, shifted to palliative care with status M1c

The entire tissues (prostate, seminal vesicles, and lymph nodes) removed at RP were sectioned fresh and fixed in PAXgene fixative and processed to preserve anatomic orientation, histology, DNA, and RNA as previously reported [55] (Additional file 1: Supplementary Results). Hematoxylin and eosin (H&E) stained sections were whole-slide imaged (WSI) at 20 × with a Nanozoomer S60 (Hamamatsu). Cancer regions of interest (ROI) and morphologic features in each block-face section WSI were annotated (Cytomine) (Additional file 1: Figs. S1 and S2). Cancer volumes of interest (VOI) for laser microdissection were constructed from 4 μm H&E and interleaved 20 μm sections placed on nuclease and RNAse-free PET membrane FrameSlides (MicroDissect GmbH). From each laser-dissected sample, DNA was isolated using the PAXgene Tissue Allprep DNA method (Qiagen’s PX10 Supplemental protocol). From GP5, 8 primary and 3 pelvic LN metastatic VOI were examined. From GP12, 6 primary, 2 SV invasive, and 3 pelvic LN metastatic VOI were examined. Examined VOI were selected to optimize anatomic dispersion, association with known morphologic risk factors (grade, margin and SV positivity), and research cost.

### DNA analysis and phylogenetic tree construction

Whole genome DNA sequencing was performed to a median read depth of 58X for the normal reference comparison blood leukocyte DNA samples taken just prior to prostatectomy, and 71X for primary tumor and metastatic samples using Illumina Novaseq 6000 machines with S4 reagents (Novogene). Sequenced reads were aligned to human genome reference hg38. Somatic copy number alterations (CNAs) were analyzed using Battenberg-hg38 [46], and structural variants (SVs) were analyzed with SvABA [56]. Subclonal reconstruction of cancer cell populations was done with DPClust [57]. Software and genome reference data sets for the project are listed in Additional file 1: Table S2. Aligned paired-end read variant calling followed GATK best-practices guidelines using GATK 4.1.8.1. Somatic variant calling accuracy was validated in comparison to the PCAWG pipeline (Additional file 1: Figs. S3 and S4, Supplementary Methods). A putative sequencing artifact cluster of undetermined origin was found to be present in both patients and all sequenced samples. The presence of a distinct mutational trinucleotide signature (Additional file 1: Fig. S5) in this cluster with a cosine similarity of 91.3% to SBS48 (“possible sequencing artifact”), along with low and inconsistent CCFs (0.02–0.17) for phylogeny reconstruction ruled out the possibility of cancer cell origin. This cluster was removed from the final results. An integrated list of variants identified in GP5 and GP12 is contained in Additional file 2 (Excel Doc A): Table S3. Structural variants identified in GP5 and GP12 are contained in Additional file 3 (Excel Doc B): Tables S4 and S5. The Battenberg purity and ploidy values of samples are contained in Additional file 3 (Excel Doc B): Table S6. Variants and copy number alterations matching recently described high-risk prostate cancer drivers [2, 4] (Additional file 3 (Excel Doc B): Table S7) were placed on the phylogenetic trees. Details of the bioinformatic analysis of the DNA sequencing data are provided in Additional file 1: Supplementary Methods. All subclonal cancer cell populations found in the sampled extraprostatic tissues (seminal vesicles and pelvic lymph nodes) were considered metastatic for the purposes of this analysis and their matching seeder cell populations inside the primary tumor were classified as having metastatic capability.

### Timing of cancer evolution using [C > T]pG mutations and tandem duplications

In both patients, we considered the branch containing the largest number of CpG > TpG mutations (COSMIC single base substitution signature 1, SBS1) [58] at the age of prostatectomy (63.8 for GP5 and 58.7 for GP12) as a molecular clock to estimate the calendar time of cancer progression in each patient. Similar to tumor evolution timing in the International Cancer Genome Consortium (ICGC) Pan-Cancer Analysis of Whole Genomes (PCAWG) project [6], we simulated an increase in the mutational burden at 10,000 random time points following a uniform distribution up to 15 years before sampling (representing ~ 25% of age at RP for both patients). The increase was modeled as a fivefold acceleration in the mutation burden after random time points, reflecting advanced stages of tumor evolution [6]. A fivefold increase matches the PCAWG estimate for prostate tumors, similar to most cancer types. The acceleration model considered that subclonal private mutations also occurred at fivefold acceleration, and time points that did not allow for the private mutations in each sample to occur at a fivefold accelerated rate were discarded. We derived chronological time estimates (and 95% confidence intervals) by comparing SBS1 exposure in each subclone to the longest SBS1 branch as a time anchor for age at prostatectomy as well as introducing time points of interest where fivefold mutation accelerations occur.

GP5 tandem duplications (TDs) were assigned to evolutionary clusters based on SVs and cancer cell fractions (CCFs) of associated CNAs in the tumor samples using Euclidean distance. After this assignment, we tested for a correlation between CpG > TpG mutations and TDs. The TDs in subclones Ab and A were merged because of their closely matching CCFs in all but one sample (RApex), making the evolutionary assignment uncertain between the pair. Subclones Baa, Bc, Aba, and Aa were omitted from the analysis due to their low CCF (median less than 0.5 in all samples).

### Druggability analysis and mapping to current clinical guidelines

We analyzed the potential druggability of truncal and subclone-specific variants by querying their associated genes in the Drug-Gene Interaction Database (DGIdb) v4.2.0 [59] and related it to current clinical status and drug availability (Additional file 3 (Excel Doc B): Tables S8 and S9.

## Results

### Cancer evolutionary reconstruction in two patients with high-risk prostate cancer

**Fig. 1:**
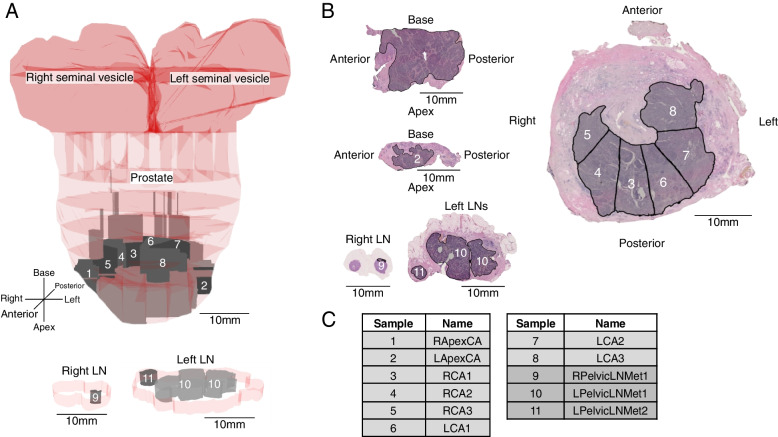
Anatomic overview of GP5 whole genome sequenced samples. **a** Pseudo-3D anatomy of prostate sampling and pelvic lymph node (LN) metastasis regions of interest (ROI). Prostate regions containing cancer are in gray, and sampled volumes of interest (VOI) are the subset in darker gray. GP5 seminal vesicles contained no cancer. **b** Primary and metastatic cancer ROI outlined in black overlayed on H&E stained face sections. **c** GP5 tumor samples analyzed. L/R, left or right; Apex, prostate apex; CA, cancer in primary organ; LN, lymph node; Met, metastasis. See also Additional file 1; Fig. S1. Samples with darker gray background color are from LN metastases

**Fig. 2:**
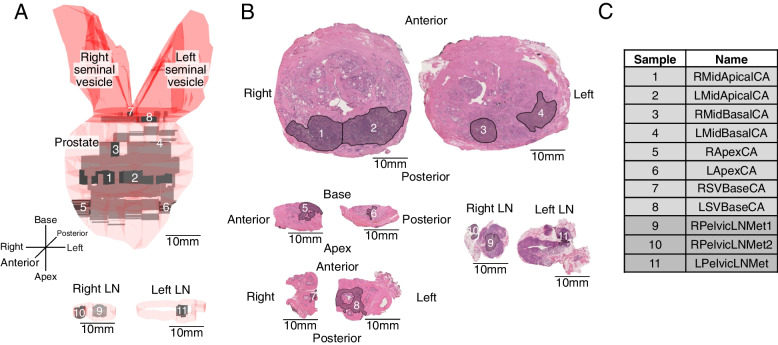
Anatomic overview of GP12 whole genome sequenced samples. **a** Pseudo-3D anatomy of prostate, seminal vesicle (SV), and pelvic lymph node (LN) metastasis regions of interest (ROI) outlined in black and overlaid on H&E stained face sections. Prostate regions containing cancer are in gray, sampled Volumes of Interest (VOI) are the subset in darker gray. **b** Primary and SV invasive cancer and LN metastasis ROI outlined in black overlayed on H&E stained face sections. **c** GP12 tumor samples analyzed. L/R, left or right; Mid, from middle of prostate, away from margins at apex or base; Apex/Apical, prostate apex; CA, cancer in primary organ; SV, seminal vesicle; LN, lymph node; Met, metastasis. See also Additional file 1: Fig. S2

We utilized whole genome sequencing data together with 3D anatomic and histomorphologic information from two men (GP5 and GP12) with high-risk PrCa undergoing radical prostatectomy to reconstruct the evolution of their cancers. A total of 22 sites were profiled from both patients, including multiple samples from the prostate, sites of spread to the SV, and pelvic LN metastases (Figs. 1 and 2, Additional file 1: Figs. S1, and S2). In patient GP5, the large primary tumor mass extended from the prostate posterior mid-apex to the apical surgical margin (Fig. 1a). In contrast, the intraprostatic distribution of GP12 PrCa differed greatly from GP5, with the tumor extending from posterior apex to posterior base and into both seminal vesicles, and only partially filled any given tissue block (Fig. 2a).

**Fig. 3:**
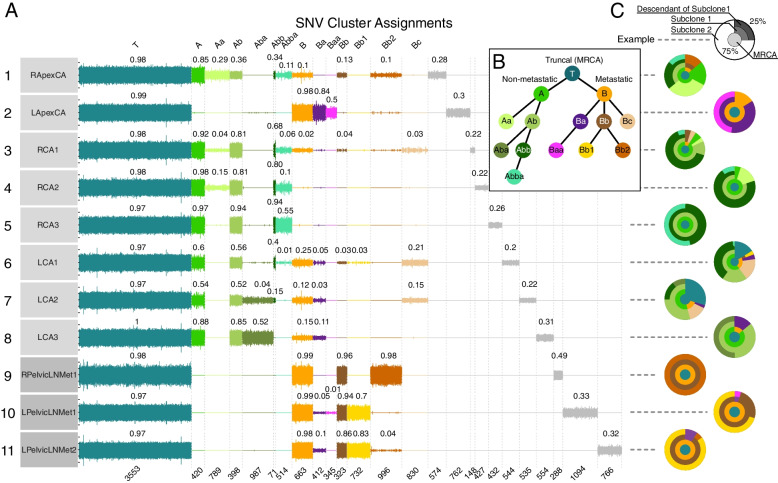
Evolutionary overview of GP5 whole genome sequenced samples. **a** Subclonal reconstruction of single nucleotide variants (SNVs) across the cancer samples (rows) into evolutionary clusters (colored segments in columns). Vertical lines on sample rows represent SNVs with the height of the lines corresponding to their cancer cell fractions (CCF) in the samples, mirrored on the negative side of the axis. Curve-fitted median cluster CCFs are shown on top of each cluster in every sample. The mutations in the most recent common ancestor (MRCA) truncal cluster T are present with a ~ 1.0 CCF in every sample, signifying that these mutations are present in every detected cancer cell. Subclonal SNVs are present with lower CCFs in the samples where the subclone is detected. Mutations detected only in a single sample (private mutations) appear in gray in the rightmost columns. The number of SNVs in each cluster is shown at the bottom of the columns. **b** Cladogram of the evolutionary clusters in GP5 cancer. The MRCA, represented by Cluster T, branched into two distinct lineages, a non-metastatic A-branch (green) and a metastatic B-branch (orange). **c** “Jawbreaker” plots combine evolutionary cluster information with the cluster CCFs to create a summary of the fractions of cancer cell populations present in each sampled location at the time of radical prostatectomy. Example jawbreaker plot at the top in gray describes how descending subclonal lineages are drawn on top of each other so that the surface (outermost layer) of the jawbreaker matches the fraction of cells belonging to the color-matching evolutionary clusters (see also Additional file 1: Fig. S6 for jawbreaker interpretation)

**Fig. 4:**
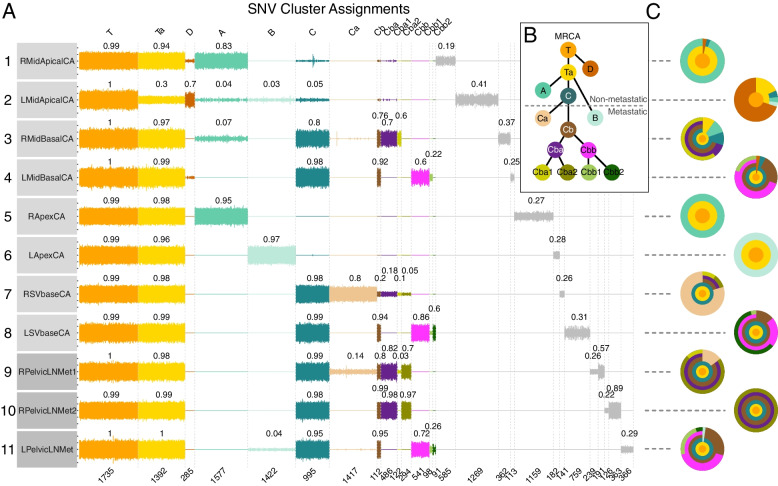
Evolutionary overview of GP12 whole genome sequenced samples. **a** Dirichlet process clustering of GP12 single nucleotide variants (SNVs) across the cancer samples plotted as in Fig. 3a for GP5. Private mutations in samples RPelvicLNMet1 and RPelvicLNMet2 were further divided into two separate clusters. **b** Cladogram of the evolutionary clusters in GP12 cancer. The evolutionary tree has two non-metastatic branches, represented by clusters A and D. The lineage of branch C has undergone the most clonal expansions, putatively representing the most advanced form of the cancer. **c** Jawbreaker plots for GP12 samples

The subclonal composition of the samples from GP5 and GP12 was determined using Dirichlet process clustering of single nucleotide variants (SNVs) (Additional file 1: Supplementary Methods). The clustering revealed between one and seven prominently divergent cancer cell populations in each sampled region of the prostate tumor, as well as the monoclonal origin of the tumors in both patients (Figs. 3 and 4).

In GP5, tumor samples 6-LCA1 and 7-LCA2 contained the largest fraction of cancer cells harboring solely truncal (most recent common ancestor, MRCA) mutations, implicating the left posterior mid-apex region as the putative site of origin of the cancer (Fig. 3a). From this origin, the prostate carcinoma of GP5 diverged into two major branches, A and B (Fig. 3b). Cluster B cells were most prominently present in the left side of the apex (sample 2-LApexCA), while reaching into the rightmost sampled region of the apex as well (1-RApexCA). Cluster A cells occupied the right side of the apical region of the prostate and reached more prominently into the sampled mid-section of the gland (samples 3–8). Strikingly, while cluster A cells represented the larger tumor mass in the sampled regions, only cluster B cells and their descendants were found in the LN metastases (Fig. 3a–c, Additional file 1: Fig. S6). Analysis of the CNA burden associated with metastasis and recurrence in prostate cancer showed significant separation between samples with large metastatic subclone fractions and the remaining samples (Additional file 1: Fig. S7), consistent with B-branch proven metastatic behavior and CNA burden studies [60].

In GP12, the most ancestral form of the cancer was found in the left side of the prostate near the apex (sample 2-LMidApical), indicating this region as the origin of the cancer (Fig. 4a). From its origin, the tumor grew along the posterior prostate, towards the apex, base and both lateral sides of the prostate gland while accumulating unique genetic aberrations in each direction (clusters A–D). Interestingly, as the tumor grew over the midline of the gland, it separated into left and right-sided subpopulations (clusters Cba and Cbb) and this division was maintained in respective right and left-side lymph node metastases (Fig. 4a–c). The metastatic cancer cell populations found on the left side were, therefore, markedly different from the right side samples in terms of their subclonal composition. The dominant cancer cell populations in the metastatic samples consisted of cluster Cba2 (olive green) cells on the right side and cluster Cbb (magenta) cells on the left side. The cancer cells that had metastasized to LNs were found in the basal region of the prostate and the SVs, with the exception of cluster B cells that were found only in the left apical region of the prostate and with a 4% CCF in the left pelvic LN. Overall, cancer cells representing eight different evolutionary clusters were found in the metastatic samples.

### Evolutionary analysis as a lens for interpreting cancer driver heterogeneity

**Fig. 5:**
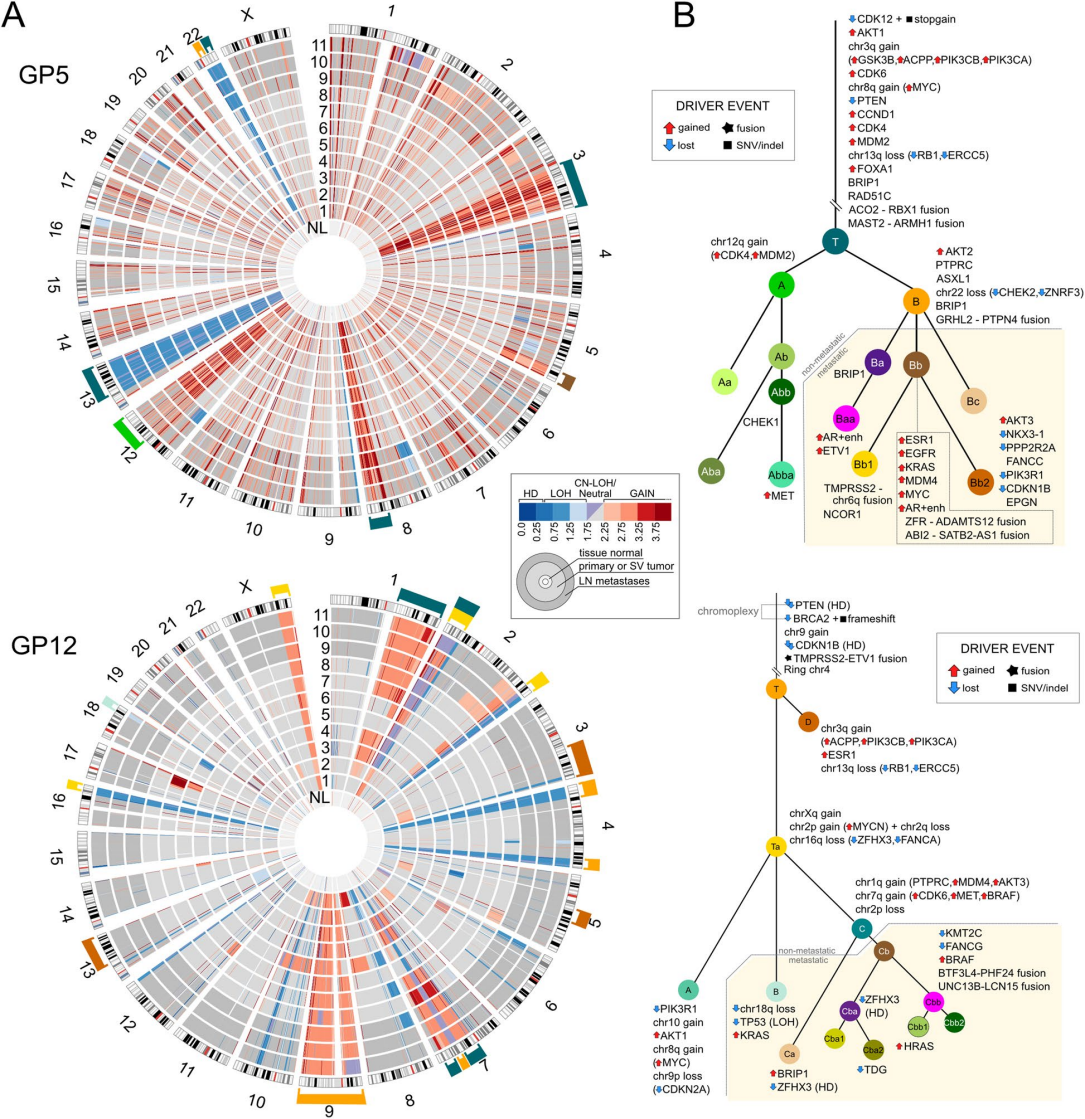
Copy number overview and driver phylogenies in GP5 and GP12. **a** Genome-wide cancer and tissue normal somatic copy number alterations (CNAs), with samples numbered as in Figs. 3a and 4a. Sample “NL” represents a comparison of a noncancerous prostate sample to the blood normal sample. Brackets on the outer rim matching the evolutionary cluster color of panel** b** show the evolutionary assignment of the CNAs. In GP5, the many red “spokes” in the cancer genome represent the 907 < 5 Mb (tandem) duplicated regions likely due to homozygous loss-of-function of *CDK12* in cluster T. Three major chromatid duplications have occurred in chromosomes 3q, 8q, and 12q (red) while large lost regions are detected in chromosomes 13q and 22q (blue). In GP12, major duplications have occurred in chromosomes 1q, 7q, 9, and Xq. Chromosome 4 has lost the ends of both q and p-arms that have subsequently joined together, forming a ring chromosome. **b** Cancer genomic cladograms with internodal distance scaled to the number of SNVs in each segment. Genetic aberrations targeting reported and candidate [2, 4] prostate cancer driver genes are shown next to the evolutionary clusters matching the point in evolution where the event has occurred. Icons adjacent to gene names are used to mark oncogenic driver events, with up (gain of function) and down (loss of function) icons indicating the type of event. Events depicted for each cluster are in no specific order. Genes without adjacent icons are events in prostate cancer driver genes that lack evidence in the literature of being oncogenic. LN, pelvic lymph node; SV, seminal vesicle

Copy number analysis with Battenberg software showed diploid genomes in all samples (Fig. 5a). A total of 35 putatively oncogenic somatic variants were mapped to the GP5 cancer evolutionary tree (Fig. 5b) [2, 4]. One of the most prominent oncogenic driver events was a truncal heterozygous *CDK12* deletion coupled with stop gain (c.C1874G:p.S625X) on the second *CDK12* allele, causing a tandem duplicator phenotype with a distinctive CNA profile with 907 < 5 Mb duplicated regions throughout the genome (Additional file 1: Fig. S8) [61, 62]. While no TMPRSS2-ETS fusions that are typically associated with prostate cancer were found in GP5 PrCa, we discovered a 1.5 Mb chr21 chromatin region containing the TMPRSS2 gene fused into the q-arm of chr6. This aberration was found to be subclonal and only present in cluster Bb1, which is a late-emerging metastatic clone found in the left side LN metastasis. To our knowledge, this is the first reported subclonal TMPRSS2 fusion [63]. The metastatic branch of GP5 acquired significantly more putative drivers (total 17) than the non-metastatic lineage (total 3). We identified two separate instances of gain of *AR* and *AR* enhancer regions in metastatic clusters Bb and Baa (Fig. 5b, Additional file 1: Fig. S8). Cluster Bb cells and their descendants were found with ≤ 12% CCFs in the primary tumor samples but were nearly clonal (CCF ≥ 86%) in all metastases, highlighting their increased metastatic potential. Notably, if the main tumor mass in 4-RCA2/5-RCA3 is declared the “index” lesion, this region contains only 16 of 35 (45.7%) of the putatively oncogenic driver aberrations detected in the metastatic subclones identified in the apices (Supplementary Results). Integrative graphic overviews of copy number, structural variant, and driver changes were produced for all samples (Fig. 5a, b, Additional file 1: Figs. S8, S9).

The most prominent oncogenic somatic driver events in GP12 were homozygous losses of *PTEN* and *CDKN1B*, heterozygous *BRCA2* loss with a frameshift on the second allele (c.3854dupA:p.N1287Kfs*1), and a *TMPRSS2-ETV1* fusion. A total of 35 putatively pathogenic driver events were identified with the large majority being CNAs. A singular chromoplexy event created a rearrangement of chromosomes 7, 10, and 13 (Additional file 1: Fig. S9), resulting in a loss of both a copy of *PTEN* and *BRCA2* in the MRCA cluster (Cluster T) of the evolutionary tree. Similar to GP5, selecting the two largest tumor foci (1-RMidApicalCA/2-LMidApicalCA) as the “index” lesion identifies only 16/36 (44.4%) driver changes identified in GP12 metastatic subclones (Additional file 1: Supplementary Results).

### Tracing the anatomic origins of cancer metastases using evolutionary status

The detailed anatomic annotations available for the samples from GP5 and GP12 allowed for the combination of this information with evolutionary status to reconstruct the paths of tumor propagation and spread in both patients.

**Fig. 6:**
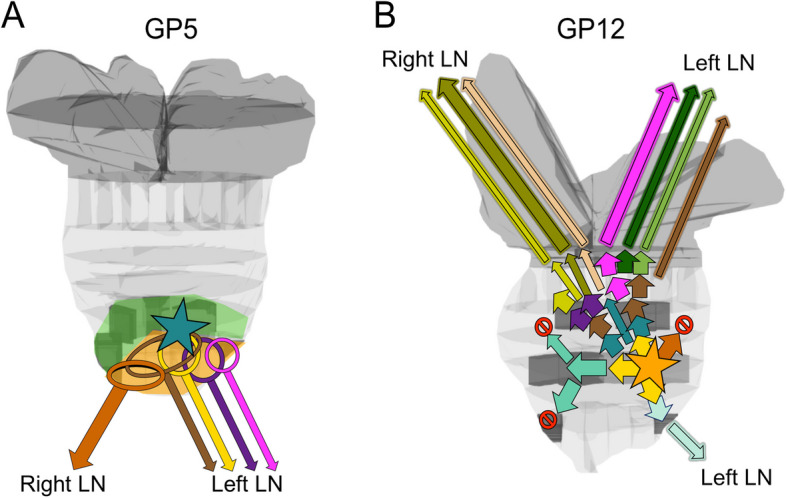
Mapping of the evolutionary clusters onto the anatomy of the prostate in GP5 and GP12. **a** In GP5, the anatomic mapping of non-metastatic and metastatic branches indicates multiple regions involved in metastatic spread. A star icon is shown at the approximate location of the most ancestral clones detected in the tumor, indicating the putative cancer anatomic origin. **b** In GP12, the mapping of the evolutionary clusters onto the anatomy of the prostate shows the path of growth and evolutionary advancement from the posterior left side of the mid-apex (denoted by a star icon) towards the base and seminal vesicles (SV). “No entry” icons indicate evolutionary branches and directions of tumor growth that are not present in any of the sampled metastases (SV or lymph nodes (LN))

In GP5, metastatic spread from prostate to pelvic lymph nodes occurred at least five separate times, by five evolutionarily distinct cancer cell populations represented by clusters Ba and Bb and their descendants (Fig. 3a). The cancer cell populations present in the left apical region of the prostate migrated to the two positive left pelvic lymph nodes, and at least once, spread occurred from the right apex to the right pelvic lymph node (Fig. 6a). The dominant population in the left side LNs (Bb1, yellow) was present in sample LCA1, indicating the left posterior region of the apex as a source of the spread. The dominant right-side LN metastatic cancer cell population consisted of cluster Bb2 cells that were found only in the right apical region of the primary tumor (sample 1-RApexCA). Notably, every descendant evolutionary cluster of the earliest cancer cells with proven metastatic capability also had a presence in the metastases (Fig. 3a, c), suggesting that cancer cell migration from the primary tumor to metastases is an ongoing process rather than a rare event.

In GP12, metastatic spread was traced from multiple locations in the prostate base, apex, and seminal vesicles. The cancer cell populations in sampled region 7-RSVBase closely resembled the cancer cell populations found in the right-side lymph nodes (Fig. 4a, c), indicating it as the putative origin of the right-side metastases. Furthermore, sample 7-RSVBase was also the only known primary tumor location for cluster Cba2, which represented the dominant clone (CCF ~ 90%) in the right-side metastases. The closest resemblance of the cancer cell populations found in the left side lymph node metastasis was present in sample 8-LSVBase, strongly implicating it as the region of the left side metastatic spread. Sample 8-LSVBase was also the only known location of cluster Cbb2 which was found in the left side lymph node as a subclonal population (CCF < 5%). In total, the metastatic spread of GP12 PrCa was traced back to a minimum of eight separate events representing the eight evolutionarily distinct cancer cell populations found in the LN metastases (Fig. 6b). Similar to GP5, all detectable descendant populations of the earliest metastatic populations were also present in the LN metastases and right- and left-sided metastatic subclones metastasized to ipsilateral lymph nodes.

### Chronological origins of primary cancer and cancer metastases

**Fig. 7:**
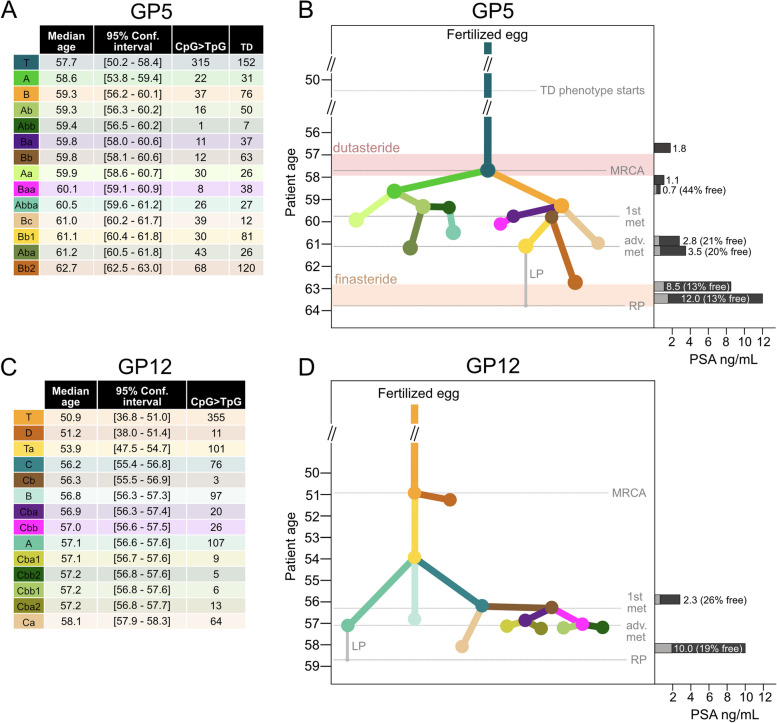
Chronological progress of prostate carcinogenesis. **a**, **c** Analysis of single base substitution (COSMIC SBS) signature 1 mutations (CpG > TpG) shows the evolutionary progression of the cancer in calendar time preceding radical prostatectomy (RP). The median age estimates and 95% confidence intervals (square brackets) of each cluster from simulations starting at random time points up to 15 years before RP are shown. For GP5, the number of tandem duplications (TDs) caused by *CDK12* inactivation detected in each evolutionary cluster is shown in the table as their correlation with SBS1 mutations was used to estimate the start of the TD phenotype. **b**, **d** Age-referenced cancer subclone cladogram for each patient. The nodes in the trees are shown at the age where a single progenitor cell (most recent common ancestor, MRCA) of the evolutionary cluster emerged and subsequently underwent clonal expansion. The gray line connecting a leaf node to RP shows the longest path (LP) of signature 1 mutations that were used in the timing of the emergence of the evolutionary clusters. Timeline markers for the emergence of the first evolutionary clone and the dominant clone detected in the metastasis are noted as “1st met” and “adv. met”. On the right to the cladogram, plasma total prostate specific antigen (PSA) levels are placed on the timeline in dark gray, together with corresponding percent free PSA values in light gray when available. GP5 received dutasteride and finasteride for symptoms of benign prostatic hyperplasia during the time periods marked with pink and orange boxes

We used clock-like CpG > TpG mutations (COSMIC signature SBS1) [58] to track the cancers of both GP5 and GP12 chronologically (Fig. 7) within 95% confidence intervals (CI) (Fig. 7a, c). The first detectable clonal expansion in GP5 occurred around the age of 57.7 (50.2–58.4, 95% CI) (Fig. 7a, b). Thirteen more clonal expansions represented by the evolutionary clusters and signifying advancement of cancer [8] occurred in the following 5 years, estimated between ages 57.7 (50.2–58.4, 95% CI) and 62.7 (62.5–63.0, 95% CI) before RP was performed at age 63.8. The most ancestral populations of cancer cells present in the LN metastases (clusters Ba and Bb) emerged at the age of 59.8 (58.0–60.6 and 58.1–60.6 for Ba and Bb, 95% CI), 4 years prior to RP. The dominant metastatic cancer cell lineages (Bb1, ~ 75% of left side and Bb2, 100% of right side LN, Fig. 3a, c) emerged at the age of 61.1 (60.4–61.8, 95% CI) and 62.7 (62.5–63.0, 95% CI), respectively, 2.7 and 1.1 years prior to RP.

Analysis and evolutionary cluster assignment of the 746 TDs detected in the genome of GP5 that could be assigned to subclones (Fig. 3, Additional file 1: Fig. S10) showed a linear correlation with SBS1 mutations (Additional file 1: Fig. S10) at a rate of ~ 2 accumulated TDs per one SBS1 mutation (*p*-value 0.00223). This observation allowed us to estimate the critical time point in the advancement of GP5 cancer when the TD phenotype became active due to biallelic inactivation of *CDK12* [61, 62], placing it at approximately 50 years of age, after ~ 75% of the MRCA mutations had occurred.

The chronological progression of GP12 PrCa analyzed using SBS1 mutations placed the earliest detectable branching event at age 50.9 (36.8–51.0, 95% CI). Rapid evolutionary progression of the cancer was observable after the age of 53.9 (47.5–54.7, 95% CI), 4.8 years prior to RP at age 58.7 (Fig. 7c, d). Between ages 54 to 58.7, eleven detectable clonal expansions occurred as the carcinoma advanced. The most ancestral cancer cell population (cluster Cb) found in the SV and LN metastases emerged at 56.3 (55.5–56.9, 95% CI) years of age (2.4 years prior to RP), while the dominant metastatic populations of clusters Cba2 (~ 85% of right side LN mets) and Cbb (~ 40% of left side LN met) emerged 1.5 (56.8–57.7, 95% CI) and 1.7 (56.6–57.5, 95% CI) years prior to RP, respectively.

### Evolution in relation to clinical course and histomorphology

In both cases, plasma total PSA and several simultaneously measured percent free PSA values were available from time prior to diagnosis of prostate cancer (Fig. 7b, d). In both GP5 and GP12, no significant difference in Gleason pattern or stromal appearance was identified histologically on visual analysis of H&E stained sections in regions containing metastatic subclones versus those containing no metastatic subclones.

### Evolutionary analysis can be used as a tool for advancing precision medicine

We hypothesized that cancer evolutionary analyses such as those shown here for GP5 and GP12 could be used to inform and improve patient treatment. We analyzed the potential druggability of somatic tumor variants identified in both patients in the context of somatic cancer evolutionary status, focusing on exonic and splice site variants with predicted protein-altering effects, copy number gains, and homozygous losses observed in the DNA (with the exception of TP53, for which we considered loss of heterozygosity to be potentially druggable) (Additional file 3 (Excel Doc B): Tables S8 and S9, Additional file 1: Supplementary Results). For GP5, *CDK12* inactivation and gains of *AKT1*, *GSK3B*, *PIK3CA*, *CCND1*, and *MDM2* were identified as the top truncal druggable targets that would be most likely to obtain a durable response due to their presence in all cancer cells. In GP12, top truncal druggable targets were the inactivation of *BRCA2*, and *PTEN*. Several druggable subclonal somatic cancer variants were further identified in both patients, such as *AR*, *ESR1*, *EGFR*, *KRAS*, and *MET* in GP5, and *HRAS*, *TP53*, *KRAS*, *BRAF*, and *AKT1* in GP12. The subclonal evolutionary status of these variants makes them less likely to obtain a durable response if targeted exclusively, demonstrating the utility of evolutionary analysis in prioritizing druggable targets and designing combination treatments.

### Druggability analysis

Druggable targets with available drugs and clinical trial evidence of potential value to patients exist in both cases, but many are not yet part of standard care or available in specific trials in Finland (Additional file 3 (Excel Doc B): Tables S8 and S9).

## Discussion

We traced the anatomic and chronologic origins of primary tumor and metastases in two men. We determined that PrCa cells escaped from the prostate of GP5 and GP12 at least 5 and 8 times respectively, in each instance traveling ipsilaterally from the prostate to lymph nodes. We also show that intraprostatically, metastatic subclones can migrate across the midline. Multiple instances of cell transit from the prostate to lymph nodes confirm previous findings [11, 12] and resonate with studies showing that selected patients can benefit from removal of the prostate in locally advanced [64] or even metastatic [65] PrCa, because more prostate dwell-time appears to mean more instances of metastasis.

The results allow the “curative time window” between the first PrCa detectability and first metastasis formation to be addressed. Based on the number of accumulated mPrCa-associated driver mutations, the approximate period that aggressive, but solely nonmetastatic PrCa existed in GP5 was between ages 50.5 and 59.8, and in GP12, between ages 50.9 and 56.3. Therefore, the window for curative RP may have closed approximately 4 and 3 years prior to RP surgery for GP5 and GP12, respectively. The combined timing and evolutionary analysis (Fig. 7b, d) show that if genetic sampling of the cancerous cells would be possible at the onset of the symptoms that prompted the first visits to the clinic, before metastatic potential was reached, the tumor cells would already be clearly recognizable as malignant based on the existing oncogenic driver events.

Extraprostatic spread in both cases occurred solely from the apex, base, or seminal vesicles. If this tendency holds in larger cohorts, staging should become more focused on these anatomic sites. Consistent with this, a “wall” of nonmetastatic tumor existed at mid-prostate (Fig. 1), where the tumor originated in GP5, while GP5’s metastatic tumor evolved as it migrated to and spread from the prostate apex.

In GP5 and GP12, two patients who presented with prostate cancer in a high-risk category who elected to undergo a robotic prostatectomy, cancer sites harboring solely nonmetastatic subclones are large but contain only 45.7% and 44.4% of the oncogenic drivers detected in metastatic subclones, suggesting that dangerous lesions are not necessarily larger and cannot be identified based on histology alone, as witnessed in Haffner et al. [10]. Evolutionary studies could help refine how index lesions are defined. This study did not include an analysis of epigenetic and transcriptomic changes at the bulk or single-cell level, and these will likely further increase the value of gradually reengineering PrCa practice to include evolution as a key element.

Taken together, these results suggest that with similarly detailed analysis of a larger cohort of patients, it may be possible to distinguish metastatic-capable from metastatic-incapable subclones in previously unseen primary tumor biopsies based on evolutionary status (truncal/subclonal), number and combination of driver events, and anatomic location.

## Conclusions

Here we show that PrCa evolutionary analysis allows tracking of anatomic site of origin, timing of cancer origin and spread, and distinction of metastatic-capable from non-metastatic subclones. We therefore provide evidence that cancer somatic evolutionary context is fundamentally important to establishing a framework for effective personalized medicine in PrCa. Our analysis suggests that key aspects of current prostate cancer practice can be improved if similar detailed studies are performed in larger cohorts. Well-established oncogenic driver events in PrCa have a roughly 5% mean frequency in tested cohorts. An evolution-aware longitudinal clinical trial including for example 500 men in intermediate or high preoperative risk categories would enable trial-based treatment designed to formally test the value of such an approach. Taken together with our recent evolutionary-analysis-based discovery that solid tumor subclones can be eradicated [13], and that comparison of eradicated and resistant subclones can guide therapy [16], we believe the case for embarking on a deliberate evolution-based transformation of PrCa care is compelling.

### Supplementary Information

**Additional file 1:** Supplementary Material PDF including supplementary material index, supplementary tables 1 and 2, supplementary figures, supplementary methods, and supplementary references.

**Additional file 2:** Supplementary table S3 (Excel Doc A) Annotated GP5 and GP12 GATK4.1.8.1 Variant list.

**Additional file 3:** Supplementary tables S4-S9 (Excel Doc B).

## Data Availability

Whole genome sequence data for the study are available in the European Genome-Phenome Archive (EGA Accession number EGAD50000000005) [66]. Other supporting data is contained in Supplementary Material.

## Abbreviations

AJCC: American Joint Committee on Cancer (staging system)
AR: Androgen receptor
BPH: Benign prostatic hyperplasia
CCF: Cancer cell fraction
CI: Confidence interval
CNA: Copy number alteration
DGIdb: Drug-gene interaction database
EBRT: External beam radiation therapy
FISH: Fluorescence In-Situ Hybridization
GP: Geoprostate
FusionGDB: Fusion gene annotation database
H&E: Hematoxylin and eosin
HRR: Homologous recombination repair (pathway)
LN: Lymph node
mCRPC: Metastatic castrate-resistant prostate cancer
mPrCa: Metastatic prostate cancer (includes both mCRPCs and metastatic hormone-sensitive metastatic prostate cancer)
MRCA: Most recent common ancestor
MRI: Magnetic resonance imaging
PARP: Poly ADP-ribose polymerase (protein family)
PCAWG: Pan-cancer analysis of whole genomes (project)
PET: Positron emission tomography
PIN: Prostatic Intraepithelial neoplasia
postop: Postoperative
PrCa: Prostate cancer
PSA: Prostate-specific antigen
ROI: Region of interest
RP: Radical prostatectomy
SBS: Single base substitution (signature)
SNV: Single nucleotide variant
SV: Structural variant
SV: Seminal vesicle
TD: Tandem duplication
VOI: Volume of interest
WGS: Whole genome sequencing
WSI: Whole-slide imaging
