# Supplementary material for: Cancer origin tracing and timing in two high-risk prostate cancers using multisample whole genome analysis: prospects for personalized medicine
Source: Genome Med. 2023 Oct 12;15:82. doi: 10.1186/s13073-023-01242-y (PMC10571458; doi:10.1186/s13073-023-01242-y)
Supplement: Supplementary file 1 — Additional file 1: Supplementary Material PDF including supplementary material index, supplementary tables 1 and 2, supplementary figures, supplementary methods, and supplementary references. [file 13073_2023_1242_MOESM1_ESM.pdf]

Additional file 1: SUPPLEMENTARY MATERIAL FOR

Cancer origin tracing and timing in two high risk prostate cancers using multisample whole genome analysis: prospects for personalized medicine

Anssi Nurminen[a], Serafiina Jaatinen[a], Sinja Taavitsainen[a], Gunilla Högnäs[a], Tom Lesluyes[b], Naser Ansari-Pour[c], Teemu Tolonen[d], Kerstin Haase[b,e], Antti Koskenalho[a], Matti Kankainen[f], Juho Jasu[a], Hanna Rauhala[a], Jenni Kesäniemi[a], Tia Nikupaavola[a], Paula Kujala[d], Irina Rinta-Kiikka[g], Jarno Riikonen[h], Antti Kaipia[h], Teemu Murtola[a,h], Teuvo L.Tammela[a,h], Tapio Visakorpi[a], Matti Nykter[a], David C. Wedge[i], Peter Van Loo[b,j,k], and G. Steven Bova[a*]

[a]Faculty of Medicine and Health Technology, Prostate Cancer Research Center, Tampere nUniversity and Tays Cancer Center, Tampere, FI-33014 Finland

[b]The Francis Crick Institute, London NW1 1AT, UK

[c]MRC Molecular Haematology Unit, Weatherall Institute of Molecular Medicine, University of Oxford, Oxford, United Kingdom

[d]Fimlab Laboratories, Department of Pathology, Tampere University Hospital, Tampere, Finland

[e]Charité – Universitätsmedizin Berlin, corporate member of Freie Universität Berlin and Humboldt Universität zu Berlin, ECRC Experimental and Clinical Research Center, Berlin, Germany

[f]Institute for Molecular Medicine Finland, University of Helsinki, Tukholmankatu 8, FIN-00290 Helsinki, Finland.

[g]Imaging Centre, Department of Radiology, Tampere University Hospital, Tampere, Finland

[h]Tampere University Hospital, TAYS Cancer Center, Department of Urology, Tampere, Finland

[i]Manchester Cancer Research Centre, Division of Cancer Sciences, University of Manchester, Manchester, M20 4GJ, UK

[j]Department of Genetics, The University of Texas MD Anderson Cancer Center, Houston, TX 77030, USA

[k]Department of Genomic Medicine, The University of Texas MD Anderson Cancer Center, Houston, TX 77030, USA

*Corresponding author. Prostate Cancer Research Center, Tampere University, Faculty of Medicine and Health Technology, PO Box 100, 33014 Tampere, Finland. Tel. +3582945211. steve.bova@tuni.fi.

Supplementary Material Index

Supp Table		Page
S1	Detailed patient characteristics	3-4
S2	Software and reference data sets used, origins and versions	5-6
S3	Annotated GP5 and GP12 GATK4.1.8.1 Variant list	Excel Doc A
S4	GP5 Structural Variants	Excel Doc B
S5	GP12 Structural Variants	Excel Doc B
S6	GP5 and GP12 purity and ploidy values from Battenberg	Excel Doc B
S7	Driver Gene List	Excel Doc B
S8	GP5 Druggability Analysis	Excel Doc B
S9	GP12 Druggability Analysis	Excel Doc B

Supp Fig		Page
S1	GP5 Cancer Histology	7
S2	GP12 Cancer Histology	8
S3	Validation Method for GP2Men WGS DNA somatic variant calling pipeline	9
S4	GP2Men WGS DNA somatic variant calling pipeline validation results	9
S5	Mutational trinucleotide signature of unknown origin, similar to SBS48	10
S6	Cancer Somatic Evolution “Jawbreaker Plot” Visualization Method	11
S7	CNA burden in patients GP5 and GP12	12
S8	GP5 Somatic Tumor DNA Alteration Whole Chromosome Linear Plots	13-36
S9	GP12 Somatic Tumor DNA Alteration Whole Chromosome Linear Plots	37-60
S10	Correlation of Tandem Duplications (TDs) and CpG>TpG (SBS1) nucleotide mutation per GP5 cancer subclone	61

**Supplementary Methods and Results**	62-68
**Supplementary References**	69
**Abbreviations used in Supplementary Information**	70

SUPPLEMENTARY TABLES

Table S1. Detailed patient characteristics

Feature	GP5	GP12
Prostate and urinary history in years prior to PrCa diagnostic biopsy	Age 55.8 symptoms of BPH and PSA 1.8, started on Dutasteride. PSA dropped to 0.7 on Dutasteride age 58.6, when Dutasteride stopped. PSA then steadily rose from 2.8 at age 61, to 12 (percent free 13%) at age 63.6 despite course of Finasteride, just before PrCa diagnostic biopsy.	Age 56.2 PSA 2.3 (26% free), Age 58.5 PSA 10 (19% free) led to prostate biopsy.
Urinary symptoms just prior to PrCa diagnostic biopsy	Urinary urgency and nocturia	Urinary frequency and decreased urinary stream
Plasma PSA just prior to PrCa diagnostic biopsy	12 ng/mL	10 ng/mL
Family History of Cancer in first degree relatives	History of lethal cancer in father and mother.	History of cancer in father.
Race/Ancestry	White/Finnish	White/Finnish
AJCC 7th edition clinical stage	T2b NxM0	T2c NxM0
Biopsy Gleason Grade Group	5	5
Pathologic stage (after RP)	pT3aN1M0, stage group IVA	pT3bN1M0, stage group IVA
Radical prostatectomy Gleason Grade Group	5	5
Age at PrCa Diagnostic Biopsy/RP	63.6/63.8 years	58.5/58.7 years
RP surgical pathology summary	45 x 45 x 38 mm prostate<br>Gleason grade group: 5<br>Gleason score: 9<br>Most common Gleason grade: 4<br>Second most common Gleason grade: 5<br>Total tumor area: 32%<br>Perineural invasion: Yes<br>Lymphatic invasion: Yes	45 x 45 x 40 mm prostate<br>Gleason grade group: 5<br>Gleason score: 8<br>Most common Gleason grade: 4<br>Second most common Gleason grade: 5<br>Total tumor area: 35%<br>Perineural invasion: Yes<br>Lymphatic invasion: Yes

	High grade PIN: Yes<br>Invasion outside the capsule: Yes<br>Length of extraprostatic invasion: 8 mm<br>Invasion of the bladder stroma: No<br>Tumor in surgical margins: Yes<br>Diameter of largest tumor focus: 32 mm<br>Seminal vesicle invasion: No<br>Left iliac lymph nodes PrCa positive/total: 2/5<br>Right iliac lymph nodes PrCa positive/total: 1/7<br>Note: 1 mm lymph node positive in anterior periprostatic fat.	High grade PIN: Yes<br>Invasion outside the capsule: Yes<br>Length of extraprostatic invasion: 14 mm<br>Invasion of the bladder stroma: Yes<br>Tumor in surgical margins: Yes<br>Diameter of largest tumor focus: 40 mm<br>Seminal vesicle invasion: Yes, bilateral<br>Left iliac lymph nodes PrCa positive/total: 1/9<br>Right iliac lymph nodes PrCa positive/total: 1/11<br>Note: Central comedonecrosis is noted in several areas.
Post operative status (patient follow up period: up to 6 years after RP)	Received EBRT to prostate fossa in postoperative period. Put on leuprolide and bicalutamide. PSA nadir post RP 0.3 ng/mL. PSA 6 years post op 0.11ng/mL. Status M0 at 6 years post op.	Received EBRT to prostate fossa and pelvic lymph nodes in postoperative period. Bone metastasis found 2 yrs post RP. PSA nadir post RP 0.9 ng/mL, >1000 ng/mL at 6 years post op after bicalutamide, orchiectomy, cabazitaxel, carboplatin and Radium 223 treatments, shifted to palliative care with status M1c.
AJCC 8th Ed. current or clinical stage at death	pT3aN1M0, Stage Group IVA	pT3bN1M1c, Stage Group IVB
Key results, prospective effect of results on care based on current guidelines	•Truncal drivers: CDK12 biallelic inactivation, gains of AKT1, GKS3B, PIK3CA, CCND1, and MDM2<br>•Metastatic subclone AR gain and associated AR-V7 and other AR splice variant expression<br>•If progression occurs meeting guideline definition, Docetaxel followed by cabazitaxel next step in current guidelines<br>•DNA-damaging therapy alone and immunotherapy alone currently in trials in CDK12-deficient mCRPC, and recent case reports suggest a combination of these modalities may provide therapeutic synergy but clinical trials are needed.<br>•Druggability analysis identified several other targets prioritized by truncal evolutionary status but no trial framework to provide the drugs (Table S8)	•Truncal drivers: BRCA2 and PTEN biallelic inactivation<br>•Docetaxel followed by cabazitaxel next step in current guidelines.<br>•BRCA2 inactivation confirmed by clinical laboratory, carboplatin added on this basis.<br>•Drug targeting PI3K/AKT/mTOR pathway alone has been largely ineffective, pembrolizomab promising in recent Phase I/II trial (see references)<br>•Other potential clonal truncal targets listed in Table S9

Table S2. Software and reference data sets used, origins and versions.

Software or Data and Version	Obtained at
1000G_phase1 (GATK Resource Bundle)	https://console.cloud.google.com/storage/browser/genomics-public-data/resources/broad/hg38/v0
allelecount rev4	https://github.com/photonchang/allelecount
alleleCounter 4.0.2	https://github.com/cancerit/alleleCount
Annovar v. 2019Oct24	https://annovar.openbioinformatics.org/en/latest/user-guide/download/
Battenberg-hg38 2.2.9 dev, commit 9b2d4bfe1d844544c838aaea8e4b19d9f7ad583e	https://github.com/Wedge-lab/battenberg/commit/9b2d4bfe1d844544c838aaea8e4b19d9f7ad583e
Battenberg hg38 reference dataset	https://ora.ox.ac.uk/objects/uuid:08e24957-7e76-438a-bd38-66c48008cf52
beagle 5.0 12Jul19	https://faculty.washington.edu/browning/beagle/b5_0.html
BWA-MEM 0.7.17 (r1188)	https://github.com/lh3/bwa/releases/tag/v0.7.17
Circos 0.69-9	http://circos.ca/software/download/circos/
CNVkit 0.9.8	https://anaconda.org/bioconda/cnvkit
copynumber 1.26.0, commit b3de1b00165b112bdcbc44be61e236a88a634c9a	https://github.com/ShixiangWang/copynumber/commit/b3de1b00165b112bdcbc44be61e236a88a634c9a
COSMIC GRCh38 v92	https://cancer.sanger.ac.uk/cosmic/download
COSMIC Mutational Signatures v3.2, March 2021.	https://cancer.sanger.ac.uk/signatures/sbs/
Cytomine	https://cytomine.com/
dbNSFP version v4.1c (Annovar)	https://annovar.openbioinformatics.org/en/latest/user-guide/download/
DPClust	Available from the corresponding author upon reasonable request
ExAC 65000 exome allele frequency data v. 0.3 (Annovar)	https://annovar.openbioinformatics.org/en/latest/user-guide/download/
FusionGDB, Fusion Gene annotation DataBase	https://ccsm.uth.edu/FusionGDB/
GATK 4.1.8.1 docker	https://hub.docker.com/layers/broadinstitute/gatk/4.1.8.1/images/sha256-8051adab0ff725e7e9c2af5997680346f3c3799b2df3785dd51d4abdd3da747b?context=ex

	plore
GATK/Haplotype caller(1)	https://gatk.broadinstitute.org/hc/en-us/articles/360037225632-HaplotypeCaller
GEM-mappability build 1.315 (beta)	https://sourceforge.net/projects/gemlibrary/files/gem-library/Binary%20pre-release%203/GEM-binaries-Linux-x86_64-core_i3-20130406-045632.tbz2/download
gnomAD AF-only (GATK Best Practices Bundle)	https://console.cloud.google.com/storage/browser/gatk-best-practices/somatic-hg38
gnomAD v3.0 (Annovar)	https://annovar.openbioinformatics.org/en/latest/user-guide/download/
gnomAD v3.1	https://gnomad.broadinstitute.org/downloads
GRCh38 GCA_000001405.15	ftp.ncbi.nlm.nih.gov/genomes/all/GCA/000/001/405/GCA_000001405.15_GRCh38/seqs_for_alignment_pipelines.ucsc_ids/GCA_000001405.15_GRCh38_no_alt_analysis_set.fna.gz
Homo_sapiens_assembly38 (GATK Resource Bundle)	https://console.cloud.google.com/storage/browser/genomics-public-data/resources/broad/hg38/v0
Homo_sapiens_assembly38 known indels (GATK Resource Bundle)	https://console.cloud.google.com/storage/browser/genomics-public-data/resources/broad/hg38/v0
Mills_and_1000G_gold_standard (GATK Resource Bundle)	https://console.cloud.google.com/storage/browser/genomics-public-data/resources/broad/hg38/v0
picard 2.21.8	https://github.com/broadinstitute/picard/releases/tag/2.21.8
R 3.6.3	https://cloud.r-project.org/bin/linux/ubuntu xenial-cran35/ (apt-repository)
RStudio 1.3.959	https://www.rstudio.com/products/rstudio/download/#download
RefSeqGene 20190929 (Annovar)	https://annovar.openbioinformatics.org/en/latest/user-guide/download/
samblaster 0.1.24	https://github.com/GregoryFaust/samblaster/releases/tag/v.0.1.24
samtools 1.8	https://github.com/samtools/samtools/releases/tag/1.8
SvABA 1.1.3(2)	https://github.com/walaj/svaba/tree/4d7b571356661c4ad34893ca2a47399212e0632b

SUPPLEMENTARY FIGURES

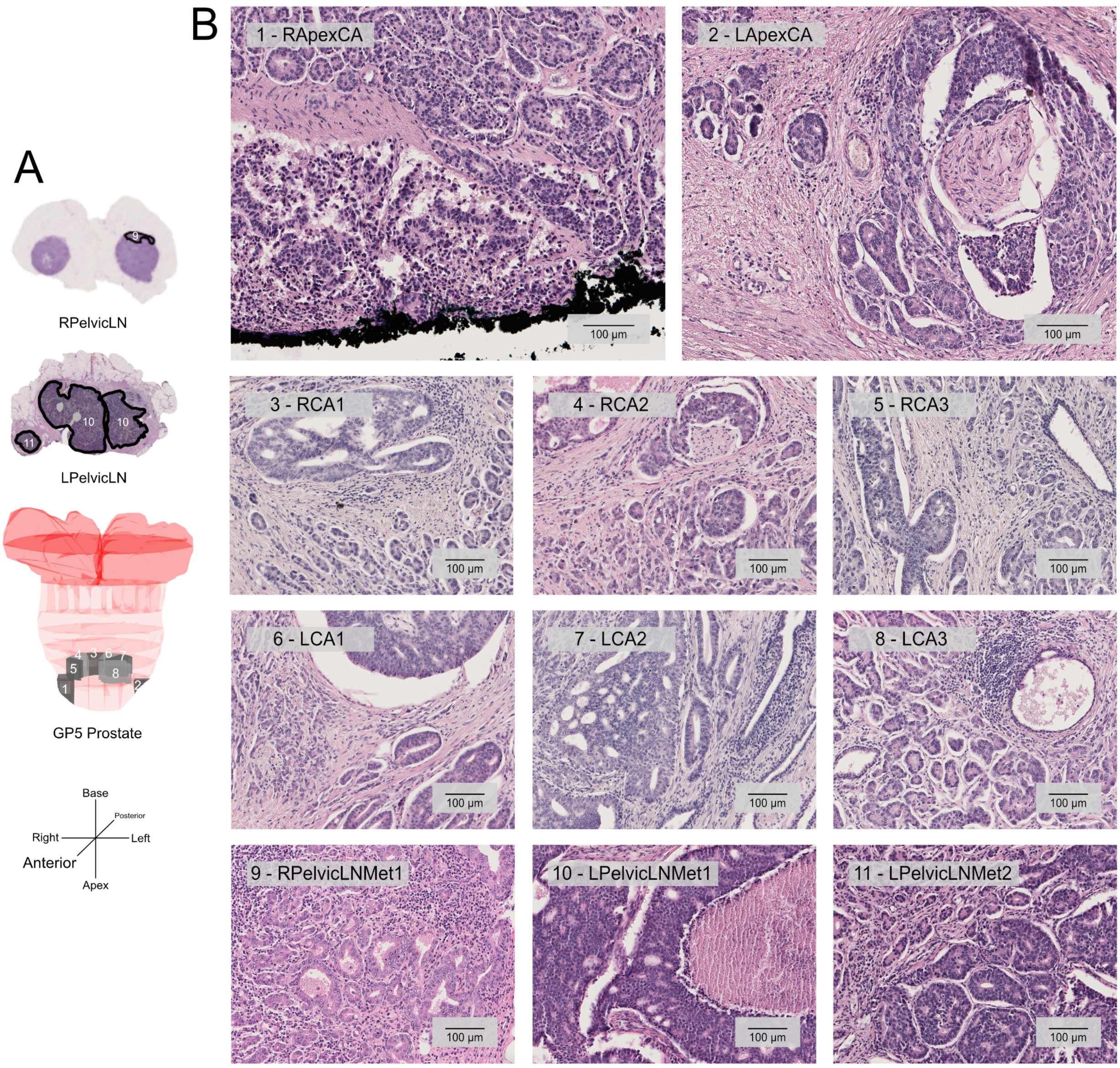

Fig. S1 GP5 Cancer Histology. Representative histology of sampled regions of cancer, focusing on highest risk features in each region of interest. **a** Maps of regions dissected. **b** One image from each of the 11 regions analyzed by WGS, each figure identified with the number and sample name. Notable findings include: Inked surgical margin positivity in 1-RApexCA, perineural invasion in 2-LApexCA, cribriform morphology in nearly all locations, and comedonecrosis in 10-LPelvicLNMet1.

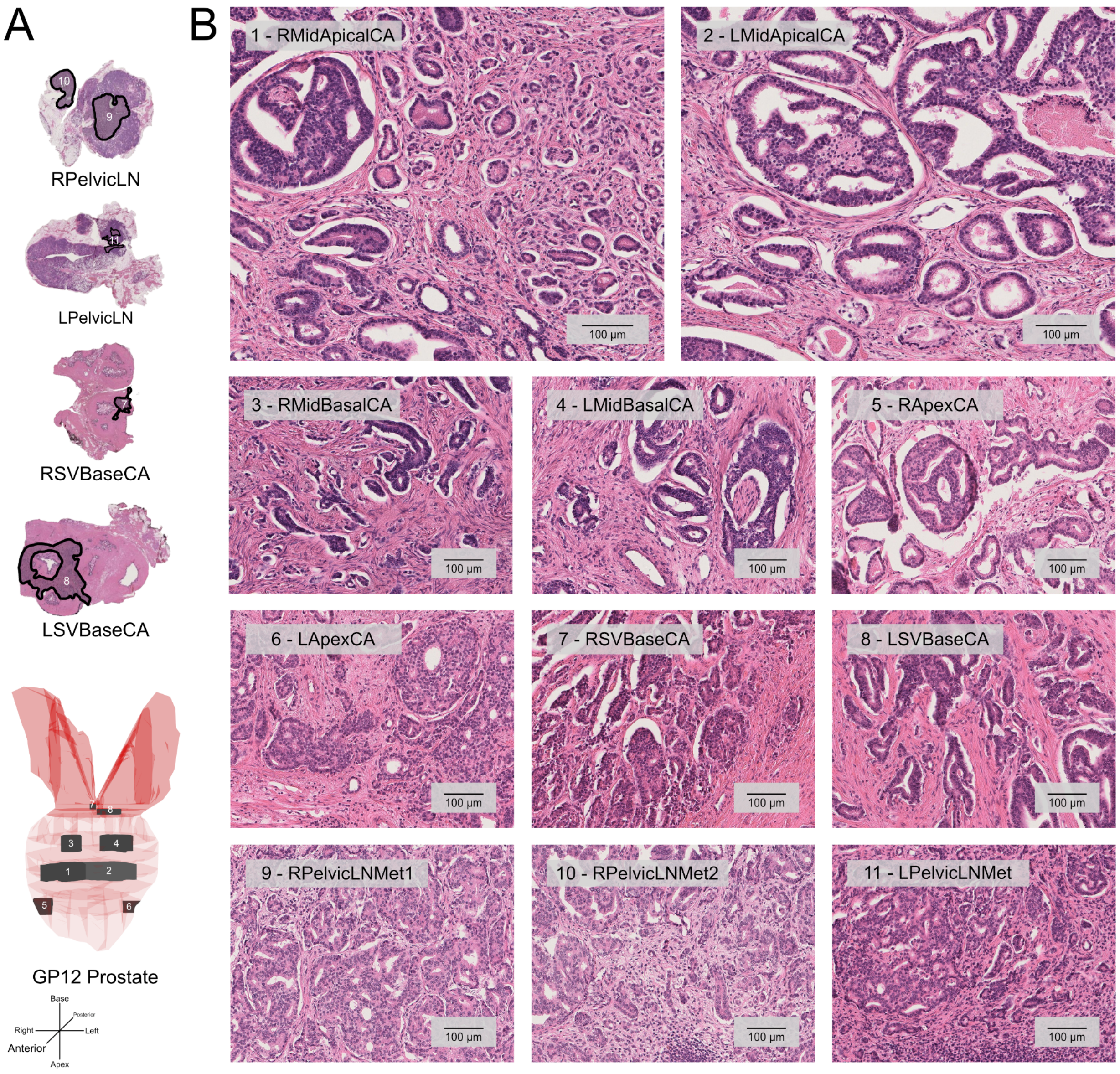

Fig. S2 GP12 Cancer Histology. Representative histology of sampled regions of cancer, focusing on highest risk features in each region of interest. **a** Maps of regions dissected. **b** One image from each of the 11 regions analyzed by WGS, each figure identified with the number and sample name. Notable findings include: Cribriform morphology in many areas, wide variation in morphology in each region including metastases, from gland-forming to single-cell, and wide variation in the amount of intervening stroma.

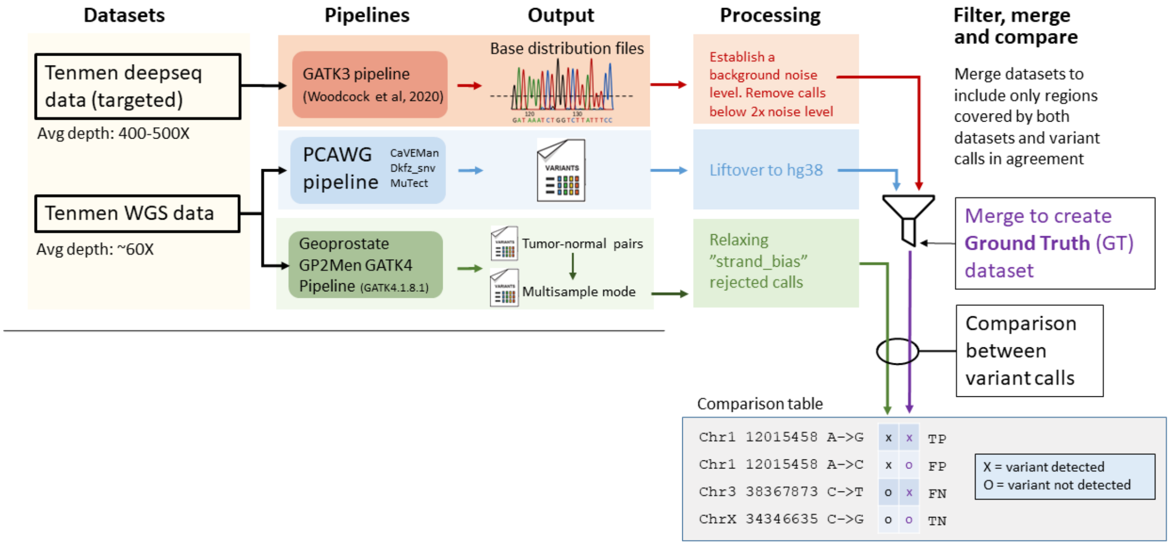

Fig. S3 Validation Method for GP2Men WGS DNA somatic variant calling pipeline. A ground truth dataset was formed from combined variants from "TenMen study" targeted deep sequencing data (3) and WGS data (4) congruent with PCAWG study (5) calls using the same WGS data. The TenMen study WGS data was processed by the GP2Men GATK4.1.8.1 (1) pipeline and resulting variants were compared to the ground truth dataset.

A10-19916-RIliacLNMet

GATK 4181	GT
1	4
308	308
Precision	Recall
0.997	0.987

A12-19947-MediastinalLNMet

GATK 4181	GT
2	4
192	192
Precision	Recall
0.990	0.980

A17-20205-AbdParaaorticLNMet

GATK 4181	GT
6	7
445	445
Precision	Recall
0.987	0.985

A21-19888-LRib5MassMet

GATK 4181	GT
0	18
434	434
Precision	Recall
1.000	0.960

A22-19924-LAdrenalMet

GATK 4181	GT
5	29
808	808
Precision	Recall
0.994	0.965

A24-19954-XiphoidMet

GATK 4181	GT
1	15
423	423
Precision	Recall
0.998	0.966

A29-19933-ProstateCA

GATK 4181	GT
6	14
233	233
Precision	Recall
0.975	0.943

A31-19935-RSubduralMet

GATK 4181	GT
0	10
445	445
Precision	Recall
1.000	0.978

A32-19945-RHumerusMet1-12

GATK 4181	GT
2	26
677	677
Precision	Recall
0.997	0.963

A34-19958-LiverMet12

GATK 4181	GT
9	17
637	637
Precision	Recall
0.986	0.974

Average

Precision	Recall
0.992	0.970

Fig. S4 GP2Men WGS DNA somatic variant calling pipeline validation results. TenMen study (3) WGS and deep targeted sequencing used for validation are identified above each yellow box. In the GP2Men GATK 4.1.8.1/ground truth (GT) comparison in each box, the first row displays the number of disagreeing calls. The left column displays the number of calls detected only by the GP2Men pipeline, and the right column displays the number of calls detected only in GT data. The second row shows the number of agreeing calls. Precision and recall are shown for each sample at the bottom row. The light blue box at the bottom displays the average precision and recall, and for the ten samples they are 0.992 and 0.970 respectively.

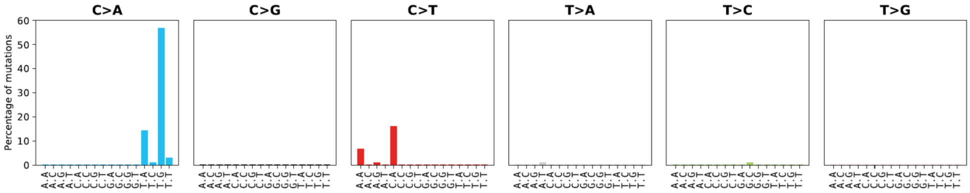

Fig. S5 Mutational trinucleotide signature of unknown origin. A trinucleotide signature was detected in all sequenced samples from both patients with ≤0.05 average CCF. The C>A components match SBS48 (“possible sequencing artifact”) with 91.3% cosine similarity, while the C>T components of the signature are novel. Further testing is needed to determine the origin of this signature, which is likely non-biological. Association of the signature with PAXgene fixation cannot be ruled out.

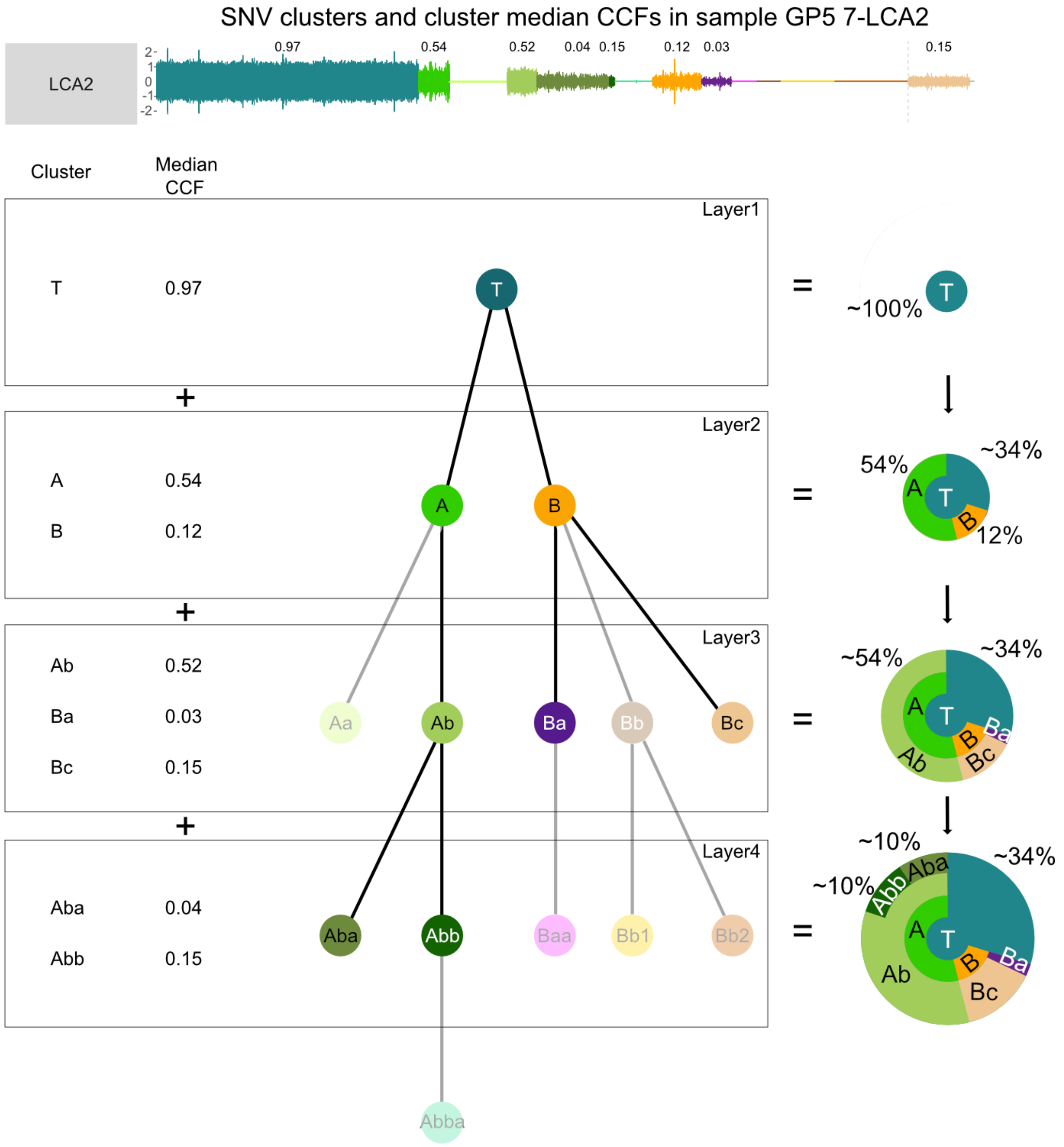

Fig. S6 Cancer Somatic Evolution “Jawbreaker Plot” Construction Method. Using sample GP5 7-LCA2 (shown in Fig. 3a) as an example, a jawbreaker plot is constructed by combining the cluster median cancer cell fraction (CCF) with the evolutionary order of emergence. Each evolutionary cluster represents a group of somatic single nucleotide substitutions (SNVs) that are grouped together based on the similarity of their CCFs in the full set of samples. Starting from the innermost layer (top row), which represents the most recent common ancestor (MRCA) cluster of the descendant subclones, the jawbreaker is built one layer at a time so that layers representing descendant subclones overlap their ancestral clusters. The cluster median CCFs match within 95% confidence intervals between ancestor-daughter clusters, so that the daughter cluster cannot have a higher CCF. The outermost layer of the jawbreaker (bottom row) shows subclones that were populating the sample at the time of RP. Clusters in the cladogram depicted in lighter colors are not present in the GP5 7-LCA2 sample, but exist in other GP5 samples. The fully formed jawbreaker for sample GP5 7-LCA2 illustrates that a large proportion of the cancer cells present in the sample contain only the mutations of the truncal cluster.

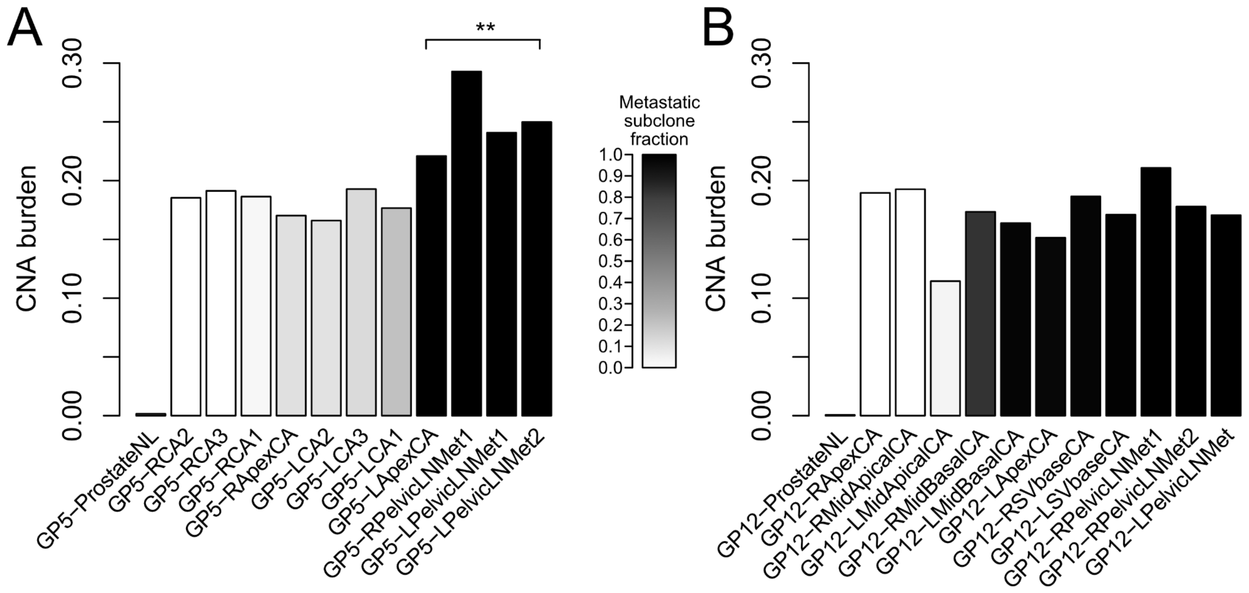

Fig. S7 CNA burden in patients GP5 and GP12. **a** CNA burden in patient GP5 was significantly higher in samples with metastatic subclone fraction above 0.8 (p-value 0.004, Wilcoxon rank sum). A similar association is not observed in patient GP12 samples shown in **b**, but the GP12 sample LMidApicalCA, which consists mostly of cells with non-metastatic cluster D mutations (Fig. 4a) had the lowest CNA burden. Metastatic subclone fraction was defined as the highest potentially metastatic subclone cluster median fraction in each sample.

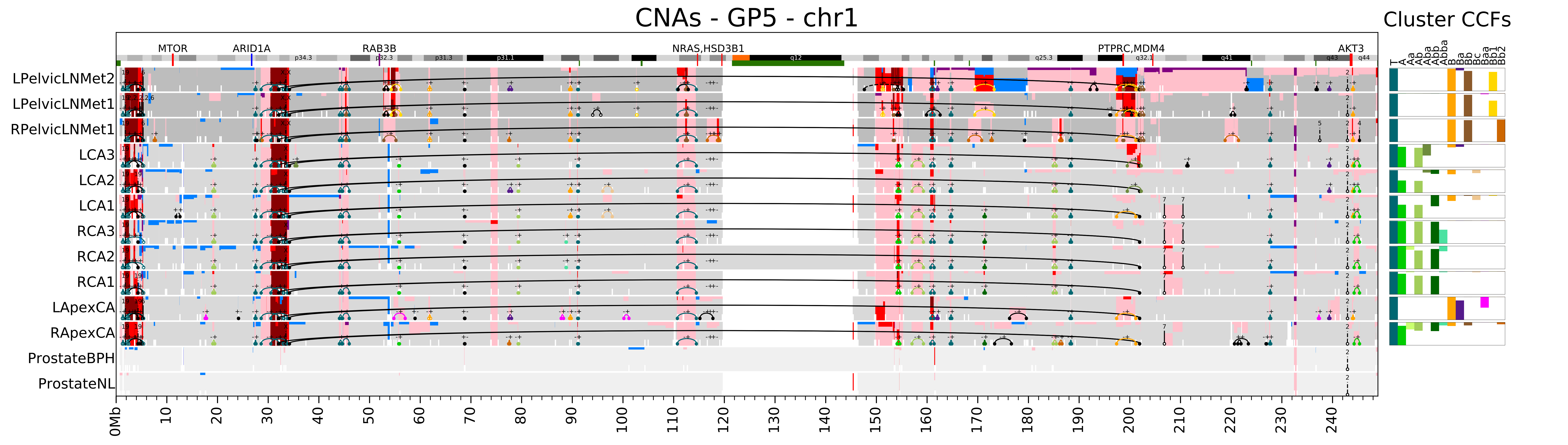

CNAs - GP5 - chr1
Cluster CCFs
MTOR
ARID1A
RAB3B
NRAS,HSD3B1
PTPRC,MDM4
AKT3
LPelvicLNMet2
LPelvicLNMet1
RPelvicLNMet1
LCA3
LCA2
LCA1
RCA3
RCA2
RCA1
LApexCA
RApexCA
ProstateBPH
ProstateNL
0Mb
10
20
30
40
50
60
70
80
90
100
110
120
130
140
150
160
170
180
190
200
210
220
230
240
T
A
Aa
Ab
Aba
Abb
Abba
B
Ba
Bb
Bc
Baa
Bb1
Bb2

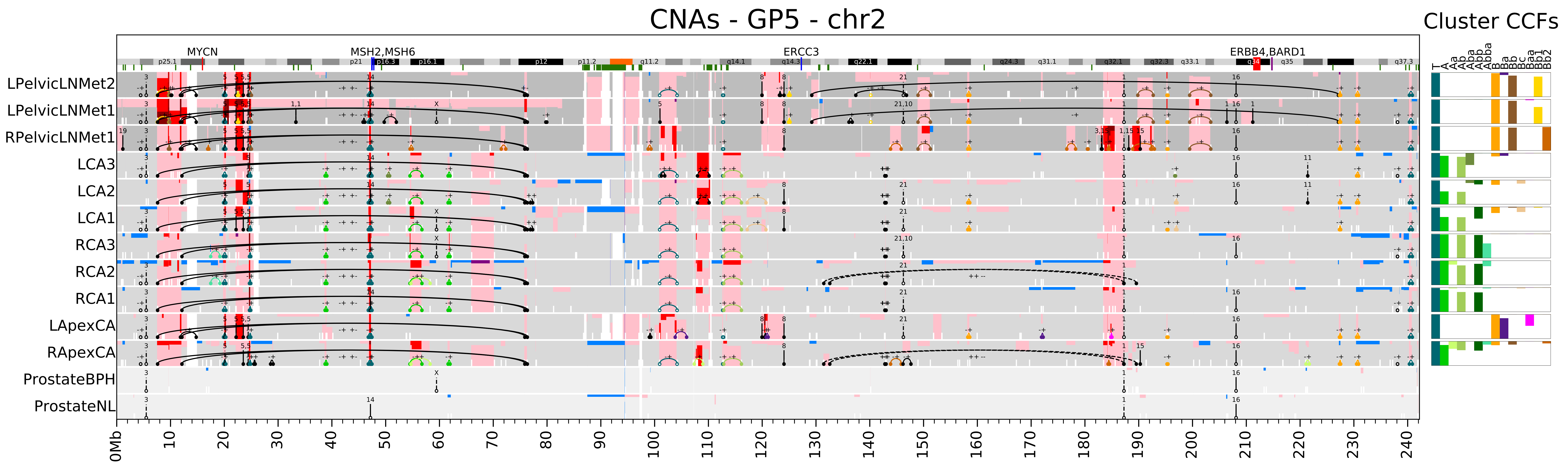
CNAs - GP5 - chr2
Cluster CCFs
MYCN
MSH2,MSH6
ERCC3
ERBB4,BARD1
LPelvicLNMet2
LPelvicLNMet1
RPelvicLNMet1
LCA3
LCA2
LCA1
RCA3
RCA2
RCA1
LApexCA
RApexCA
ProstateBPH
ProstateNL
0Mb
10
20
30
40
50
60
70
80
90
100
110
120
130
140
150
160
170
180
190
200
210
220
230
240
T
A
Aa
Ab
Aba
Abb
Abba
B
Ba
Bb
Bc
Baa
Bb1
Bb2

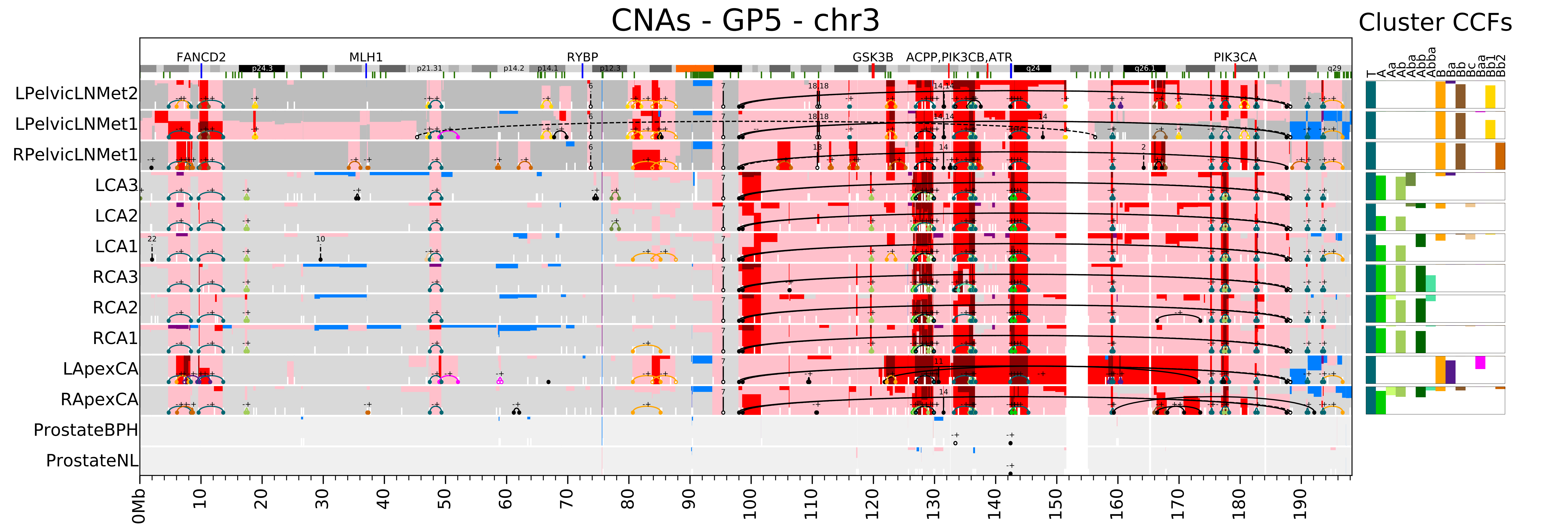

CNAs - GP5 - chr3
Cluster CCFs
FANCD2
MLH1
RYBP
GSK3B
ACPP,PIK3CB,ATR
PIK3CA
LPelvicLNMet2
LPelvicLNMet1
RPelvicLNMet1
LCA3
LCA2
LCA1
RCA3
RCA2
RCA1
LApexCA
RApexCA
ProstateBPH
ProstateNL
0Mb
10
20
30
40
50
60
70
80
90
100
110
120
130
140
150
160
170
180
190
T
A
Aa
Ab
Aba
Abb
Abba
B
Ba
Bb
Bc
Baa
Bb1
Bb2

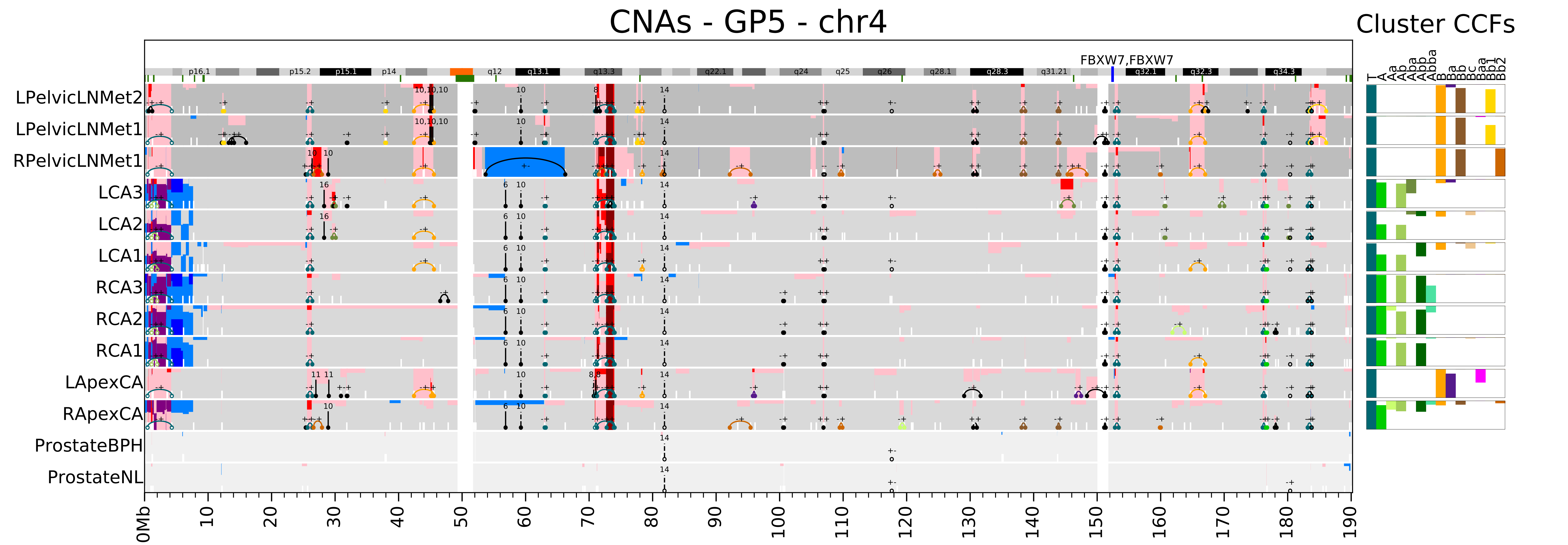
CNAs - GP5 - chr4
Cluster CCFs
FBXW7,FBXW7
LPelvicLNMet2
LPelvicLNMet1
RPelvicLNMet1
LCA3
LCA2
LCA1
RCA3
RCA2
RCA1
LApexCA
RApexCA
ProstateBPH
ProstateNL
T
A
Aa
Ab
Aba
Abb
Abba
B
Ba
Bb
Bc
Baa
Bb1
Bb2
0Mb
10
20
30
40
50
60
70
80
90
100
110
120
130
140
150
160
170
180
190

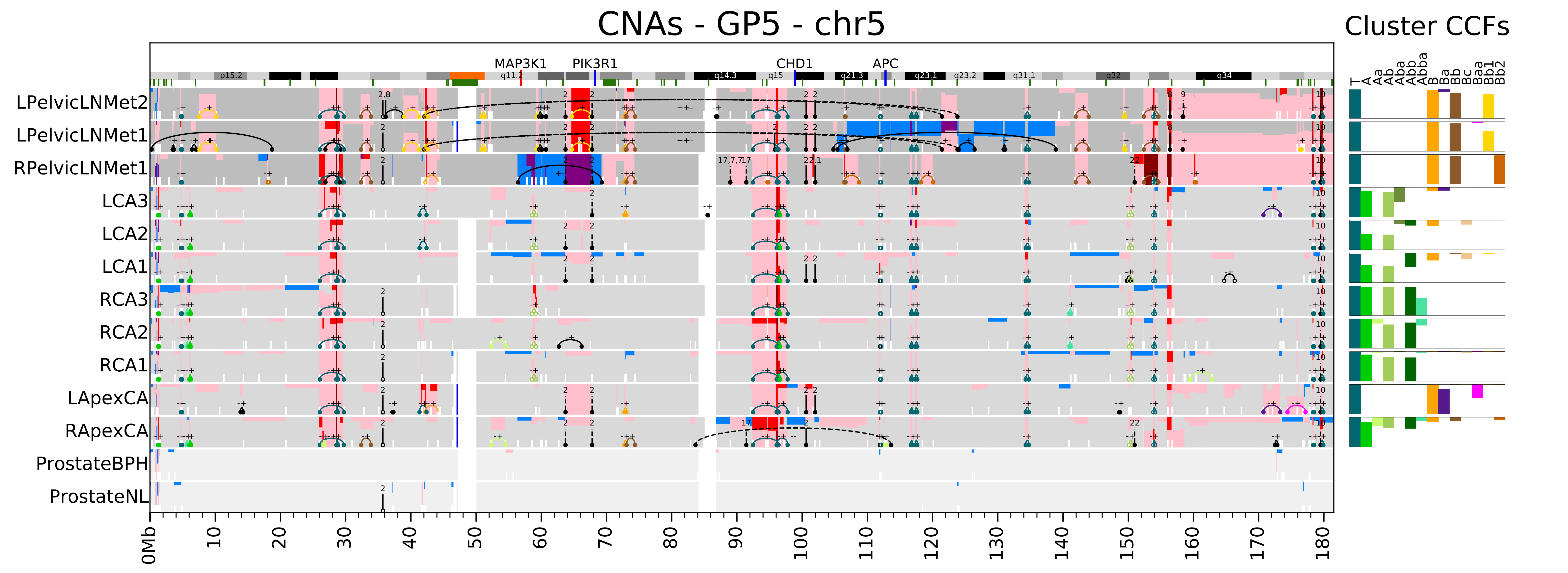

CNAs - GP5 - chr5
Cluster CCFs
MAP3K1
PIK3R1
CHD1
APC
LPelvicLNMet2
LPelvicLNMet1
RPelvicLNMet1
LCA3
LCA2
LCA1
RCA3
RCA2
RCA1
LApexCA
RApexCA
ProstateBPH
ProstateNL
0Mb
10
20
30
40
50
60
70
80
90
100
110
120
130
140
150
160
170
180
T
A
Aa
Ab
Aba
Abb
Abba
B
Ba
Bb
Bc
Baa
Bb1
Bb2

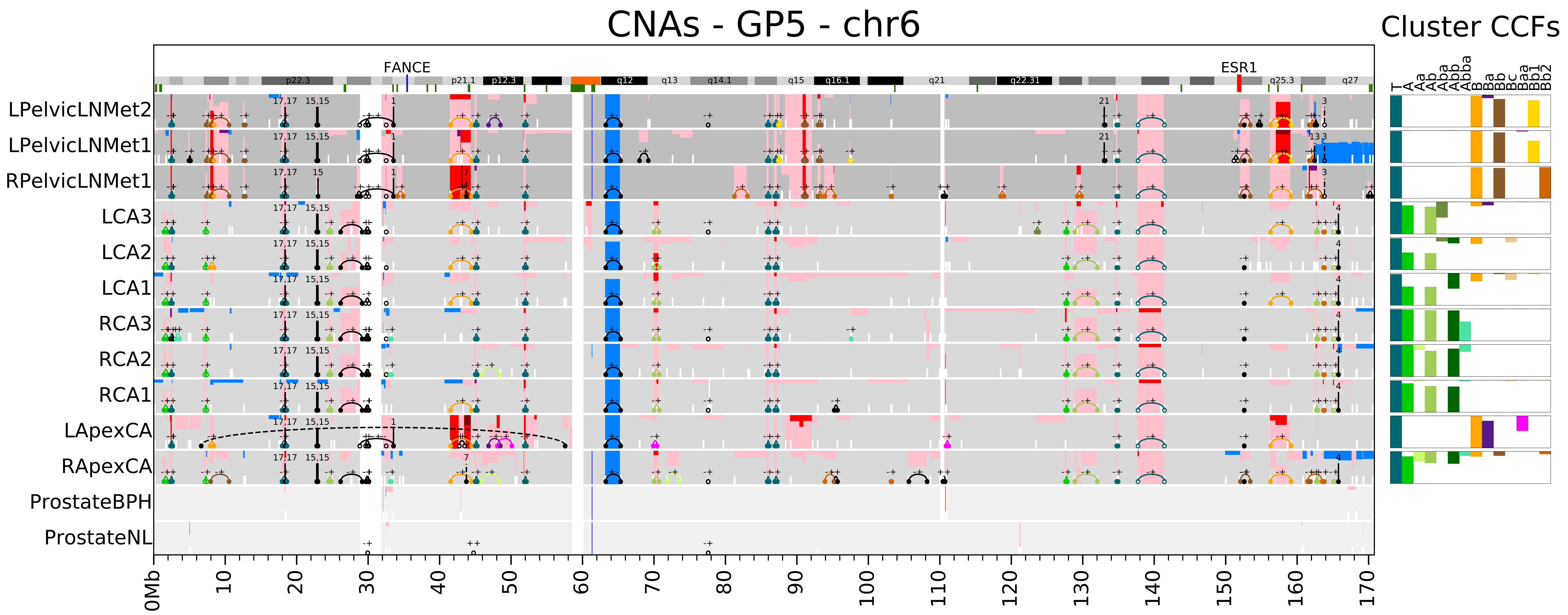

CNAs - GP5 - chr6
Cluster CCFs
FANCE
ESR1
LPelvicLNMet2
LPelvicLNMet1
RPelvicLNMet1
LCA3
LCA2
LCA1
RCA3
RCA2
RCA1
LApexCA
RApexCA
ProstateBPH
ProstateNL
0Mb
10
20
30
40
50
60
70
80
90
100
110
120
130
140
150
160
170
T
A
Aa
Ab
Aba
Abb
Abba
B
Ba
Bb
Bc
Baa
Bb1
Bb2

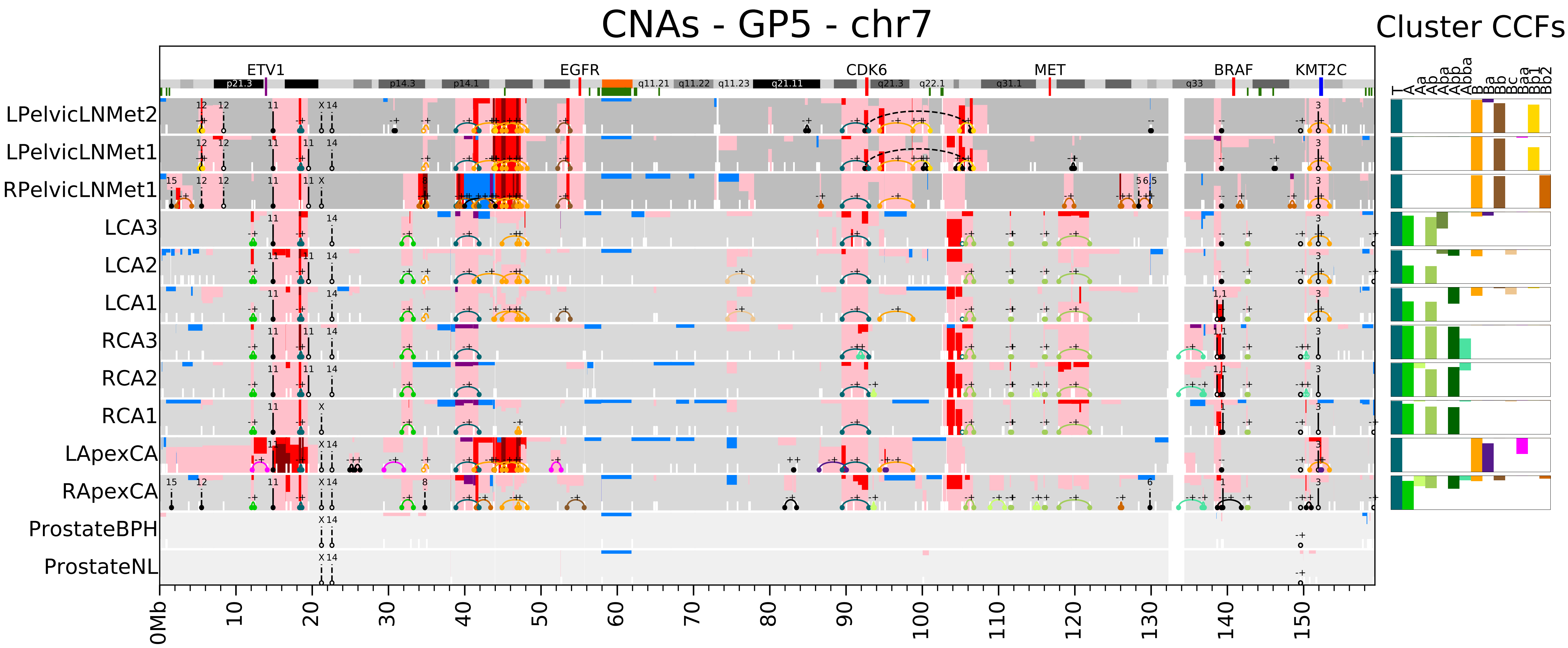

CNAs - GP5 - chr7
Cluster CCFs
ETV1
EGFR
CDK6
MET
BRAF
KMT2C
LPelvicLNMet2
LPelvicLNMet1
RPelvicLNMet1
LCA3
LCA2
LCA1
RCA3
RCA2
RCA1
LApexCA
RApexCA
ProstateBPH
ProstateNL
0Mb
10
20
30
40
50
60
70
80
90
100
110
120
130
140
150
T
A
Aa
Ab
Aba
Abb
Abba
B
Ba
Bb
Bc
Baa
Bb1
Bb2

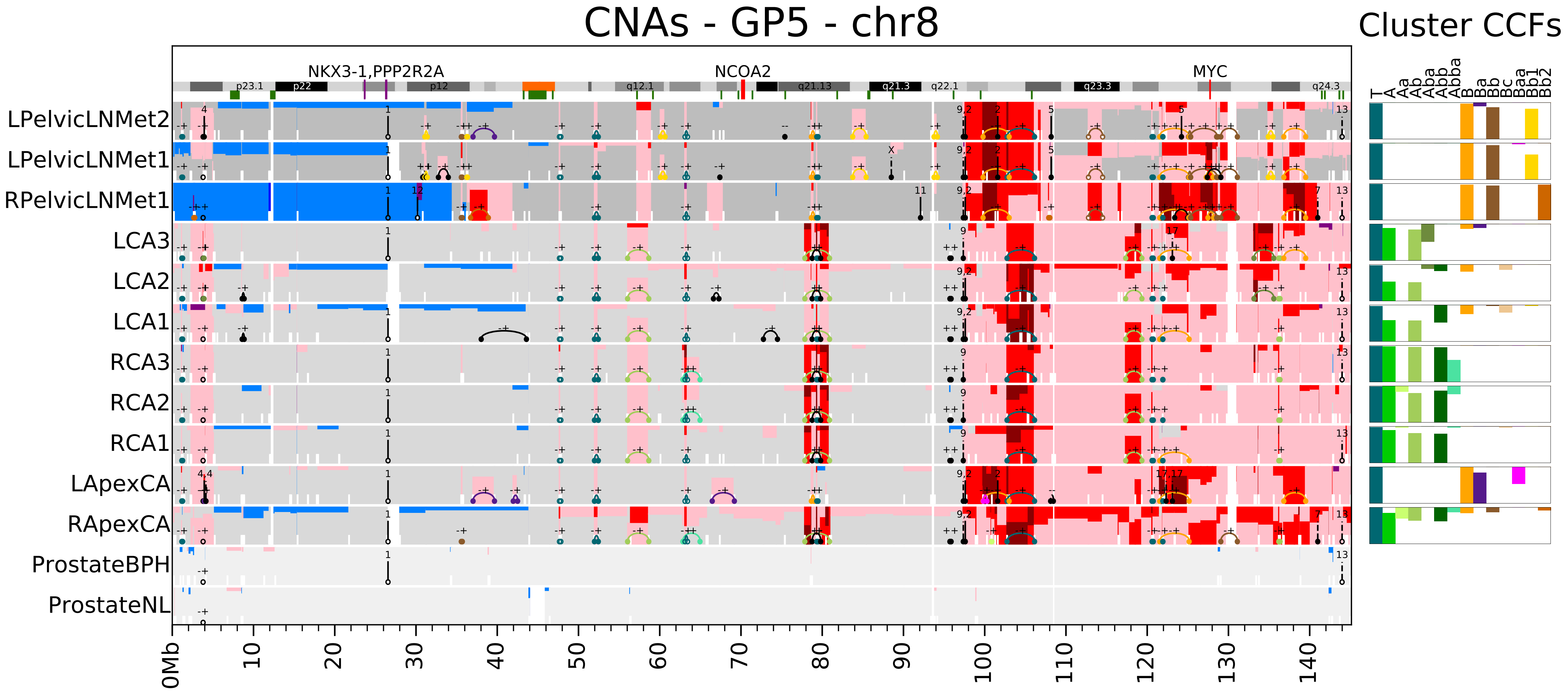
CNAs - GP5 - chr8
Cluster CCFs
NKX3-1,PPP2R2A
NCOA2
MYC
LPelvicLNMet2
LPelvicLNMet1
RPelvicLNMet1
LCA3
LCA2
LCA1
RCA3
RCA2
RCA1
LApexCA
RApexCA
ProstateBPH
ProstateNL
0Mb
10
20
30
40
50
60
70
80
90
100
110
120
130
140
T
A
Aa
Ab
Aba
Abb
Abba
B
Ba
Bb
Bc
Baa
Bb1
Bb2

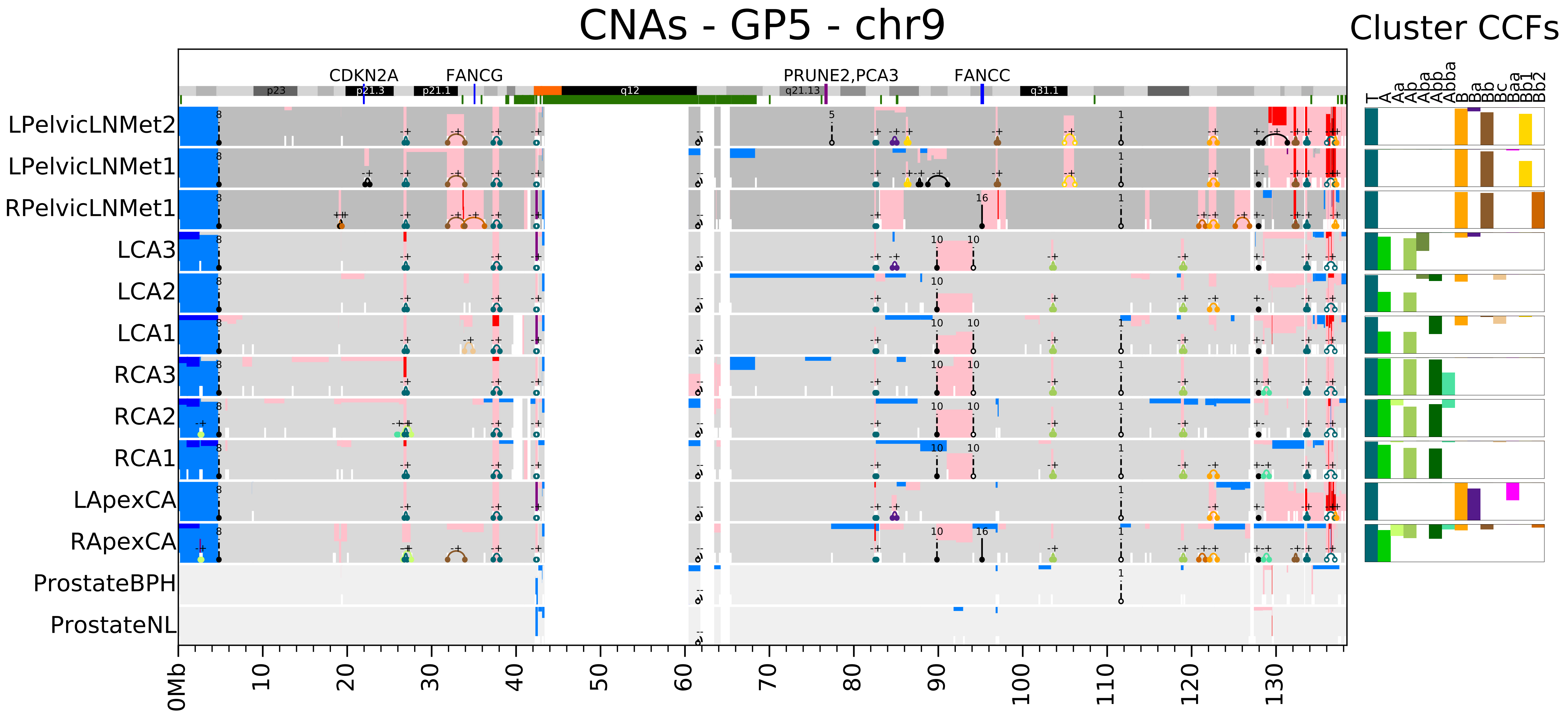
CNAs - GP5 - chr9
Cluster CCFs
CDKN2A
FANCG
PRUNE2,PCA3
FANCC
p23
p21.3
p21.1
q12
q21.13
q31.1
LPelvicLNMet2
LPelvicLNMet1
RPelvicLNMet1
LCA3
LCA2
LCA1
RCA3
RCA2
RCA1
LApexCA
RApexCA
ProstateBPH
ProstateNL
0Mb
10
20
30
40
50
60
70
80
90
100
110
120
130
T
A
Aa
Ab
Aba
Abb
Abba
B
Ba
Bb
Bc
Baa
Bb1
Bb2

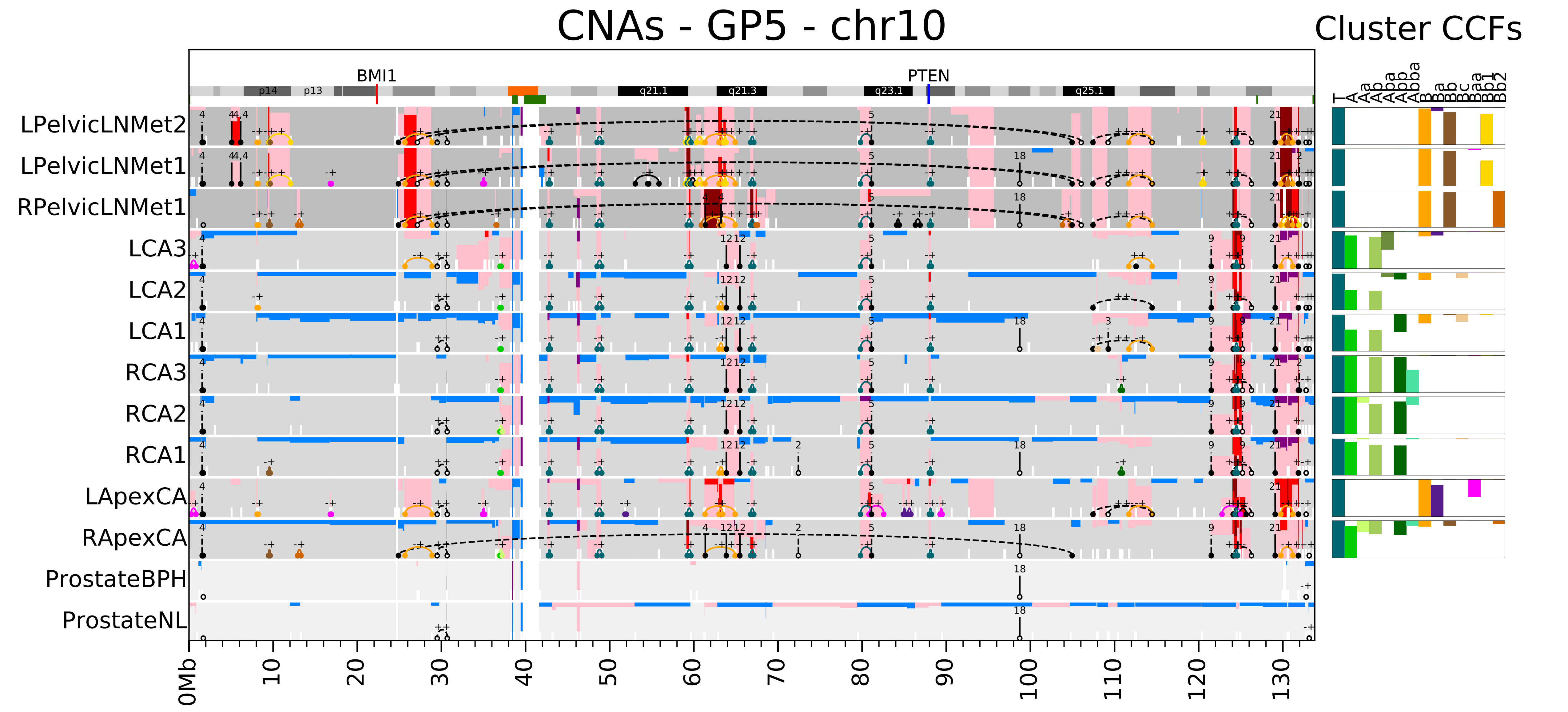

CNAs - GP5 - chr10
Cluster CCFs
BMI1
PTEN
p14
p13
q21.1
q21.3
q23.1
q25.1
LPelvicLNMet2
LPelvicLNMet1
RPelvicLNMet1
LCA3
LCA2
LCA1
RCA3
RCA2
RCA1
LApexCA
RApexCA
ProstateBPH
ProstateNL
T
A
Aa
Ab
Aba
Abb
Abba
B
Ba
Bb
Bc
Baa
Bb1
Bb2
0Mb
10
20
30
40
50
60
70
80
90
100
110
120
130

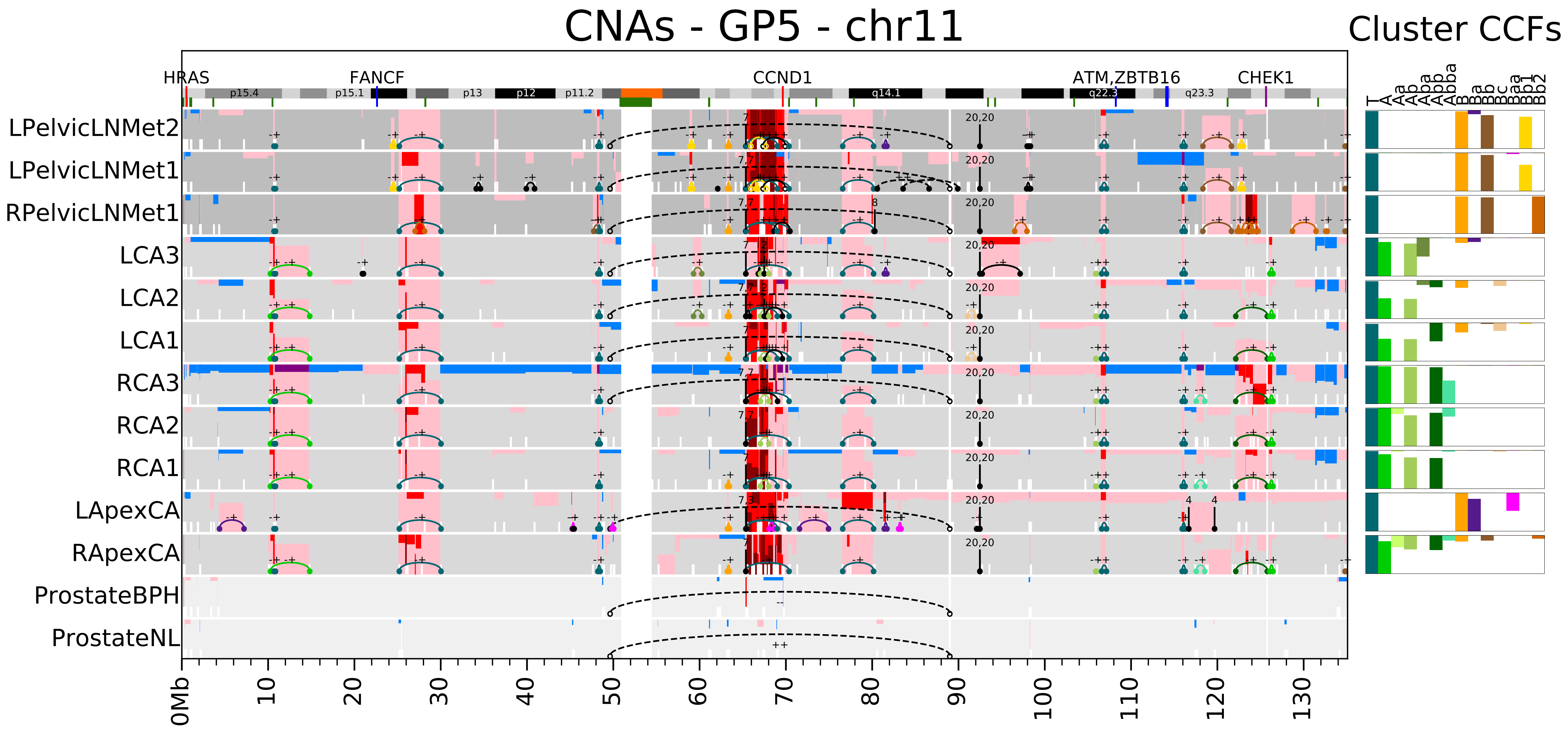
CNAs - GP5 - chr11
Cluster CCFs
HRAS
FANCF
CCND1
ATM,ZBTB16
CHEK1
p15.4
p15.1
p13
p12
p11.2
q14.1
q22.3
q23.3
LPelvicLNMet2
LPelvicLNMet1
RPelvicLNMet1
LCA3
LCA2
LCA1
RCA3
RCA2
RCA1
LApexCA
RApexCA
ProstateBPH
ProstateNL
T
A
Aa
Ab
Aba
Abb
Abba
B
Ba
Bb
Bc
Baa
Bb1
Bb2
0Mb
10
20
30
40
50
60
70
80
90
100
110
120
130

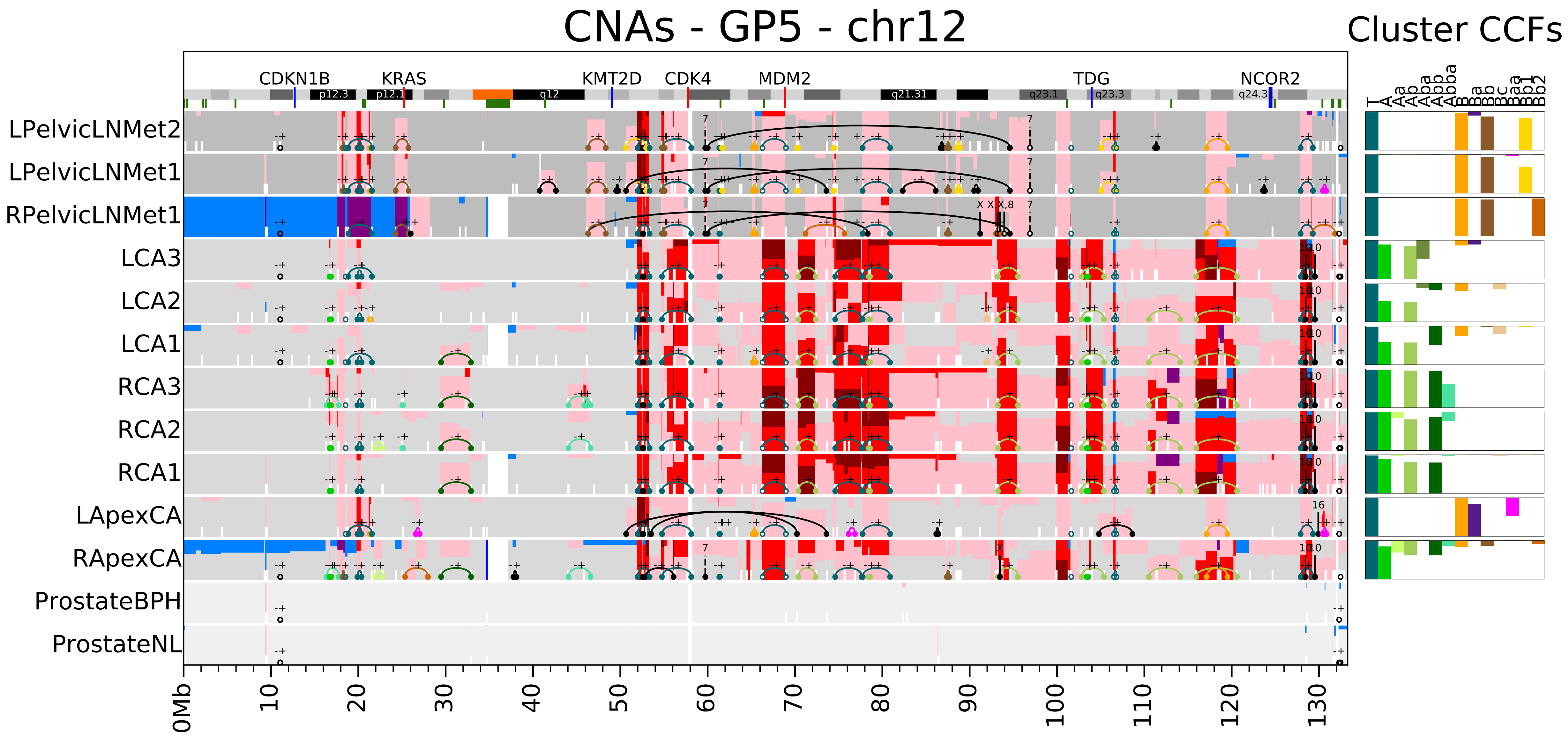

CNAs - GP5 - chr12
Cluster CCFs
CDKN1B
KRAS
KMT2D
CDK4
MDM2
TDG
NCOR2
p12.3
p12.1
q12
q21.31
q23.1
q23.3
q24.31
LPelvicLNMet2
LPelvicLNMet1
RPelvicLNMet1
LCA3
LCA2
LCA1
RCA3
RCA2
RCA1
LApexCA
RApexCA
ProstateBPH
ProstateNL
0Mb
10
20
30
40
50
60
70
80
90
100
110
120
130
T
A
Aa
Ab
Aba
Abb
Abba
B
Ba
Bb
Bc
Baa
Bb1
Bb2

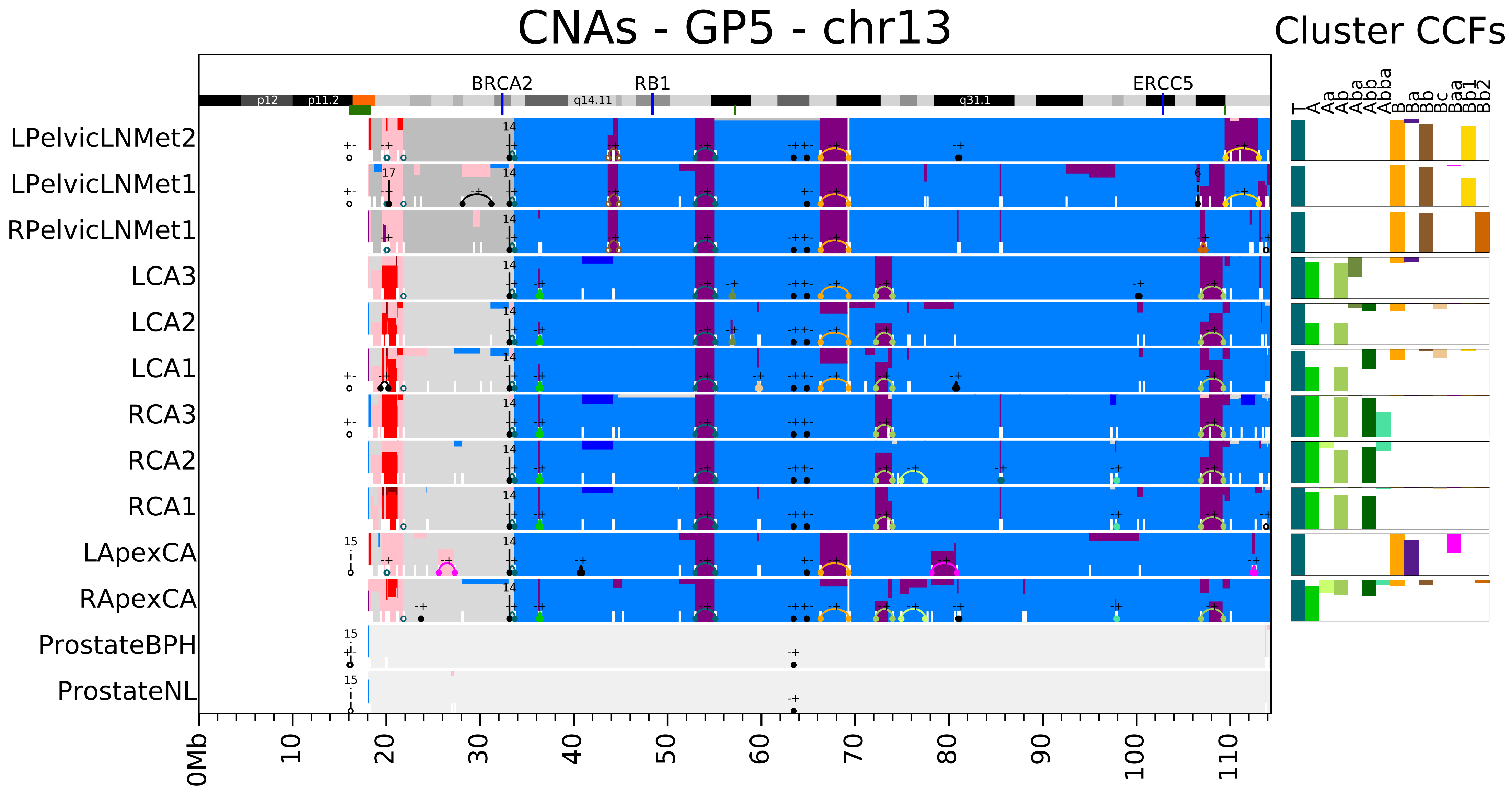

CNAs - GP5 - chr13
Cluster CCFs
BRCA2
RB1
ERCC5
p12
p11.2
q14.11
q31.1
LPelvicLNMet2
LPelvicLNMet1
RPelvicLNMet1
LCA3
LCA2
LCA1
RCA3
RCA2
RCA1
LApexCA
RApexCA
ProstateBPH
ProstateNL
T
A
Aa
Ab
Aba
Abb
Abba
B
Ba
Bb
Bc
Baa
Bb1
Bb2
0Mb
10
20
30
40
50
60
70
80
90
100
110

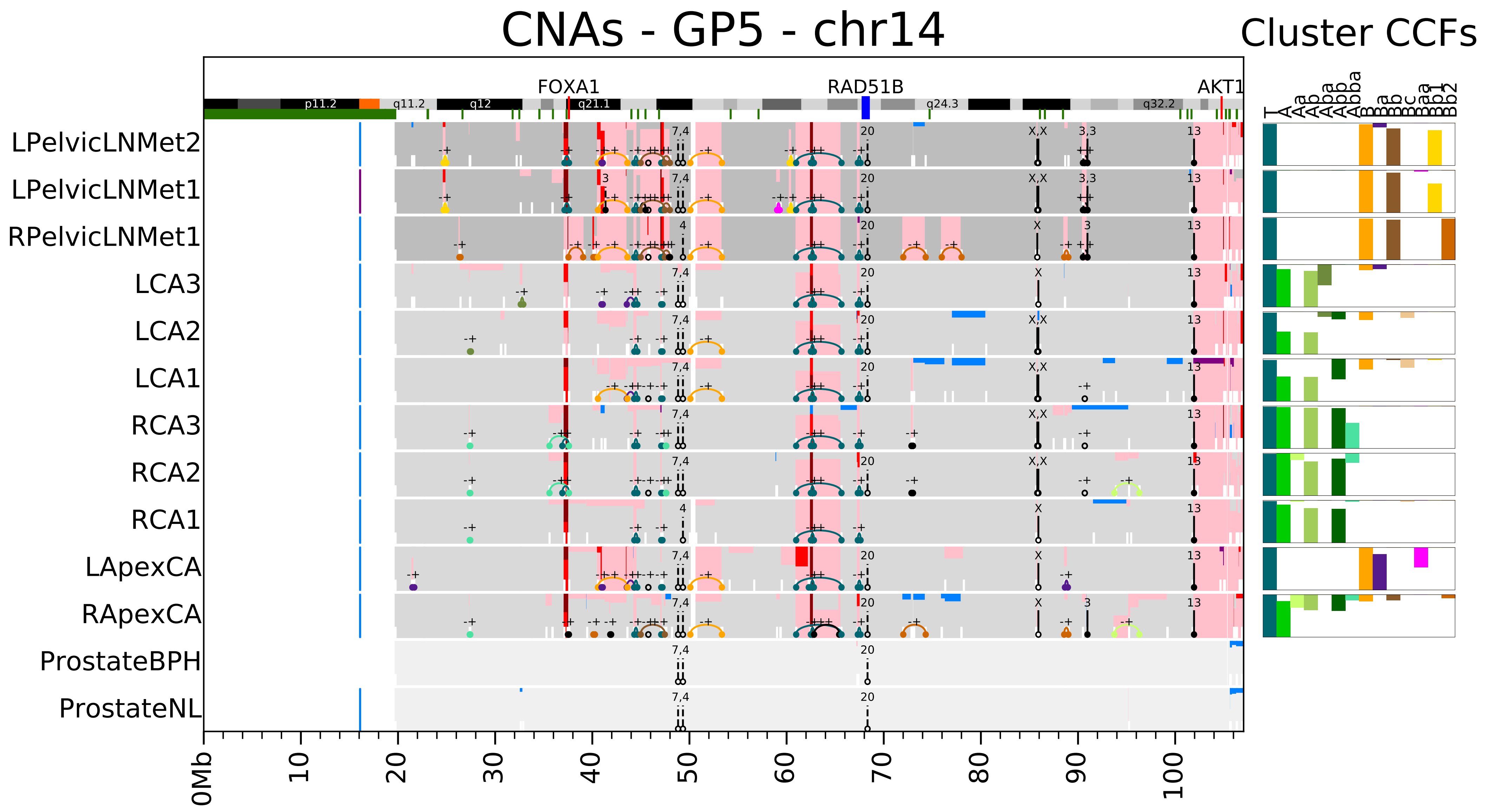

CNAs - GP5 - chr14
Cluster CCFs
FOXA1
RAD51B
AKT1
p11.2
q11.2
q12
q21.1
q24.3
q32.2
LPelvicLNMet2
LPelvicLNMet1
RPelvicLNMet1
LCA3
LCA2
LCA1
RCA3
RCA2
RCA1
LApexCA
RApexCA
ProstateBPH
ProstateNL
0Mb
10
20
30
40
50
60
70
80
90
100
T
A
Aa
Ab
Aba
Abb
Abba
B
Ba
Bb
Bc
Baa
Bb1
Bb2

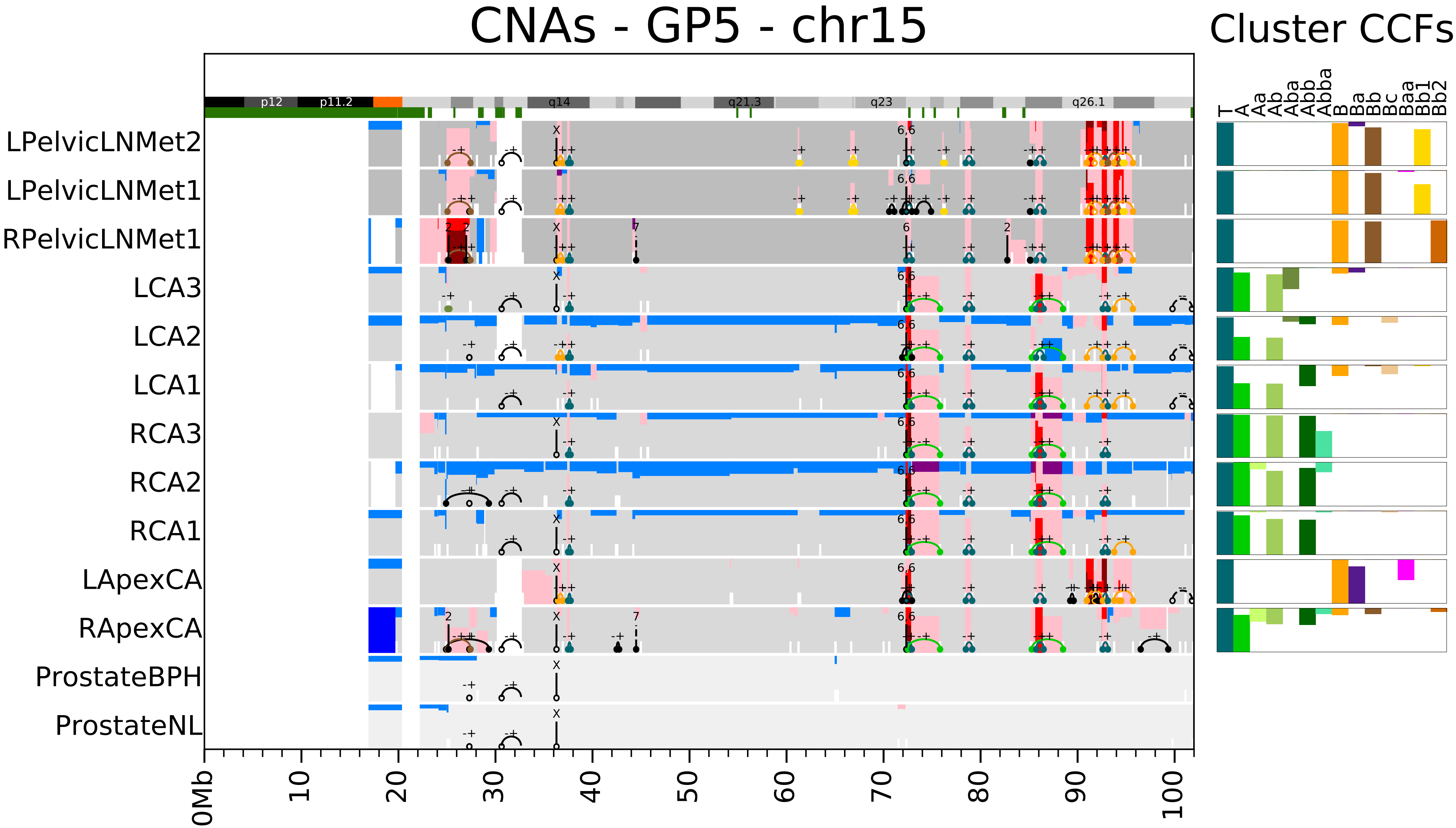

CNAs - GP5 - chr15
Cluster CCFs
p12
p11.2
q14
q21.3
q23
q26.1
LPelvicLNMet2
LPelvicLNMet1
RPelvicLNMet1
LCA3
LCA2
LCA1
RCA3
RCA2
RCA1
LApexCA
RApexCA
ProstateBPH
ProstateNL
0Mb
10
20
30
40
50
60
70
80
90
100
T
A
Aa
Ab
Aba
Abb
Abba
B
Ba
Bb
Bc
Baa
Bb1
Bb2

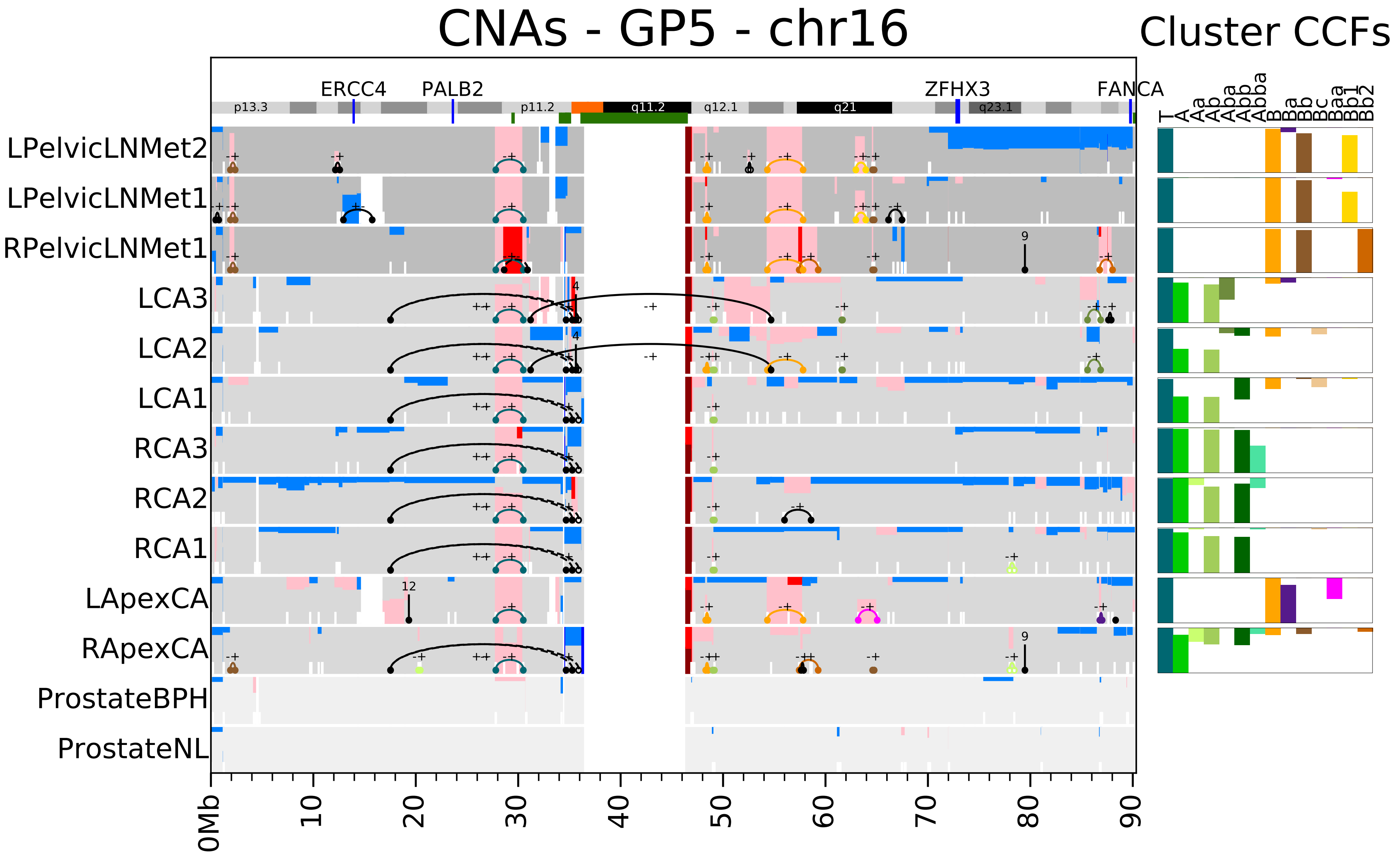

CNAs - GP5 - chr16
Cluster CCFs
ERCC4
PALB2
ZFHX3
FANCA
p13.3
p11.2
q11.2
q12.1
q21
q23.1
LPelvicLNMet2
LPelvicLNMet1
RPelvicLNMet1
LCA3
LCA2
LCA1
RCA3
RCA2
RCA1
LApexCA
RApexCA
ProstateBPH
ProstateNL
T
A
Aa
Ab
Aba
Abb
Abba
B
Ba
Bb
Bc
Baa
Bb1
Bb2
0Mb
10
20
30
40
50
60
70
80
90

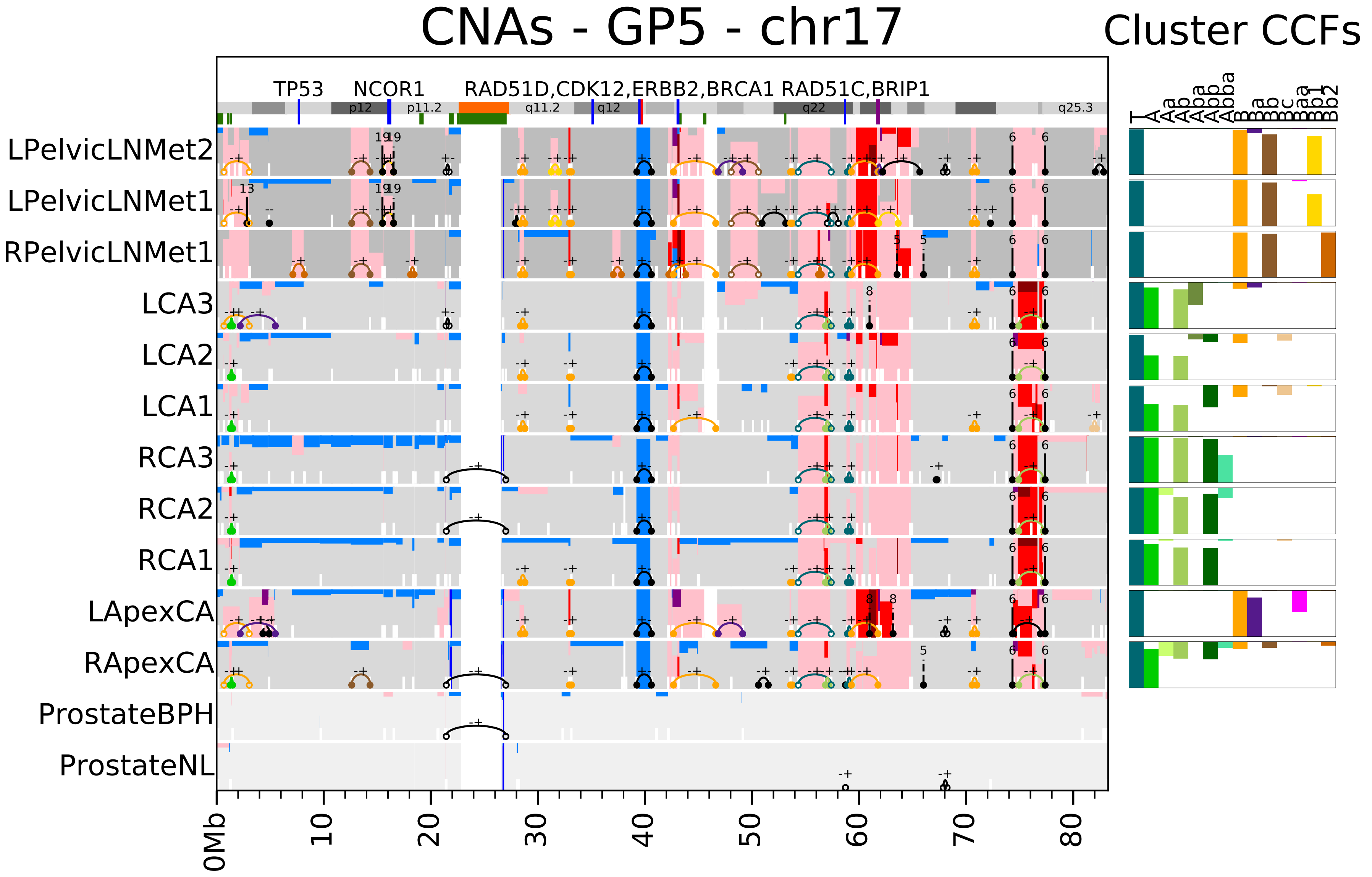

CNAs - GP5 - chr17
Cluster CCFs
TP53
NCOR1
RAD51D,CDK12,ERBB2,BRCA1
RAD51C,BRIP1
p12
p11.2
q11.2
q12
q22
q25.3
LPelvicLNMet2
LPelvicLNMet1
RPelvicLNMet1
LCA3
LCA2
LCA1
RCA3
RCA2
RCA1
LApexCA
RApexCA
ProstateBPH
ProstateNL
0Mb
10
20
30
40
50
60
70
80
T
A
Aa
Ab
Aba
Abba
B
Ba
Bb
Bc
Baa
Bb1
Bb2

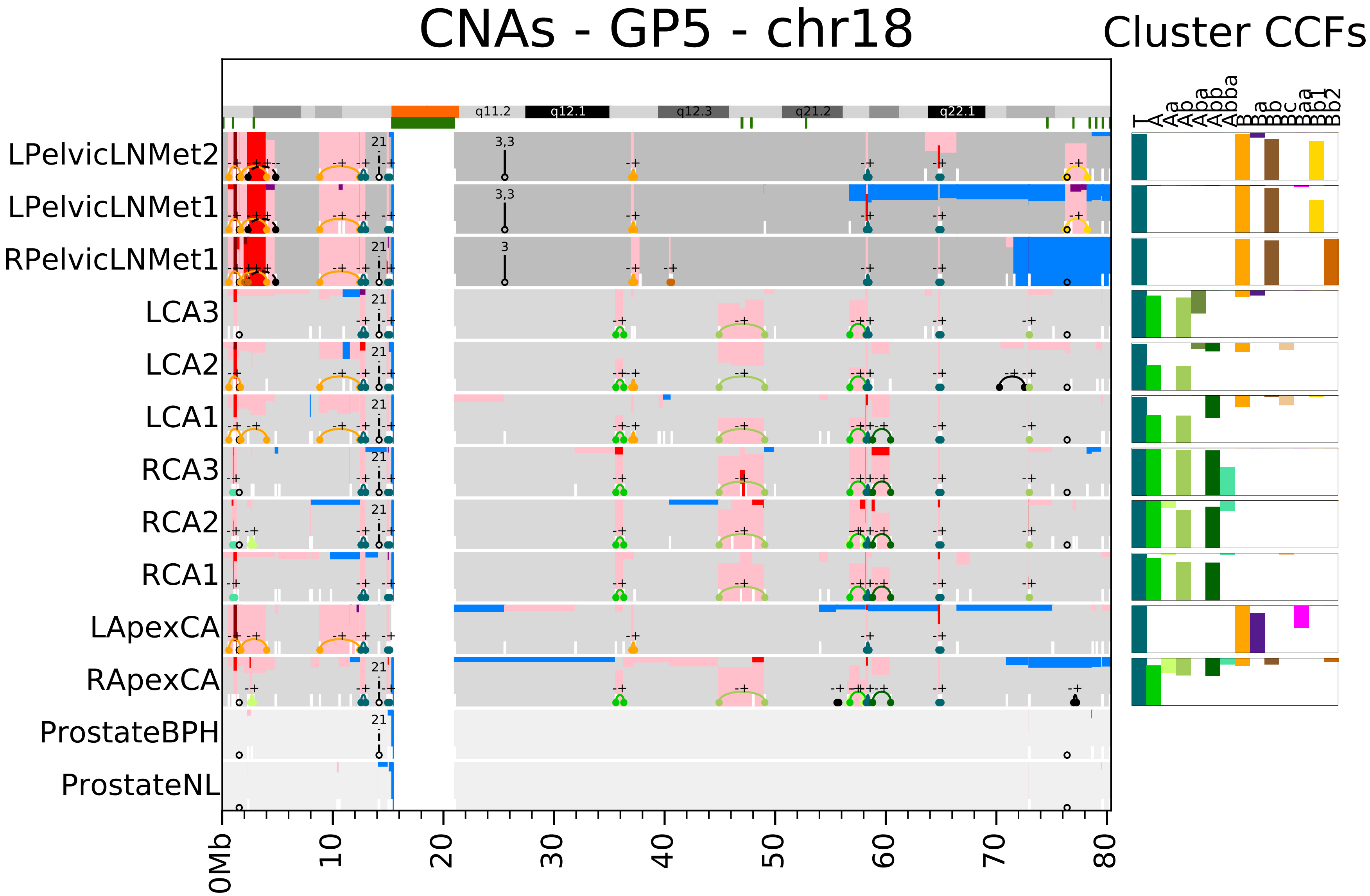

CNAs - GP5 - chr18
Cluster CCFs
q11.2
q12.1
q12.3
q21.2
q22.1
LPelvicLNMet2
LPelvicLNMet1
RPelvicLNMet1
LCA3
LCA2
LCA1
RCA3
RCA2
RCA1
LApexCA
RApexCA
ProstateBPH
ProstateNL
0Mb
10
20
30
40
50
60
70
80
T
A
Aa
Ab
Aba
Abb
Abba
B
Ba
Bb
Bc
Baa
Bb1
Bb2

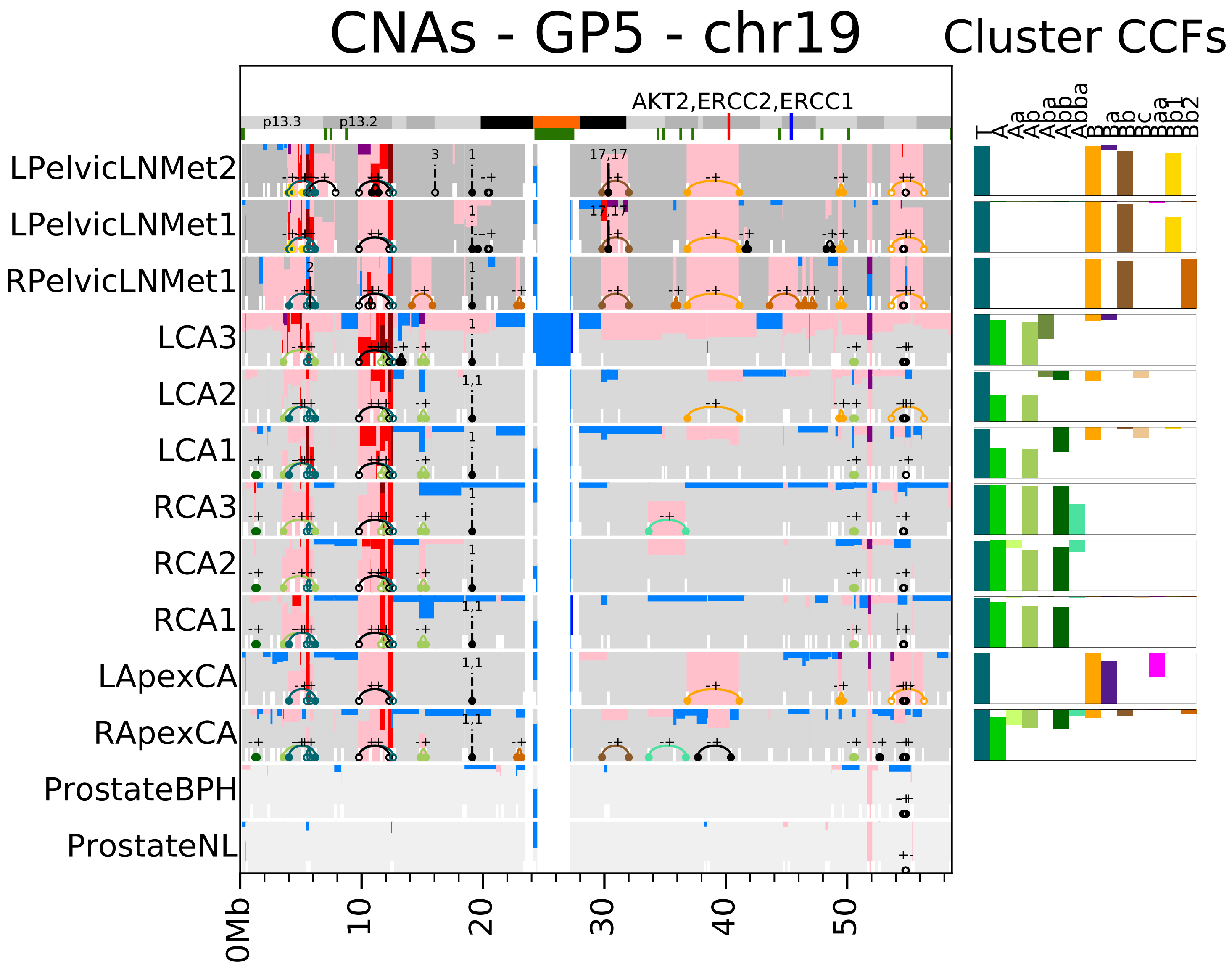
CNAs - GP5 - chr19
Cluster CCFs
AKT2,ERCC2,ERCC1
p13.3
p13.2
LPelvicLNMet2
LPelvicLNMet1
RPelvicLNMet1
LCA3
LCA2
LCA1
RCA3
RCA2
RCA1
LApexCA
RApexCA
ProstateBPH
ProstateNL
0Mb
10
20
30
40
50
T
A
Aa
Ab
Aba
Abb
Abba
B
Ba
Bb
Bc
Baa
Bb1
Bb2

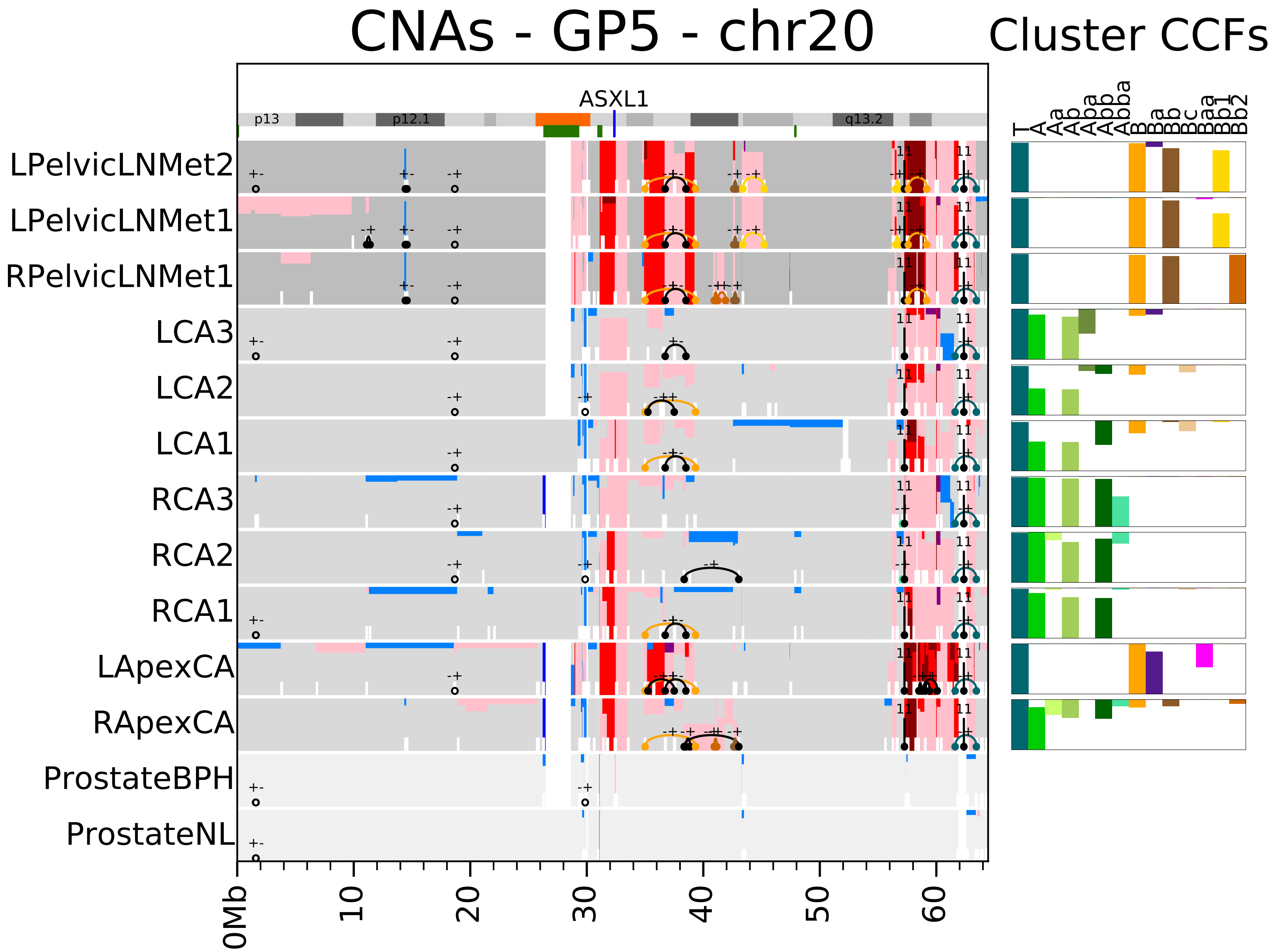

CNAs - GP5 - chr20
Cluster CCFs
ASXL1
p13
p12.1
q13.2
LPelvicLNMet2
LPelvicLNMet1
RPelvicLNMet1
LCA3
LCA2
LCA1
RCA3
RCA2
RCA1
LApexCA
RApexCA
ProstateBPH
ProstateNL
0Mb
10
20
30
40
50
60
T
A
Aa
Ab
Aba
Abb
Abba
B
Ba
Bb
Bc
Baa
Bb1
Bb2

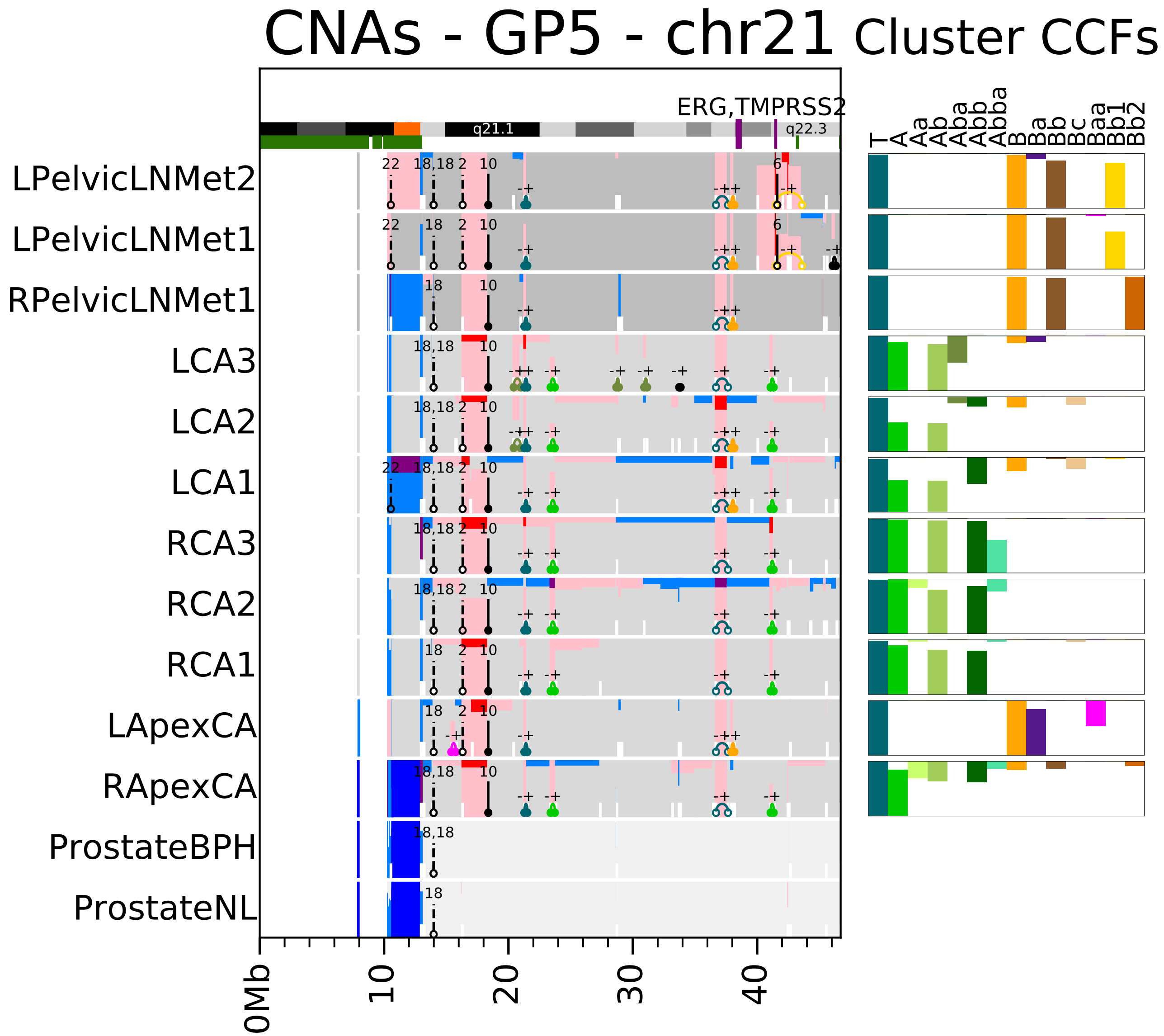

CNAs - GP5 - chr21
Cluster CCFs
ERG,TMPRSS2
q21.1
q22.3
LPelvicLNMet2
LPelvicLNMet1
RPelvicLNMet1
LCA3
LCA2
LCA1
RCA3
RCA2
RCA1
LApexCA
RApexCA
ProstateBPH
ProstateNL
0Mb
10
20
30
40
T
A
Aa
Ab
Aba
Abb
Abba
B
Ba
Bb
Bc
Baa
Bb1
Bb2

CNAs - GP5 - chr22 Cluster CCFs

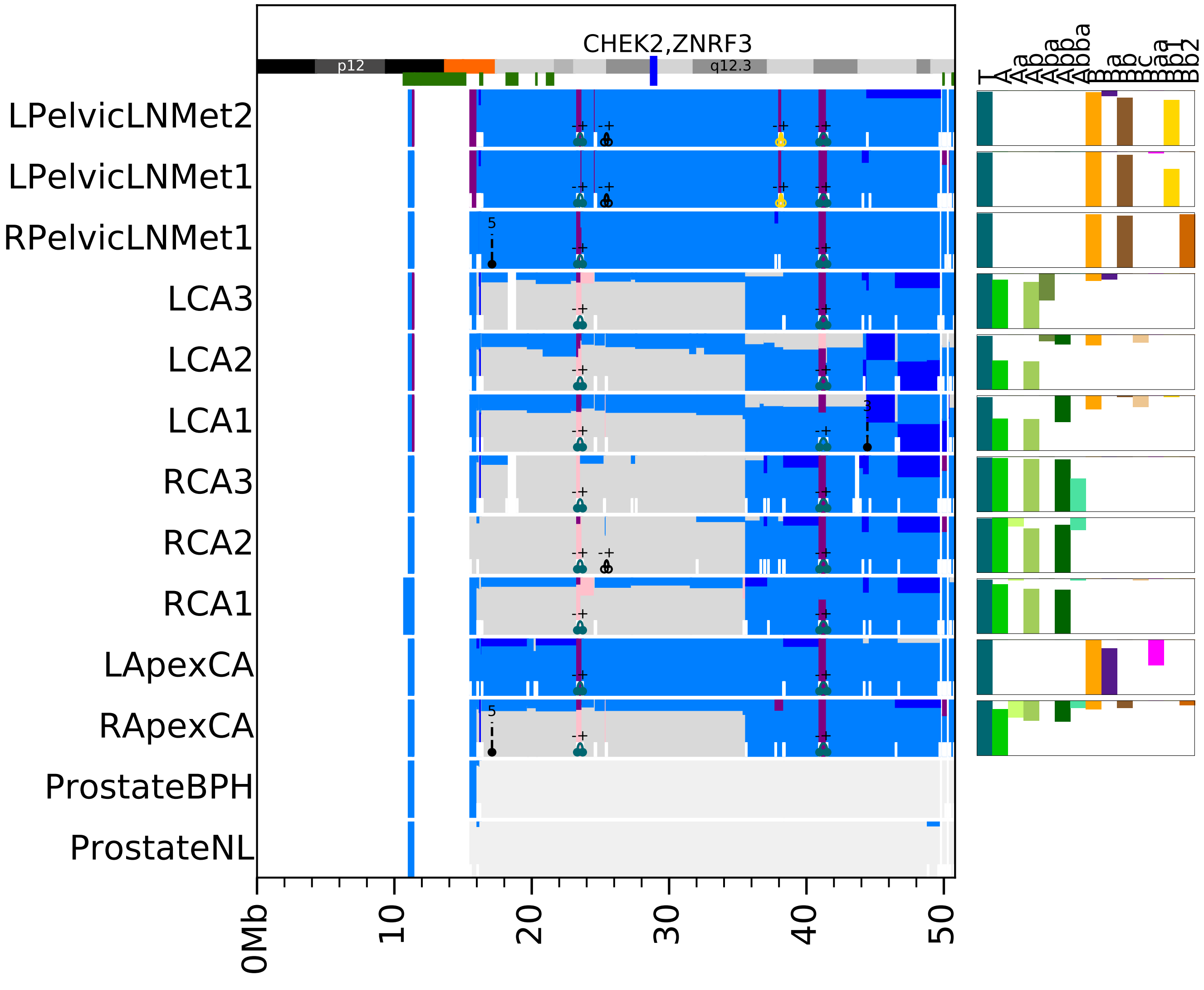

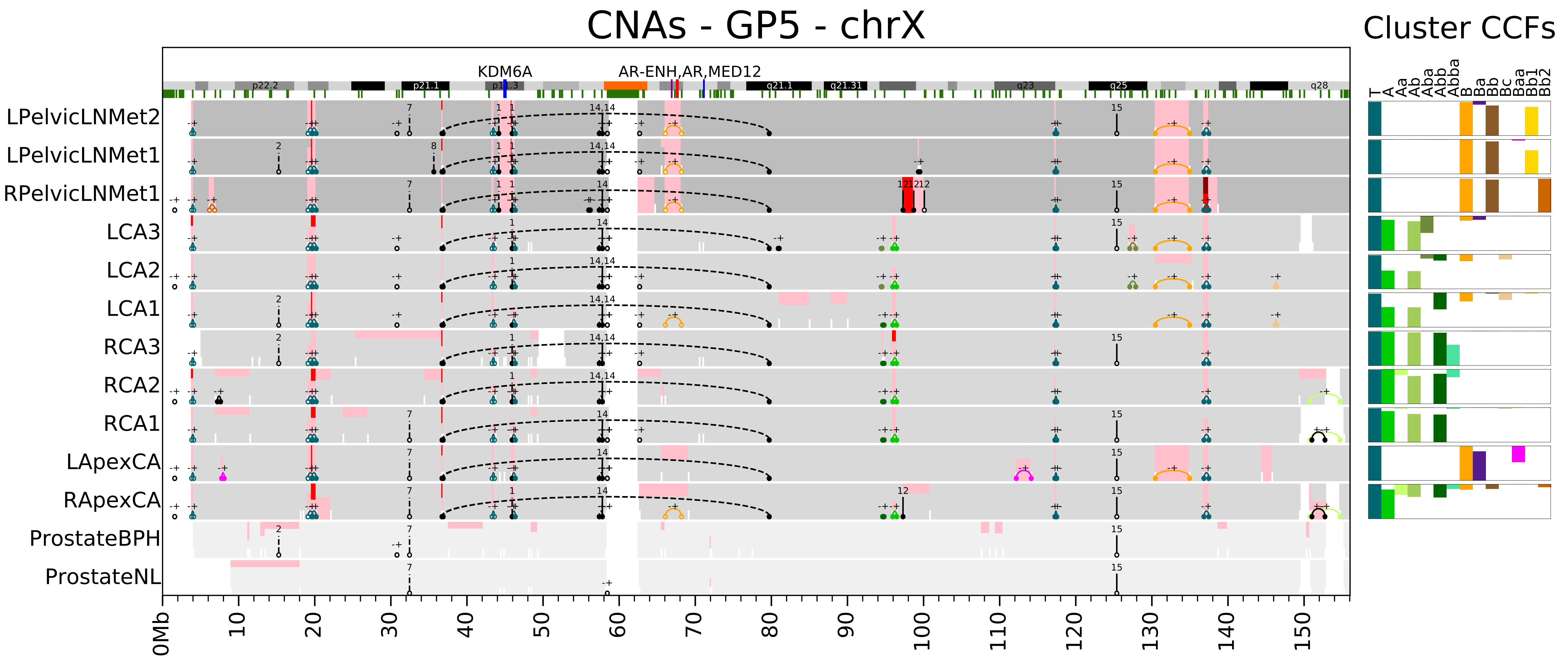

CNAs - GP5 - chrX
Cluster CCFs
KDM6A
AR-ENH,AR,MED12
LPelvicLNMet2
LPelvicLNMet1
RPelvicLNMet1
LCA3
LCA2
LCA1
RCA3
RCA2
RCA1
LApexCA
RApexCA
ProstateBPH
ProstateNL
0Mb
10
20
30
40
50
60
70
80
90
100
110
120
130
140
150
T
A
Aa
Ab
Aba
Abb
Abba
B
Ba
Bb
Bc
Baa
Bb1
Bb2

Fig. S8 GP5 Somatic Tumor DNA Alteration Whole Chromosome Linear Plots. CNAs and structural variants detected in each sample are visualized in their separate horizontal lanes. The fraction of coloring used for the CNAs inside the lanes is proportional to the fraction of cancer cells having the copy number alteration. Fractions below 50% are displayed at the top of the lane while higher fractions are displayed from the bottom up. Prostate cancer -associated genes are shown at the top of the lanes at their genomic coordinates. Centromeres are displayed in orange on the cytoband information graphic. Below the cytoband, green coloring is used for “blacklisted” genomic regions (6) where CNA and SV calls may have low confidence. Evolutionary clusters CCFs are drawn in a separate table on the right side of each chromosome with colored bars proportional to lane height in each sample, so that a colored bar filling the whole lane equals 100% CCF and no bar meaning that the cluster is not detected in the sample. Black dots that do not have a connecting arc represent intrachromosomal breakpoints that are less than 100Kb apart. Tandem duplications (dots connected with solid arcs) are drawn using the color of the cluster they have been assigned to.

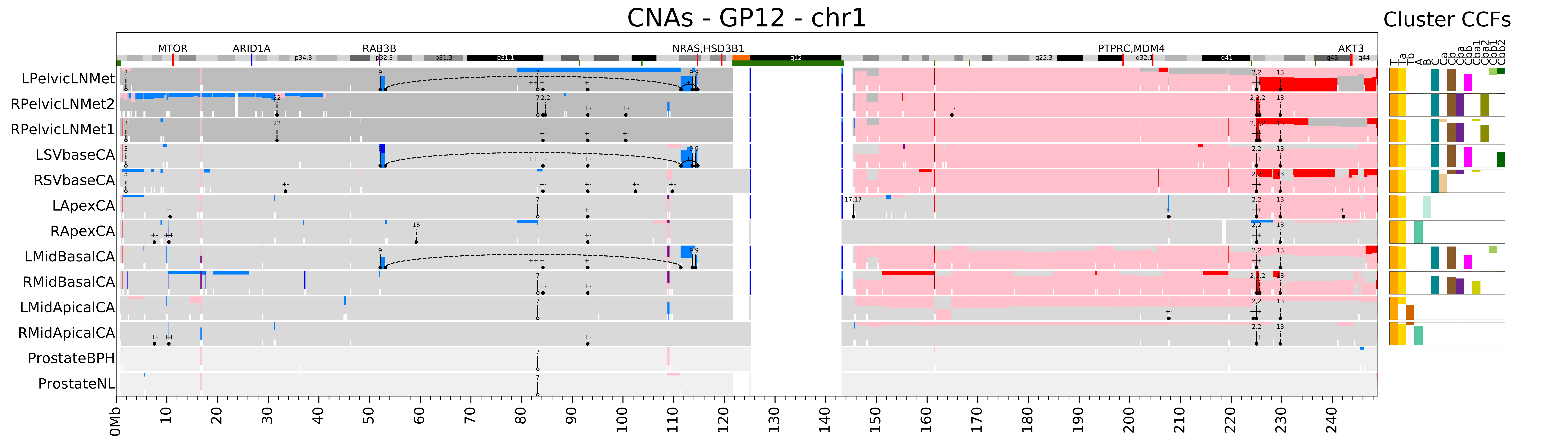

CNAs - GP12 - chr1
Cluster CCFs
MTOR
ARID1A
RAB3B
NRAS,HSD3B1
PTPRC,MDM4
AKT3
LPelvicLNMet
RPelvicLNMet2
RPelvicLNMet1
LSVbaseCA
RSVbaseCA
LApexCA
RApexCA
LMidBasalCA
RMidBasalCA
LMidApicalCA
RMidApicalCA
ProstateBPH
ProstateNL
0Mb
10
20
30
40
50
60
70
80
90
100
110
120
130
140
150
160
170
180
190
200
210
220
230
240
T
Ta
Tb
A
B
C
Ca
Cb
Cba
Cbb
Cba1
Cba2
Cbb1
Cbb2

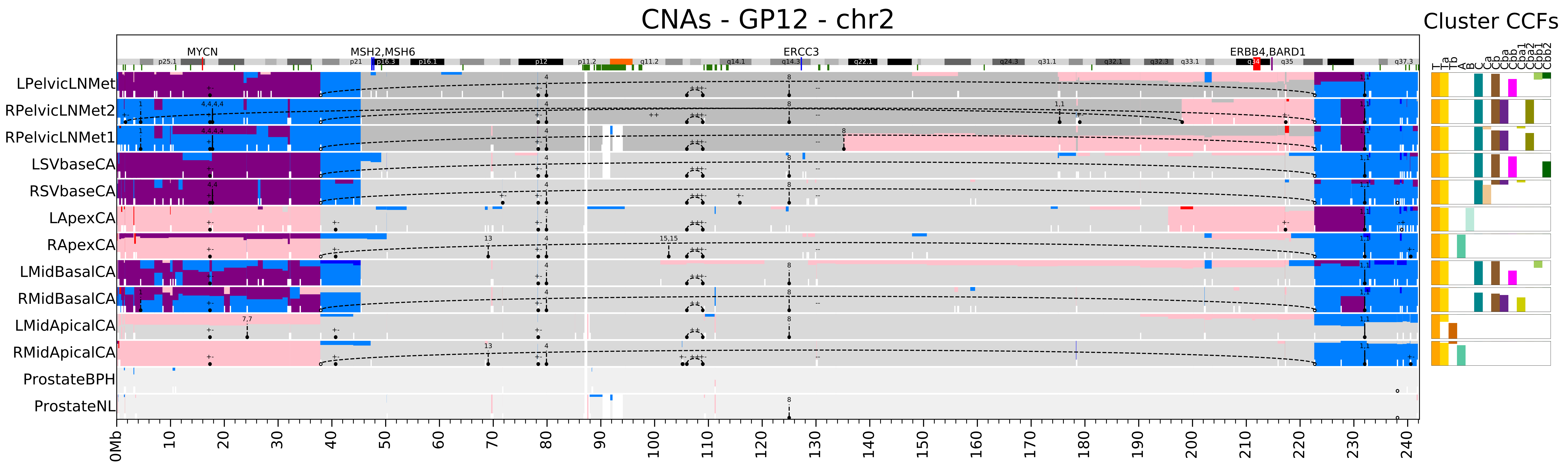
CNAs - GP12 - chr2
Cluster CCFs
MYCN
MSH2,MSH6
ERCC3
ERBB4,BARD1
LPelvicLNMet
RPelvicLNMet2
RPelvicLNMet1
LSVbaseCA
RSVbaseCA
LApexCA
RApexCA
LMidBasalCA
RMidBasalCA
LMidApicalCA
RMidApicalCA
ProstateBPH
ProstateNL
T
Ta
Tb
A
B
C
Ca
Cb
Cba
Cbb
Cba1
Cba2
Cbb1
Cbb2
0Mb
10
20
30
40
50
60
70
80
90
100
110
120
130
140
150
160
170
180
190
200
210
220
230
240

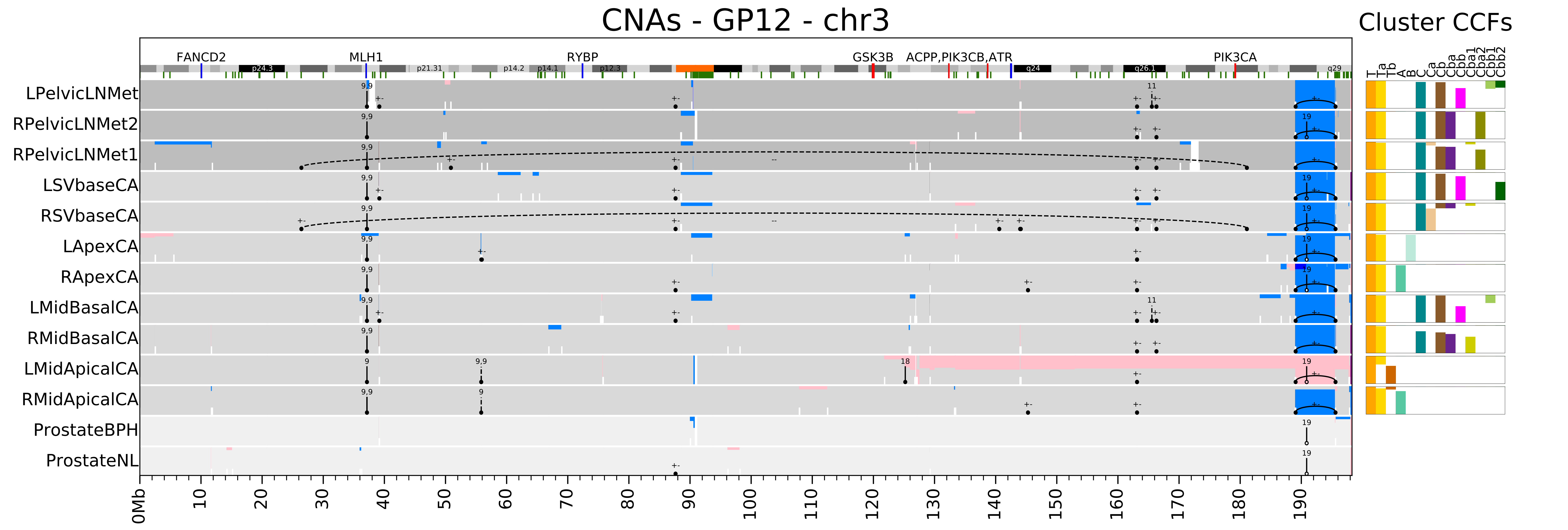

CNAs - GP12 - chr3
Cluster CCFs
FANCD2
MLH1
RYBP
GSK3B
ACPP,PIK3CB,ATR
PIK3CA
LPelvicLNMet
RPelvicLNMet2
RPelvicLNMet1
LSVbaseCA
RSVbaseCA
LApexCA
RApexCA
LMidBasalCA
RMidBasalCA
LMidApicalCA
RMidApicalCA
ProstateBPH
ProstateNL
0Mb
10
20
30
40
50
60
70
80
90
100
110
120
130
140
150
160
170
180
190
T
Ta
Tb
A
B
C
Ca
Cb
Cba
Cbb
Cba1
Cba2
Cbb1
Cbb2

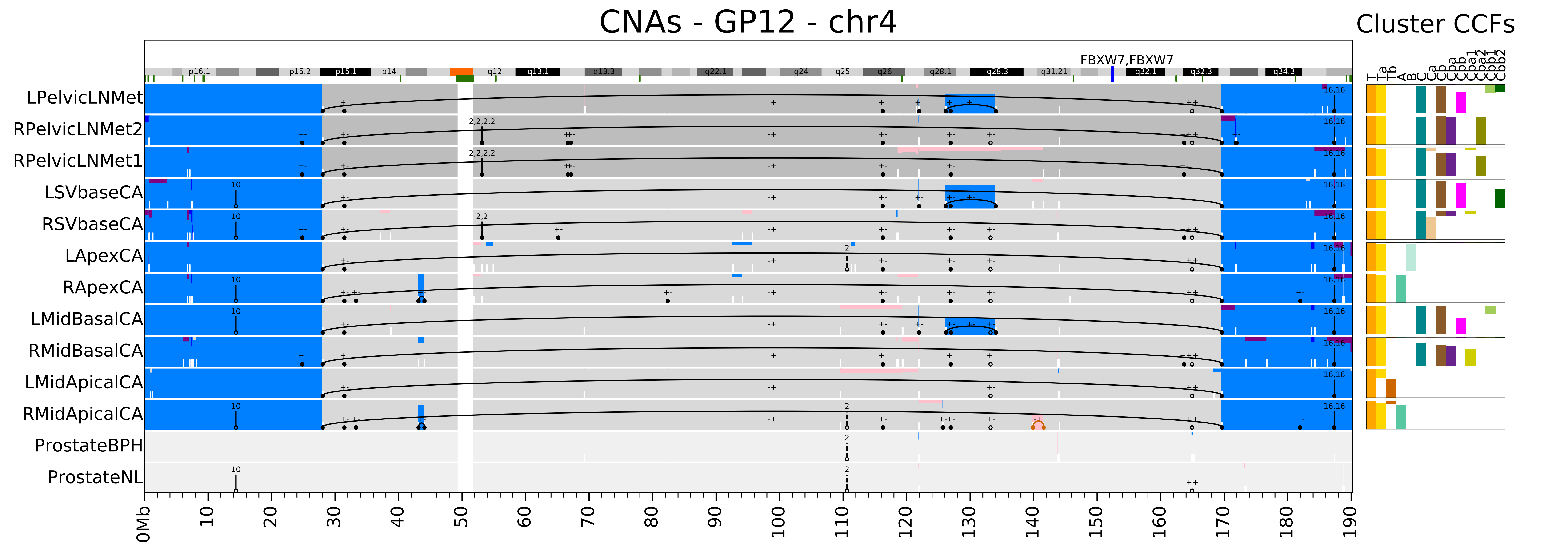

CNAs - GP12 - chr4
Cluster CCFs
FBXW7,FBXW7
LPelvicLNMet
RPelvicLNMet2
RPelvicLNMet1
LSVbaseCA
RSVbaseCA
LApexCA
RApexCA
LMidBasalCA
RMidBasalCA
LMidApicalCA
RMidApicalCA
ProstateBPH
ProstateNL
0Mb
10
20
30
40
50
60
70
80
90
100
110
120
130
140
150
160
170
180
190

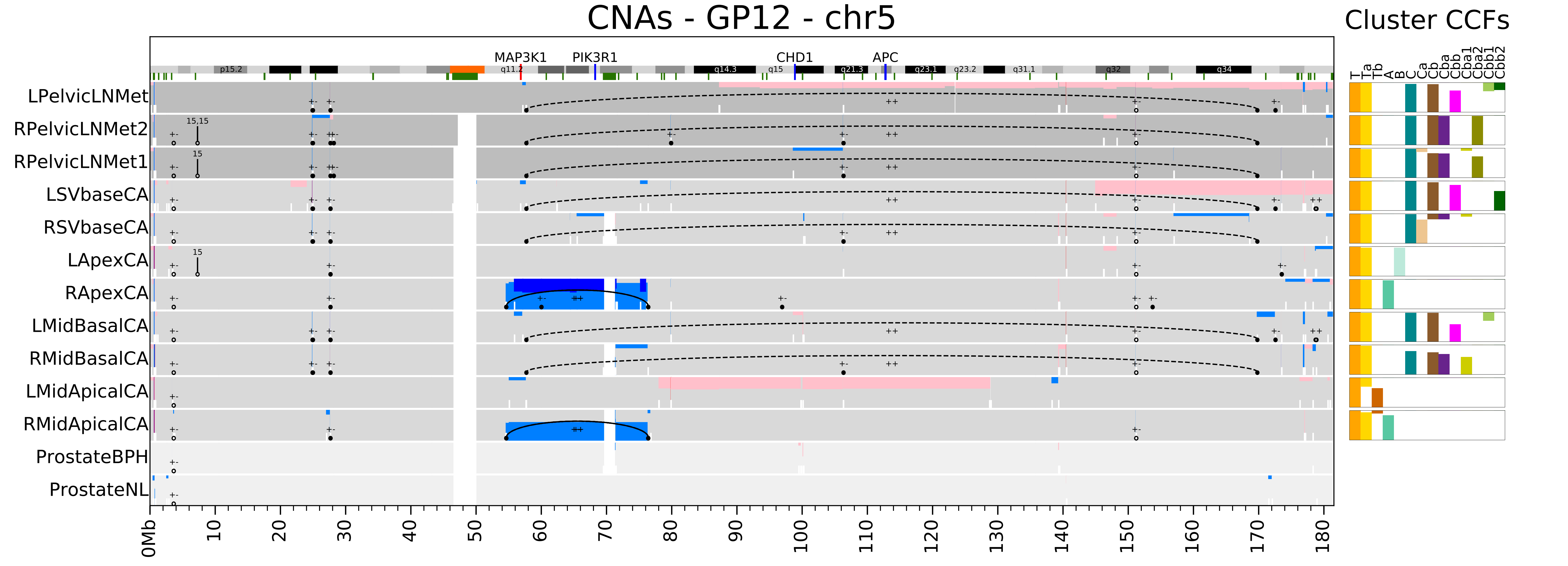

CNAs - GP12 - chr5
Cluster CCFs
MAP3K1
PIK3R1
CHD1
APC
LPelvicLNMet
RPelvicLNMet2
RPelvicLNMet1
LSVbaseCA
RSVbaseCA
LApexCA
RApexCA
LMidBasalCA
RMidBasalCA
LMidApicalCA
RMidApicalCA
ProstateBPH
ProstateNL
T
Ta
Tb
A
B
C
Ca
Cb
Cba
Cbb
Cba1
Cba2
Cbb1
Cbb2
0Mb
10
20
30
40
50
60
70
80
90
100
110
120
130
140
150
160
170
180

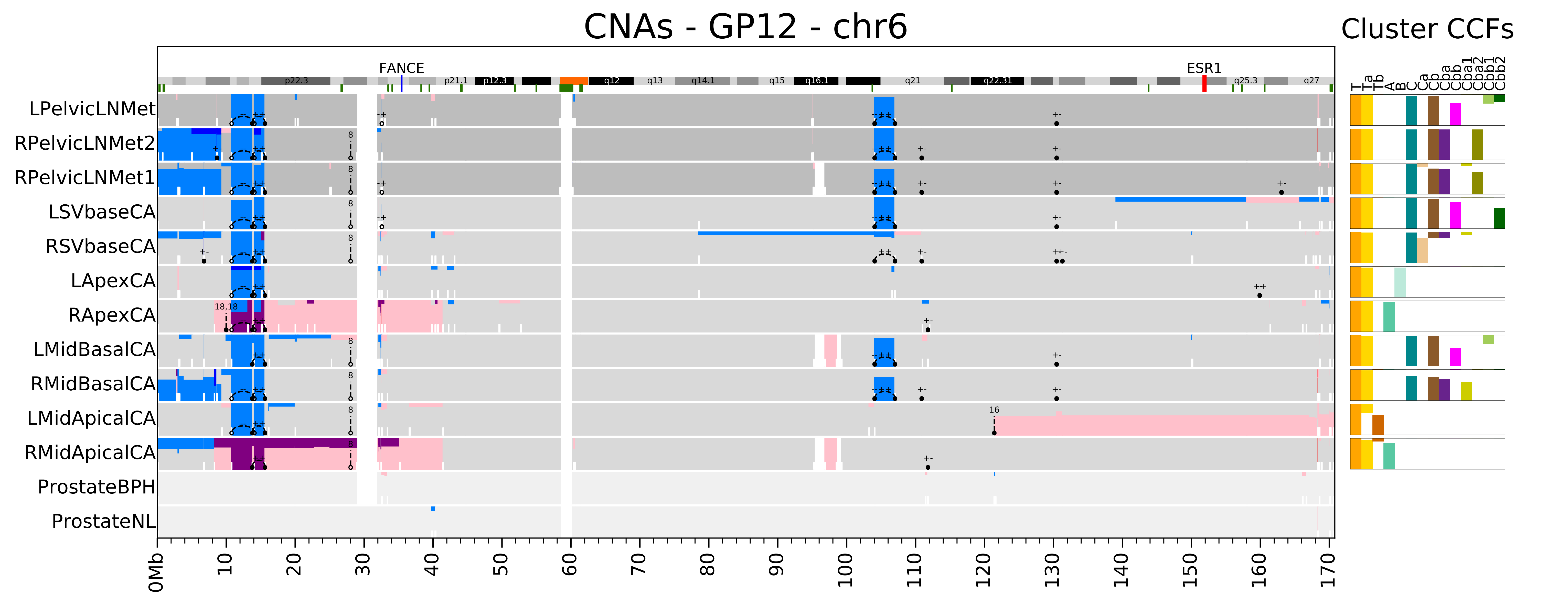

CNAs - GP12 - chr6
Cluster CCFs
FANCE
ESR1
LPelvicLNMet
RPelvicLNMet2
RPelvicLNMet1
LSVbaseCA
RSVbaseCA
LApexCA
RApexCA
LMidBasalCA
RMidBasalCA
LMidApicalCA
RMidApicalCA
ProstateBPH
ProstateNL
T
Ta
Tb
A
B
C
Ca
Cb
Cba
Cbb
Cba1
Cba2
Cbb1
Cbb2
0Mb
10
20
30
40
50
60
70
80
90
100
110
120
130
140
150
160
170

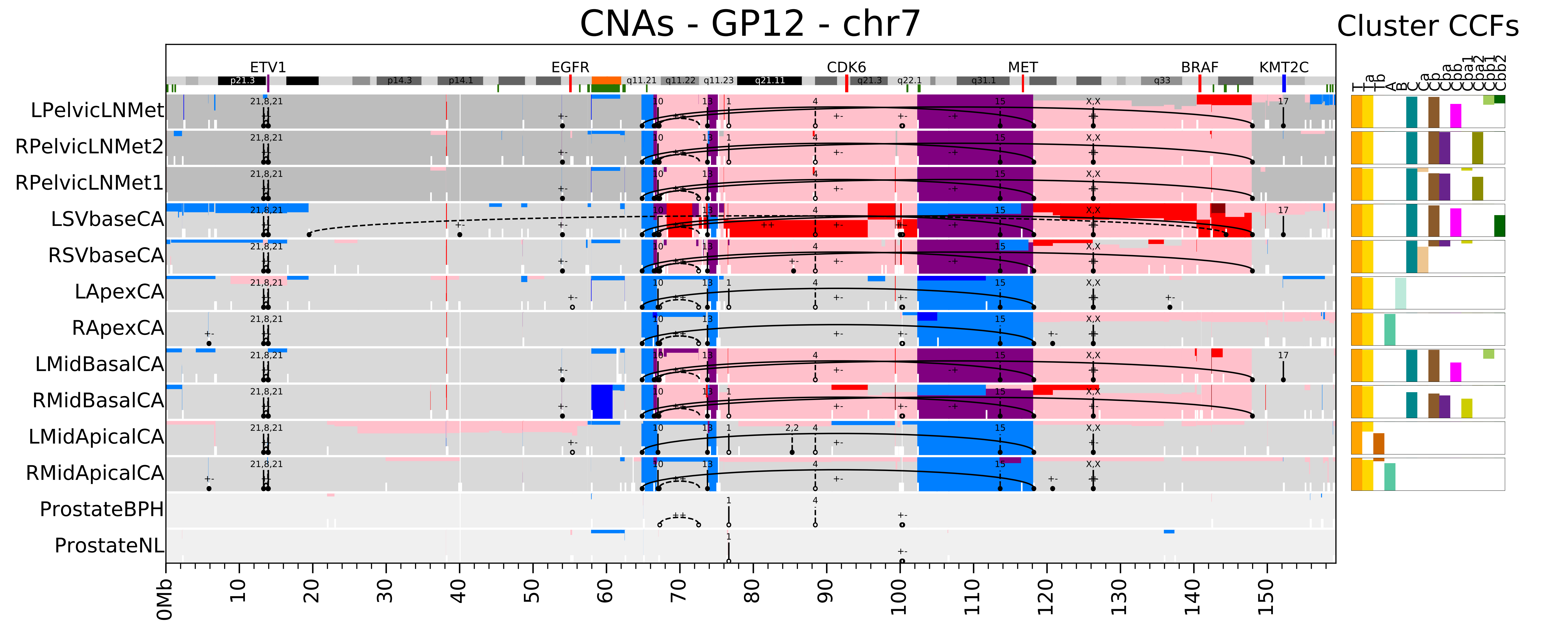

CNAs - GP12 - chr7
Cluster CCFs
ETV1
EGFR
CDK6
MET
BRAF
KMT2C
LPelvicLNMet
RPelvicLNMet2
RPelvicLNMet1
LSVbaseCA
RSVbaseCA
LApexCA
RApexCA
LMidBasalCA
RMidBasalCA
LMidApicalCA
RMidApicalCA
ProstateBPH
ProstateNL
0Mb
10
20
30
40
50
60
70
80
90
100
110
120
130
140
150
T
Ta
Tb
A
B
C
Ca
Cb
Cba
Cbb
Cba1
Cba2
Cbb1
Cbb2

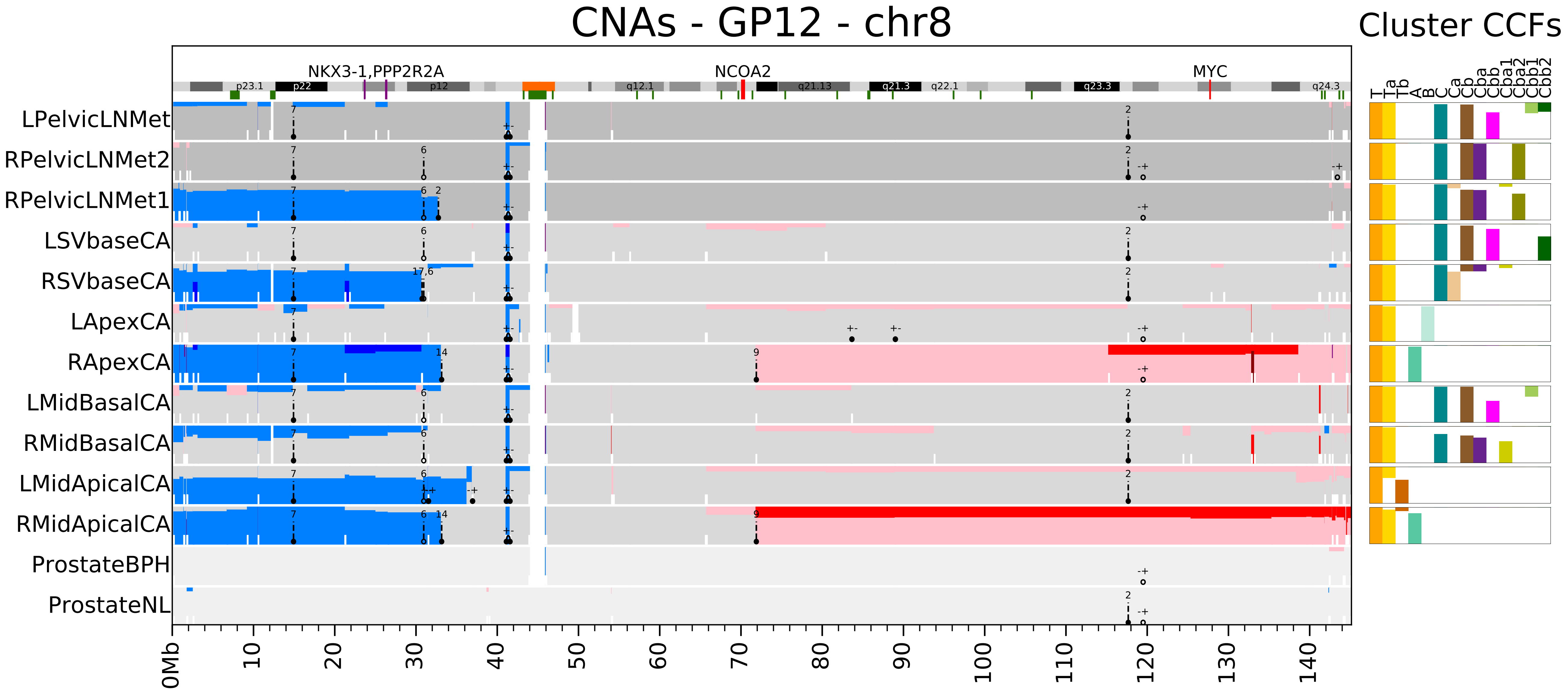

CNAs - GP12 - chr8
Cluster CCFs
NKX3-1,PPP2R2A
NCOA2
MYC
p23.1
p22
p12
q12.1
q21.13
q21.3
q22.1
q23.3
q24.3
LPelvicLNMet
RPelvicLNMet2
RPelvicLNMet1
LSVbaseCA
RSVbaseCA
LApexCA
RApexCA
LMidBasalCA
RMidBasalCA
LMidApicalCA
RMidApicalCA
ProstateBPH
ProstateNL
T
Ta
Tb
A
B
C
Ca
Cb
Cba
Cbb
Cba1
Cba2
Cbb1
Cbb2
0Mb
10
20
30
40
50
60
70
80
90
100
110
120
130
140

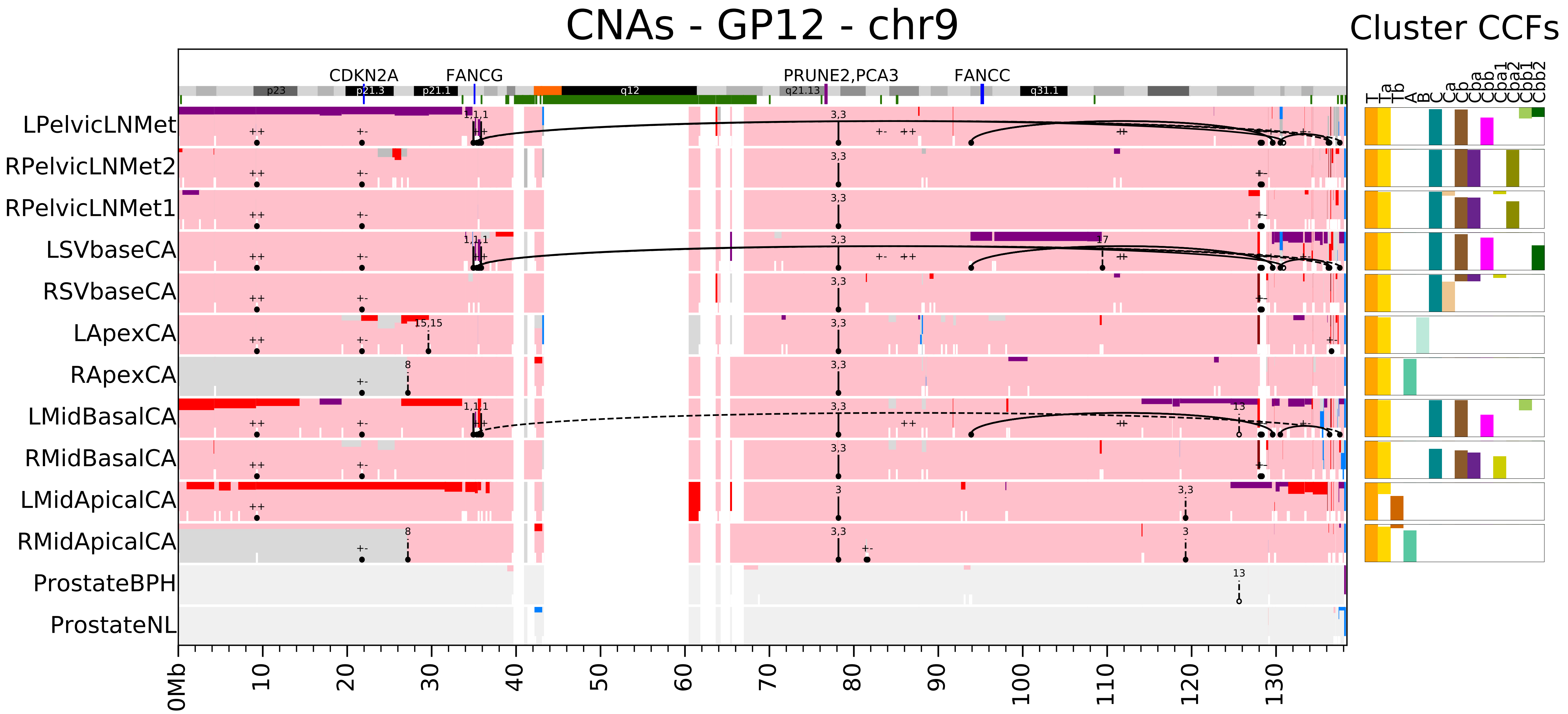

CNAs - GP12 - chr9
Cluster CCFs
CDKN2A
FANCG
PRUNE2,PCA3
FANCC
p23
p21.3
p21.1
q12
q21.13
q31.1
LPelvicLNMet
RPelvicLNMet2
RPelvicLNMet1
LSVbaseCA
RSVbaseCA
LApexCA
RApexCA
LMidBasalCA
RMidBasalCA
LMidApicalCA
RMidApicalCA
ProstateBPH
ProstateNL
0Mb
10
20
30
40
50
60
70
80
90
100
110
120
130
T
Ta
Tb
A
B
C
Ca
Cb
Cba
Cbb
Cba1
Cba2
Cbb1
Cbb2

CNAs - GP12 - chr10

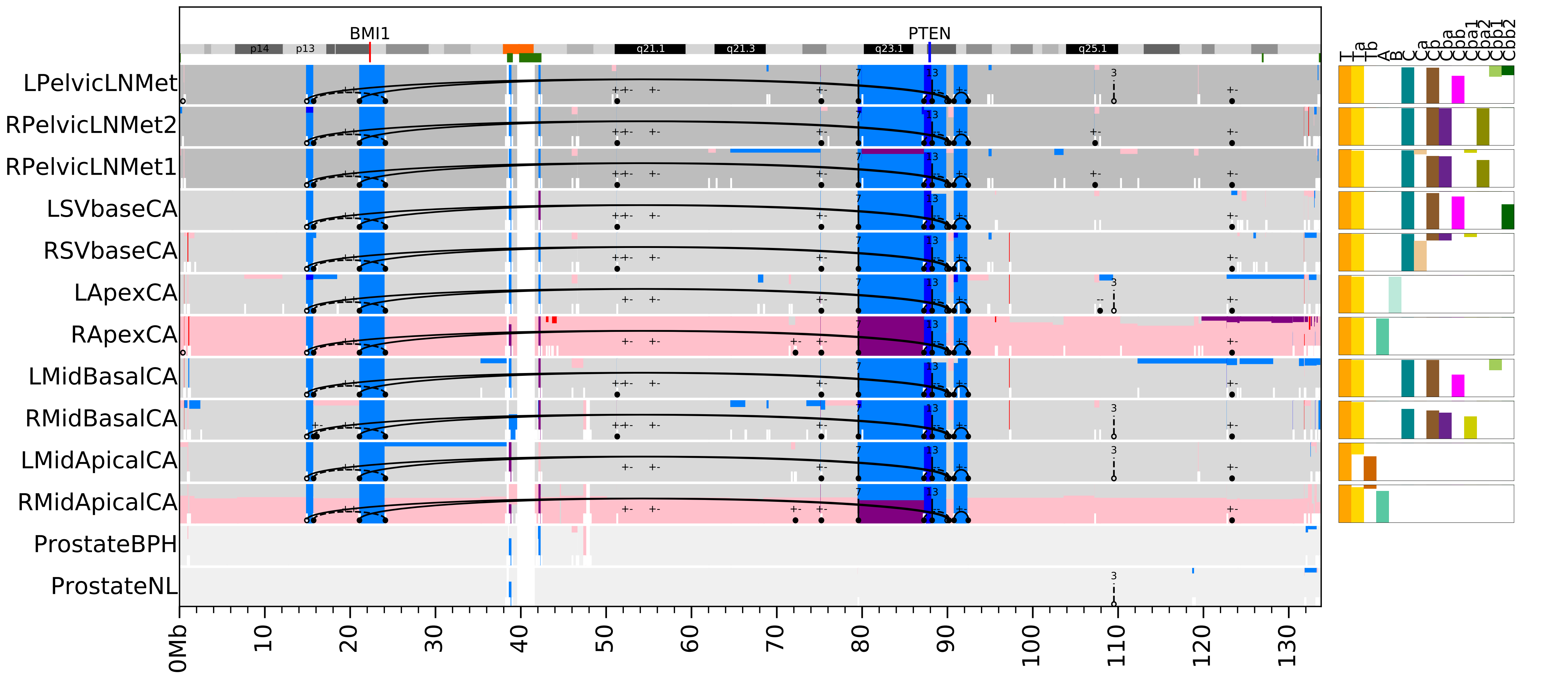

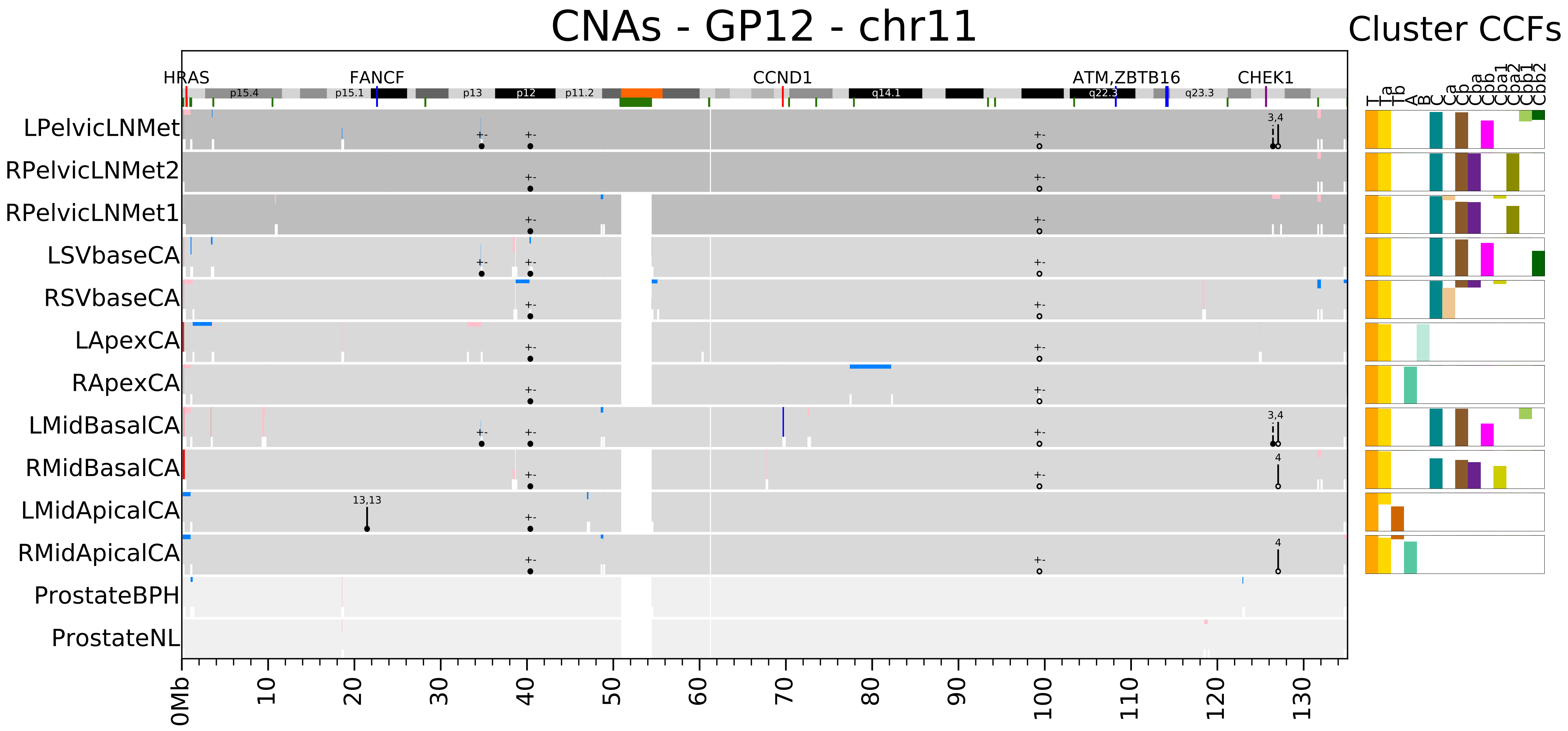

CNAs - GP12 - chr11
Cluster CCFs
HRAS
FANCF
CCND1
ATM,ZBTB16
CHEK1
p15.4
p15.1
p13
p12
p11.2
q14.1
q22.3
q23.3
LPelvicLNMet
RPelvicLNMet2
RPelvicLNMet1
LSVbaseCA
RSVbaseCA
LApexCA
RApexCA
LMidBasalCA
RMidBasalCA
LMidApicalCA
RMidApicalCA
ProstateBPH
ProstateNL
3,4
13,13
4
0Mb
10
20
30
40
50
60
70
80
90
100
110
120
130
T
Ta
Tb
A
B
C
Ca
Cb
Cba
Cbb
Cba1
Cba2
Cbb1
Cbb2

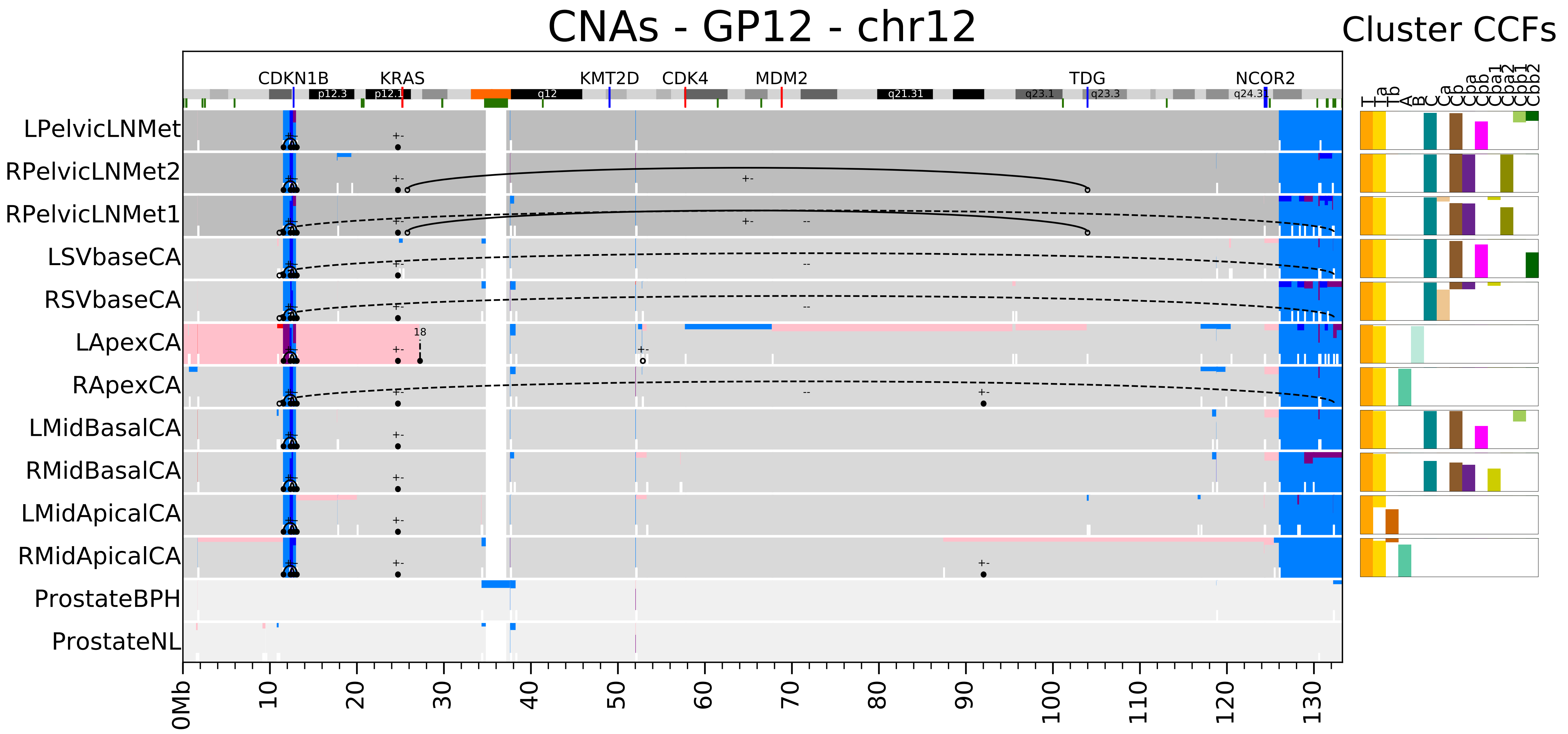

CNAs - GP12 - chr12
Cluster CCFs
CDKN1B
KRAS
KMT2D
CDK4
MDM2
TDG
NCOR2
p12.3
p12.1
q12
q21.31
q23.1
q23.3
q24.31
T
Ta
Tb
A
B
C
Ca
Cb
Cba
Cbb
Cba1
Cba2
Cbb1
Cbb2
LPelvicLNMet
RPelvicLNMet2
RPelvicLNMet1
LSVbaseCA
RSVbaseCA
LApexCA
RApexCA
LMidBasalCA
RMidBasalCA
LMidApicalCA
RMidApicalCA
ProstateBPH
ProstateNL
18
0Mb
10
20
30
40
50
60
70
80
90
100
110
120
130

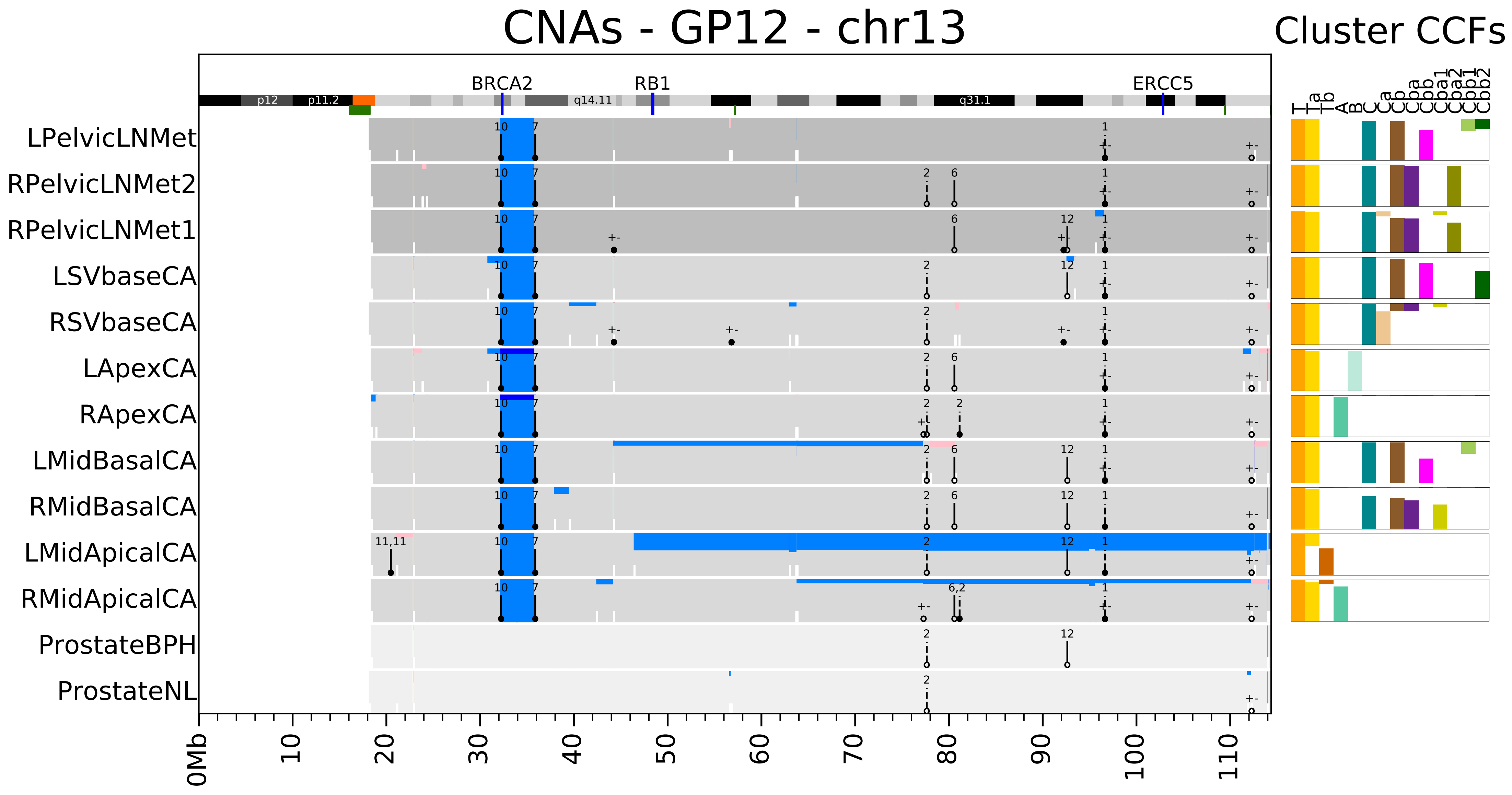

CNAs - GP12 - chr13
Cluster CCFs
BRCA2
RB1
ERCC5
p12
p11.2
q14.11
q31.1
LPelvicLNMet
RPelvicLNMet2
RPelvicLNMet1
LSVbaseCA
RSVbaseCA
LApexCA
RApexCA
LMidBasalCA
RMidBasalCA
LMidApicalCA
RMidApicalCA
ProstateBPH
ProstateNL
0Mb
10
20
30
40
50
60
70
80
90
100
110
T
Ta
Tb
A
B
C
Ca
Cb
Cba
Cbb
Cba1
Cba2
Cbb1
Cbb2

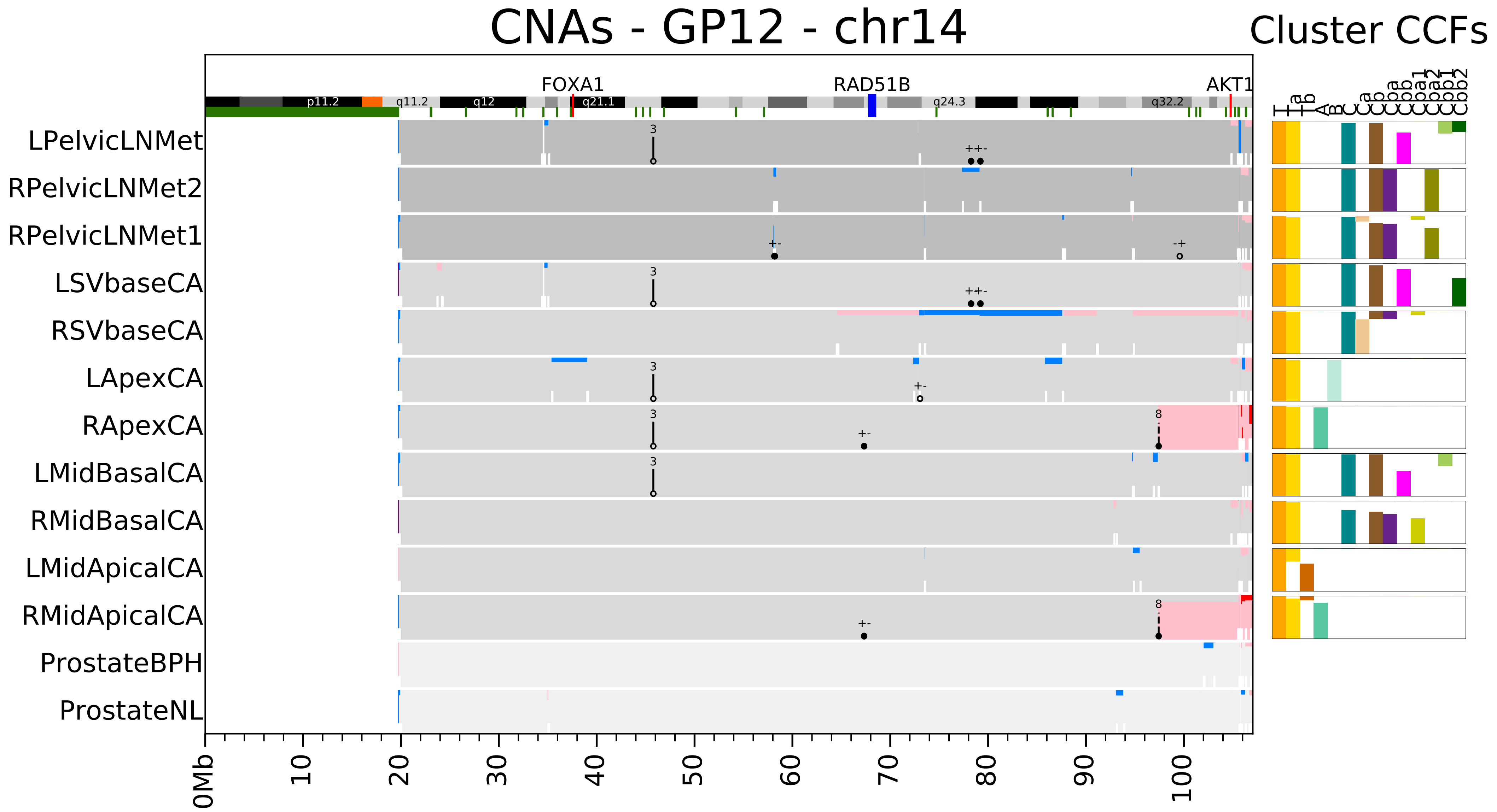

CNAs - GP12 - chr14
Cluster CCFs
FOXA1
RAD51B
AKT1
p11.2
q11.2
q12
q21.1
q24.3
q32.2
LPelvicLNMet
RPelvicLNMet2
RPelvicLNMet1
LSVbaseCA
RSVbaseCA
LApexCA
RApexCA
LMidBasalCA
RMidBasalCA
LMidApicalCA
RMidApicalCA
ProstateBPH
ProstateNL
0Mb
10
20
30
40
50
60
70
80
90
100
T
Ta
Tb
A
B
C
Ca
Cb
Cba
Cbb
Cba1
Cba2
Cbb1
Cbb2

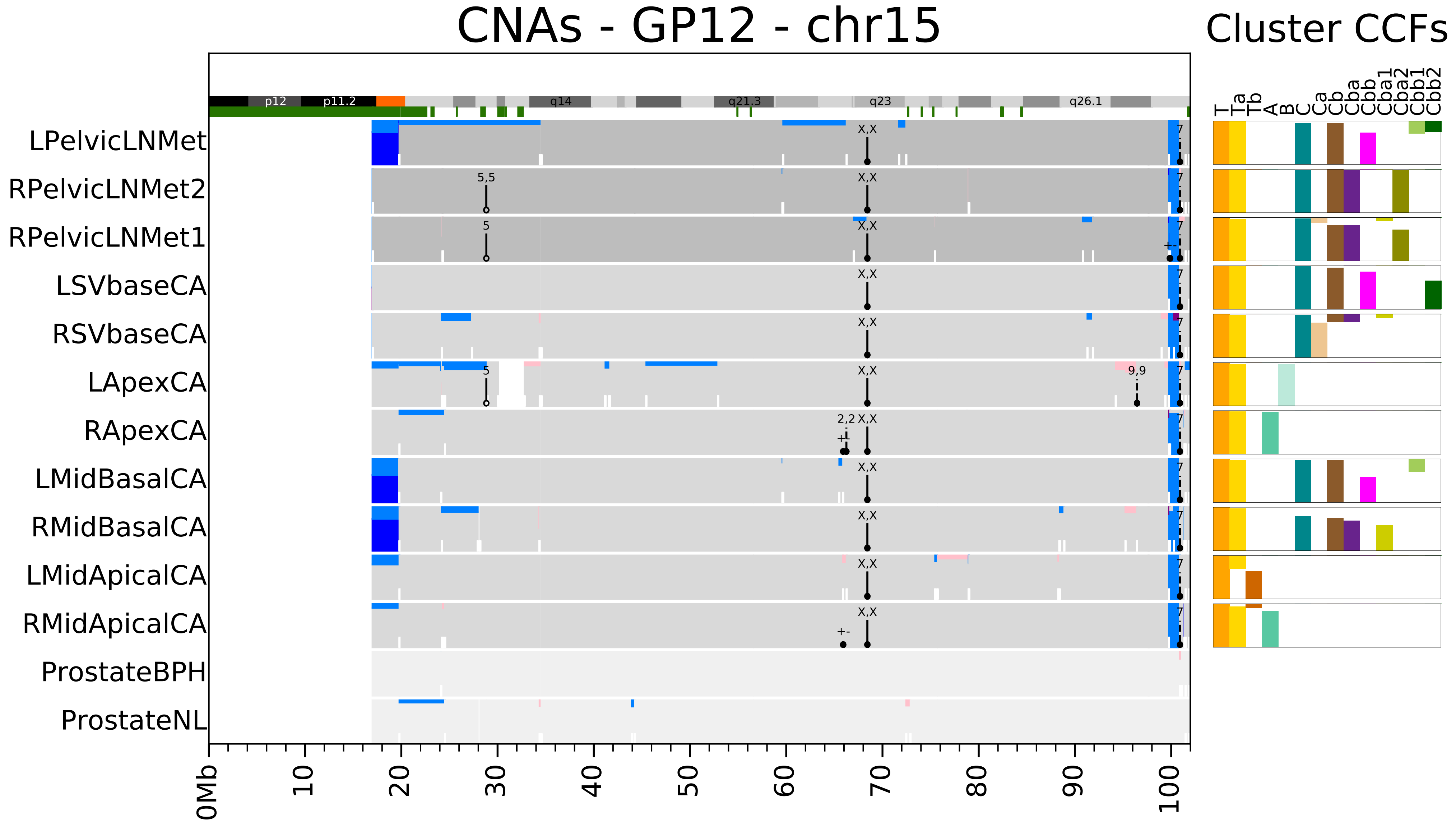

CNAs - GP12 - chr15
Cluster CCFs
p12
p11.2
q14
q21.3
q23
q26.1
LPelvicLNMet
RPelvicLNMet2
RPelvicLNMet1
LSVbaseCA
RSVbaseCA
LApexCA
RApexCA
LMidBasalCA
RMidBasalCA
LMidApicalCA
RMidApicalCA
ProstateBPH
ProstateNL
X,X
5,5
5
9,9
2,2
7
+-
0Mb
10
20
30
40
50
60
70
80
90
100
T
Ta
Tb
A
B
C
Ca
Cb
Cba
Cbb
Cba1
Cba2
Cbb1
Cbb2

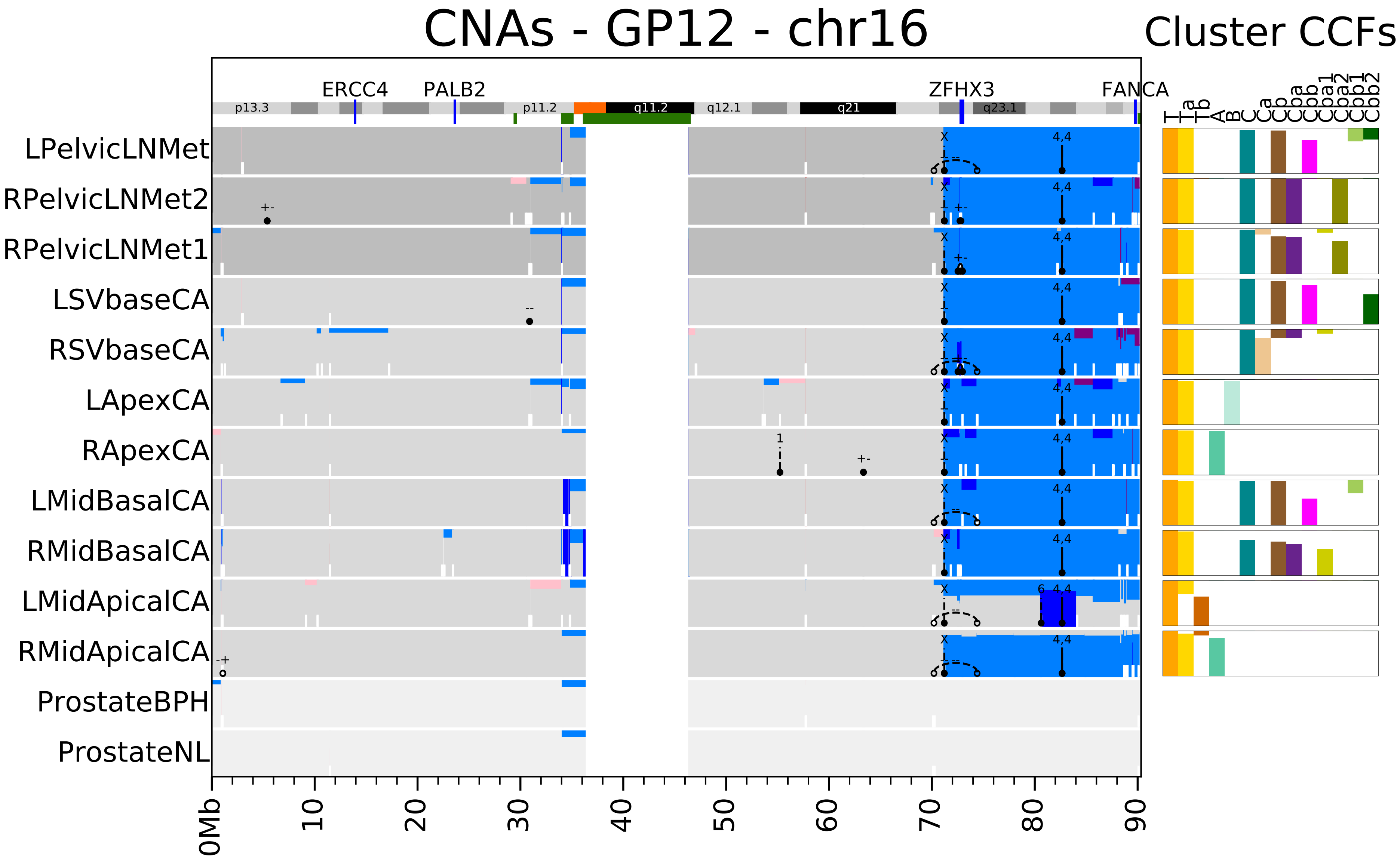

CNAs - GP12 - chr16
Cluster CCFs
ERCC4
PALB2
ZFHX3
FANCA
p13.3
p11.2
q11.2
q12.1
q21
q23.1
LPelvicLNMet
RPelvicLNMet2
RPelvicLNMet1
LSVbaseCA
RSVbaseCA
LApexCA
RApexCA
LMidBasalCA
RMidBasalCA
LMidApicalCA
RMidApicalCA
ProstateBPH
ProstateNL
T
Ta
Tb
A
B
C
Ca
Cb
Cba
Cbb
Cba1
Cba2
Cbb1
Cbb2
0Mb
10
20
30
40
50
60
70
80
90

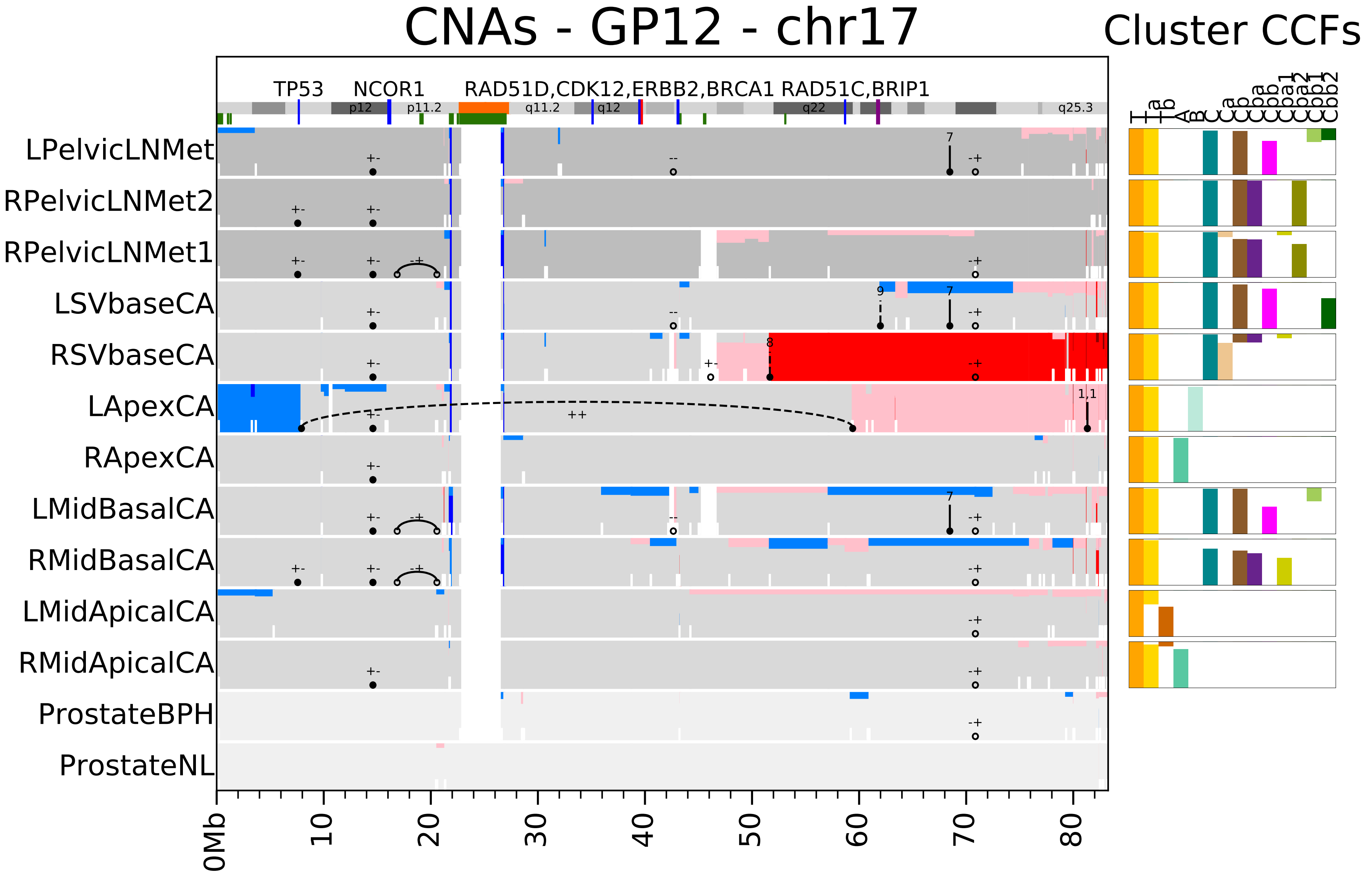

CNAs - GP12 - chr17
Cluster CCFs
TP53
NCOR1
RAD51D,CDK12,ERBB2,BRCA1
RAD51C,BRIP1
p12
p11.2
q11.2
q12
q22
q25.3
LPelvicLNMet
RPelvicLNMet2
RPelvicLNMet1
LSVbaseCA
RSVbaseCA
LApexCA
RApexCA
LMidBasalCA
RMidBasalCA
LMidApicalCA
RMidApicalCA
ProstateBPH
ProstateNL
0Mb
10
20
30
40
50
60
70
80
T
Ta
Tb
A
B
C
Ca
Cb
Cba
Cbb
Cba1
Cba2
Cbb1
Cbb2

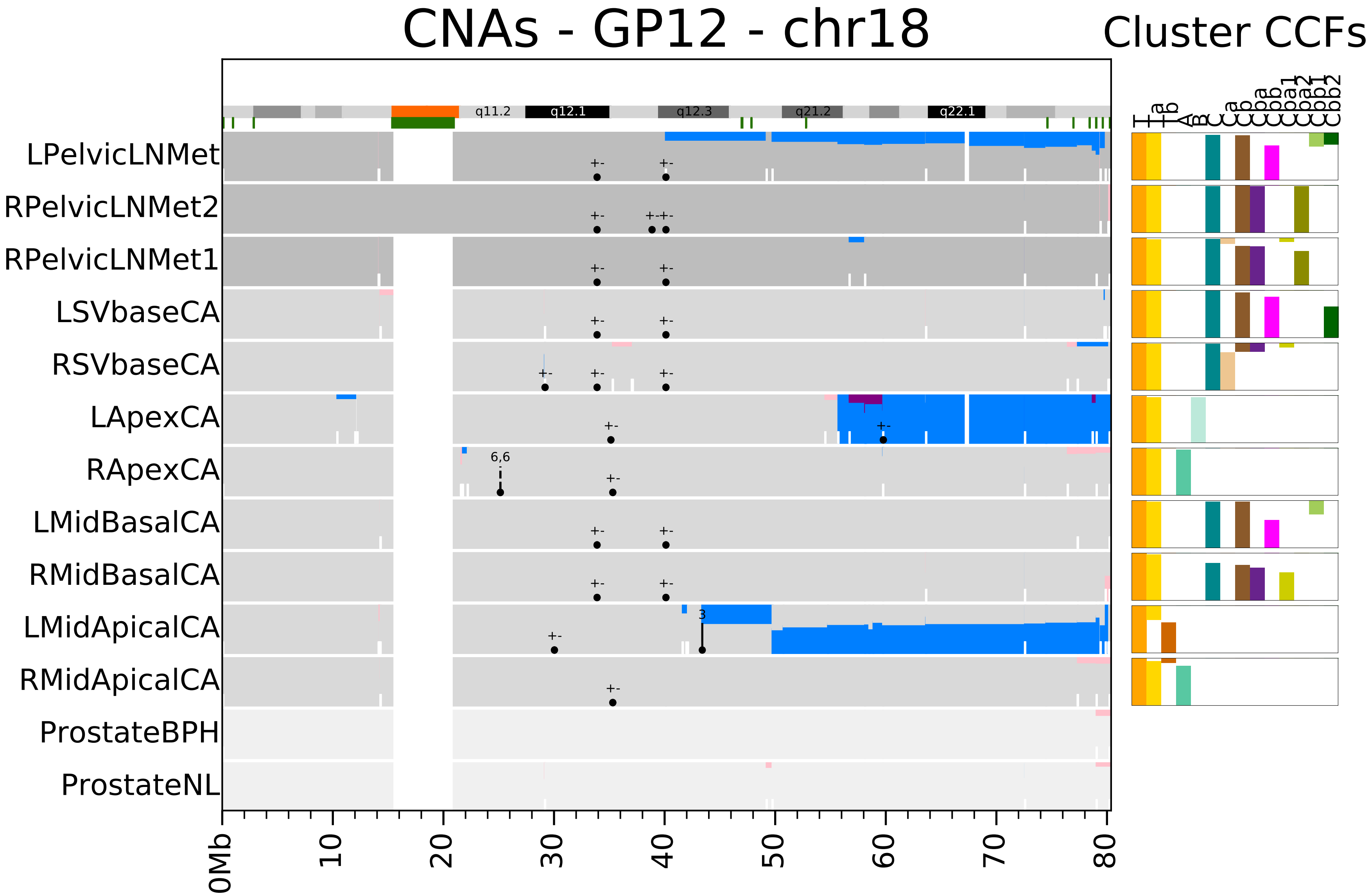

CNAs - GP12 - chr18
Cluster CCFs
q11.2
q12.1
q12.3
q21.2
q22.1
LPelvicLNMet
RPelvicLNMet2
RPelvicLNMet1
LSVbaseCA
RSVbaseCA
LApexCA
RApexCA
LMidBasalCA
RMidBasalCA
LMidApicalCA
RMidApicalCA
ProstateBPH
ProstateNL
6,6
3
0Mb
10
20
30
40
50
60
70
80
T
Ta
Tb
A
B
C
Ca
Cb
Cba
Cbb
Cba1
Cba2
Cbb1
Cbb2

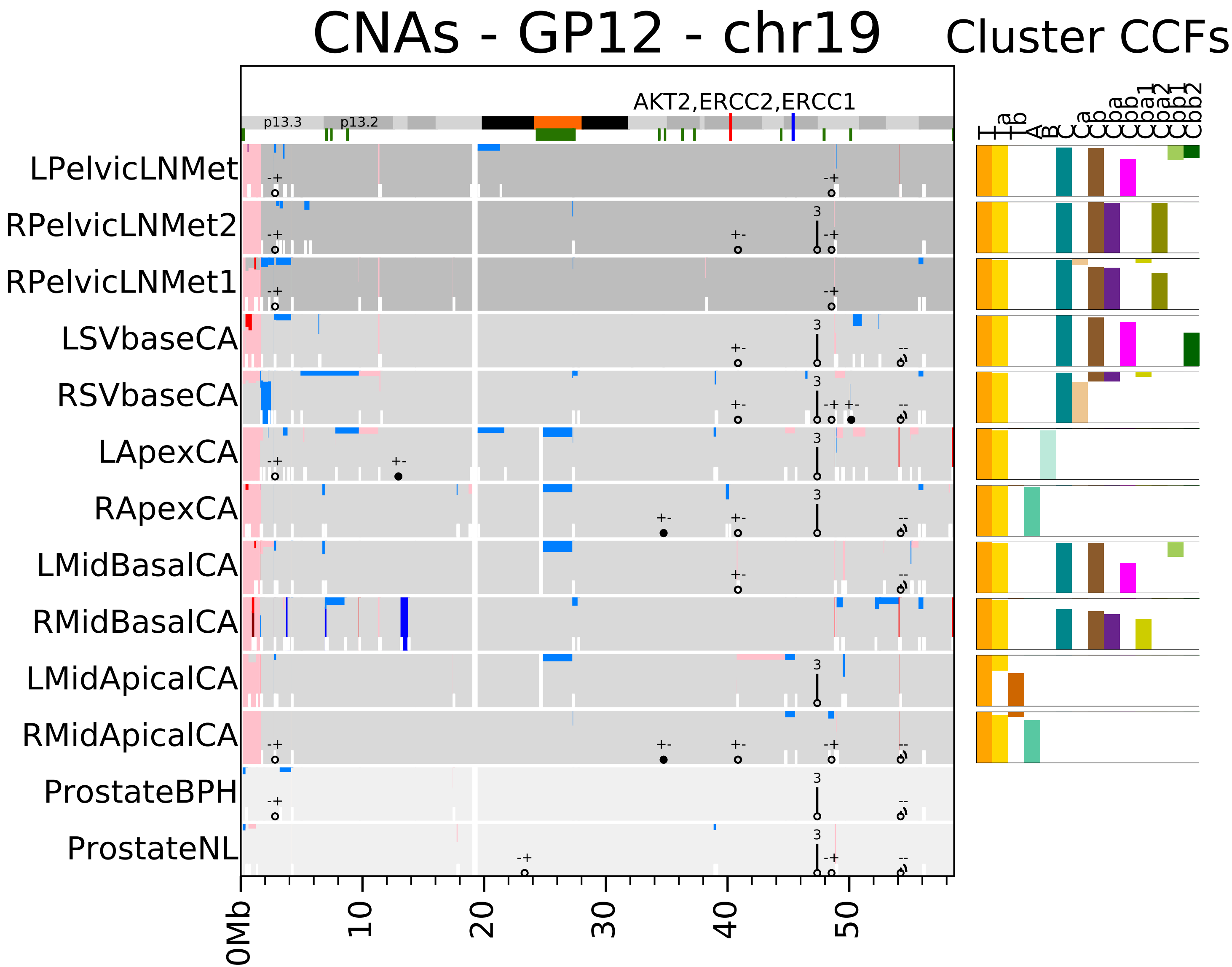
CNAs - GP12 - chr19
Cluster CCFs
AKT2,ERCC2,ERCC1
p13.3
p13.2
LPelvicLNMet
RPelvicLNMet2
RPelvicLNMet1
LSVbaseCA
RSVbaseCA
LApexCA
RApexCA
LMidBasalCA
RMidBasalCA
LMidApicalCA
RMidApicalCA
ProstateBPH
ProstateNL
0Mb
10
20
30
40
50
T
Ta
Tb
A
B
C
Ca
Cb
Cba
Cbb
Cba1
Cba2
Cbb1
Cbb2

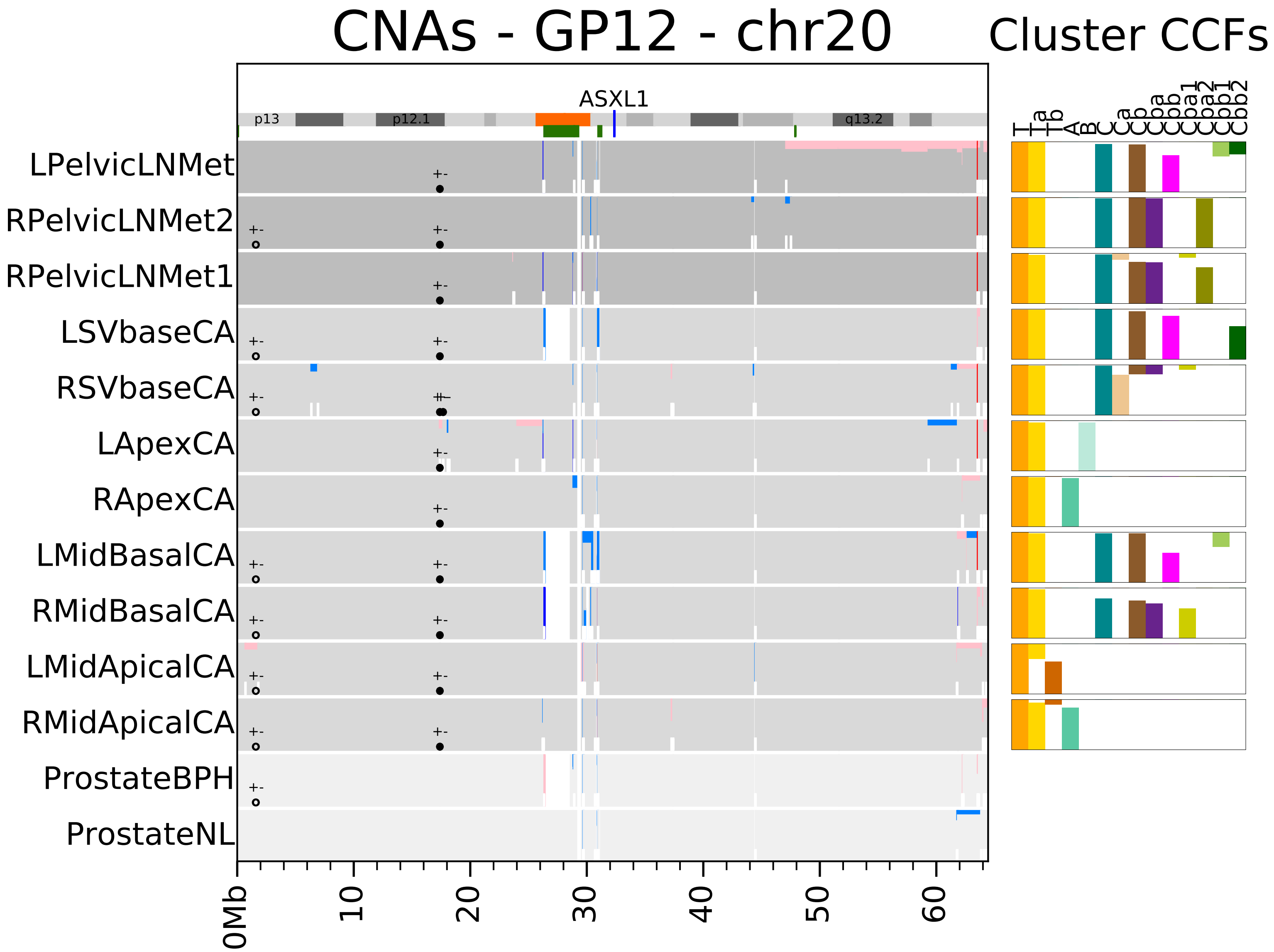

CNAs - GP12 - chr20
Cluster CCFs
ASXL1
p13
p12.1
q13.2
LPelvicLNMet
RPelvicLNMet2
RPelvicLNMet1
LSVbaseCA
RSVbaseCA
LApexCA
RApexCA
LMidBasalCA
RMidBasalCA
LMidApicalCA
RMidApicalCA
ProstateBPH
ProstateNL
0Mb
10
20
30
40
50
60
T
Ta
Tb
A
B
C
Ca
Cb
Cba
Cbb
Cba1
Cba2
Cbb1
Cbb2

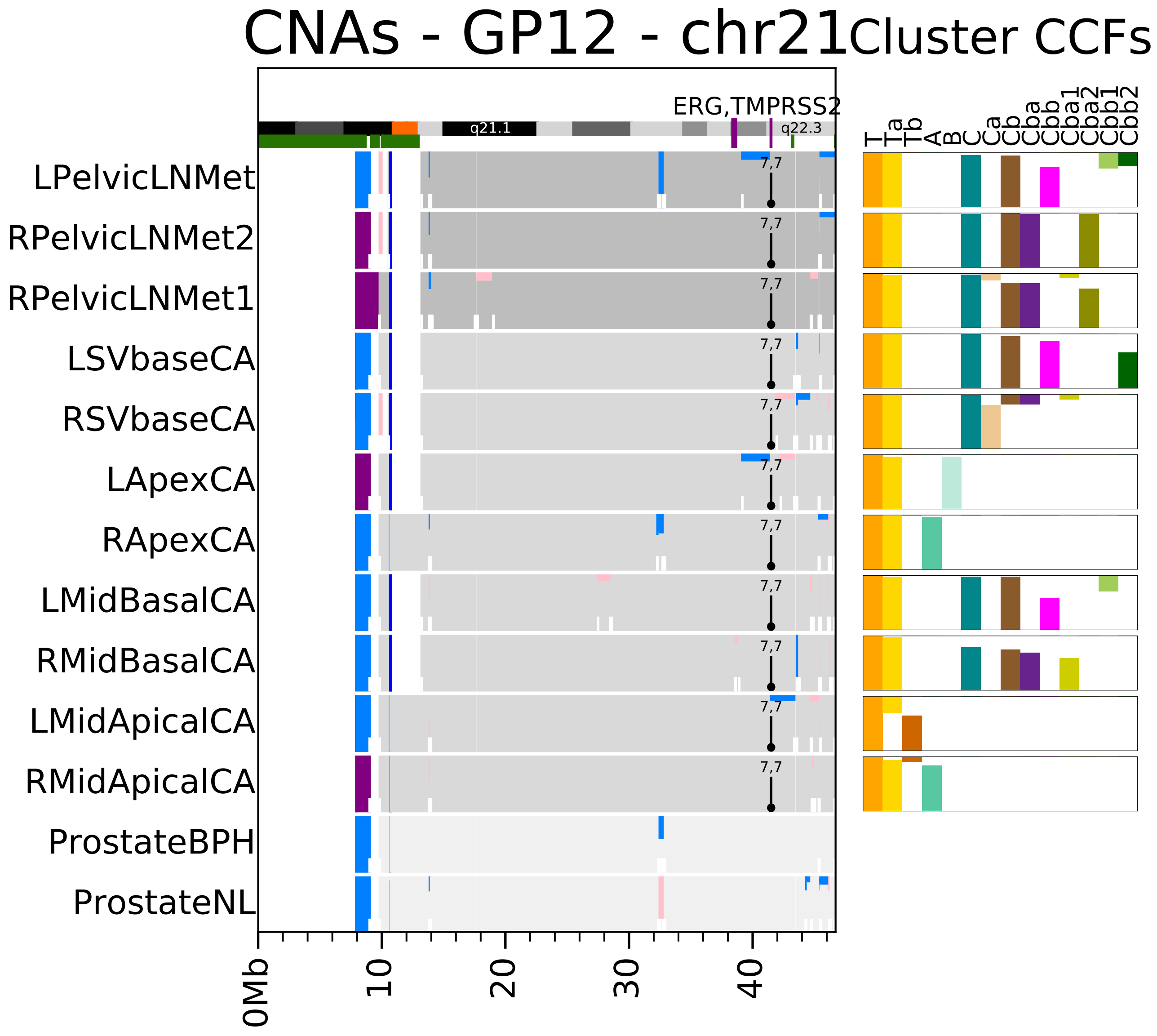

CNAs - GP12 - chr21
Cluster CCFs
ERG,TMPRSS2
q21.1
q22.3
LPelvicLNMet
RPelvicLNMet2
RPelvicLNMet1
LSVbaseCA
RSVbaseCA
LApexCA
RApexCA
LMidBasalCA
RMidBasalCA
LMidApicalCA
RMidApicalCA
ProstateBPH
ProstateNL
7,7
0Mb
10
20
30
40
T
Ta
Tb
A
B
C
Ca
Cb
Cba
Cbb
Cba1
Cba2
Cbb1
Cbb2

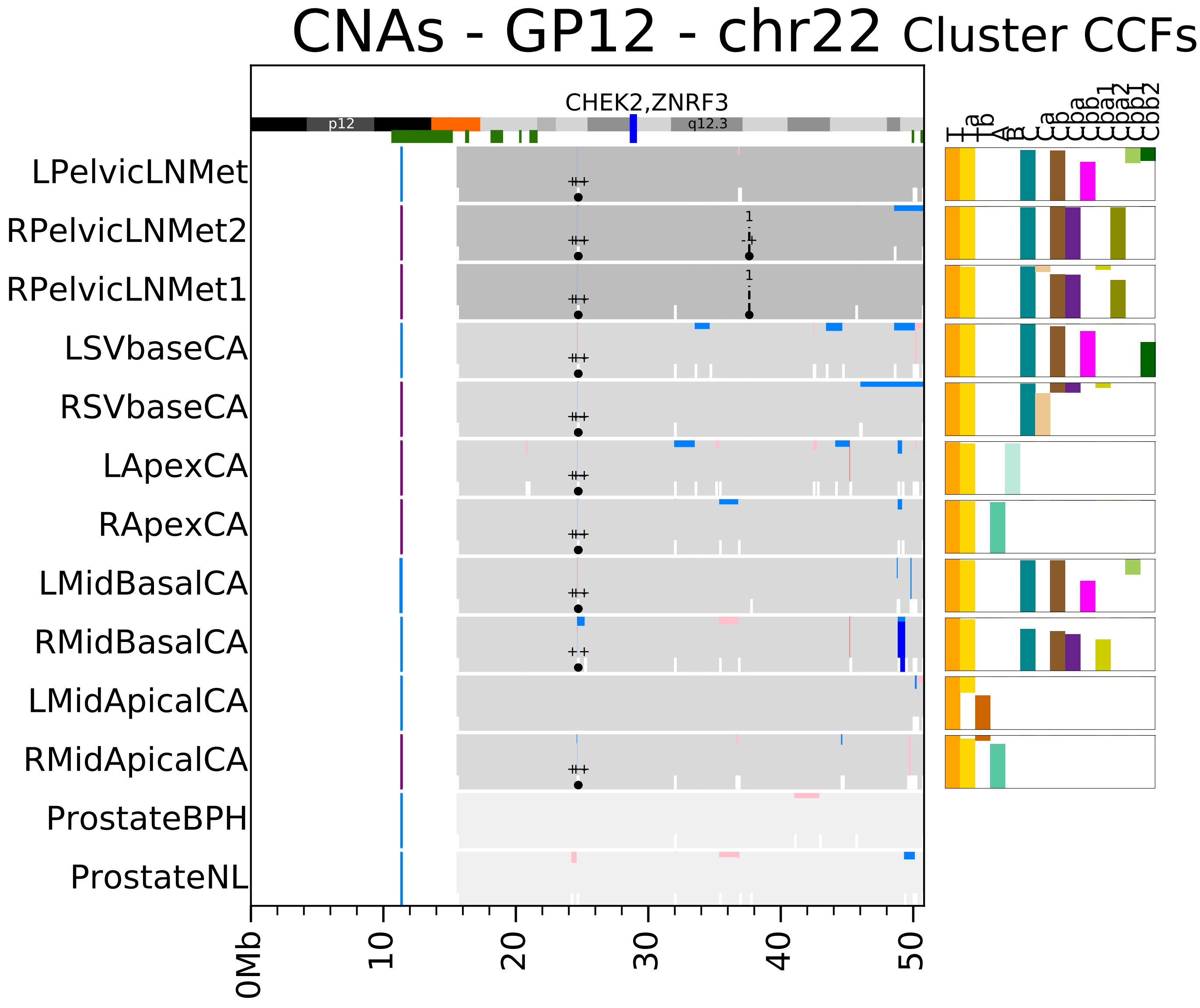
CNAs - GP12 - chr22
Cluster CCFs
CHEK2,ZNRF3
p12
q12.3
LPelvicLNMet
RPelvicLNMet2
RPelvicLNMet1
LSVbaseCA
RSVbaseCA
LApexCA
RApexCA
LMidBasalCA
RMidBasalCA
LMidApicalCA
RMidApicalCA
ProstateBPH
ProstateNL
0Mb
10
20
30
40
50
I
Ia
Ib
A
B
C
Ca
Cb
Cba
Cbb
Cba1
Cba2
Cbb1
Cbb2

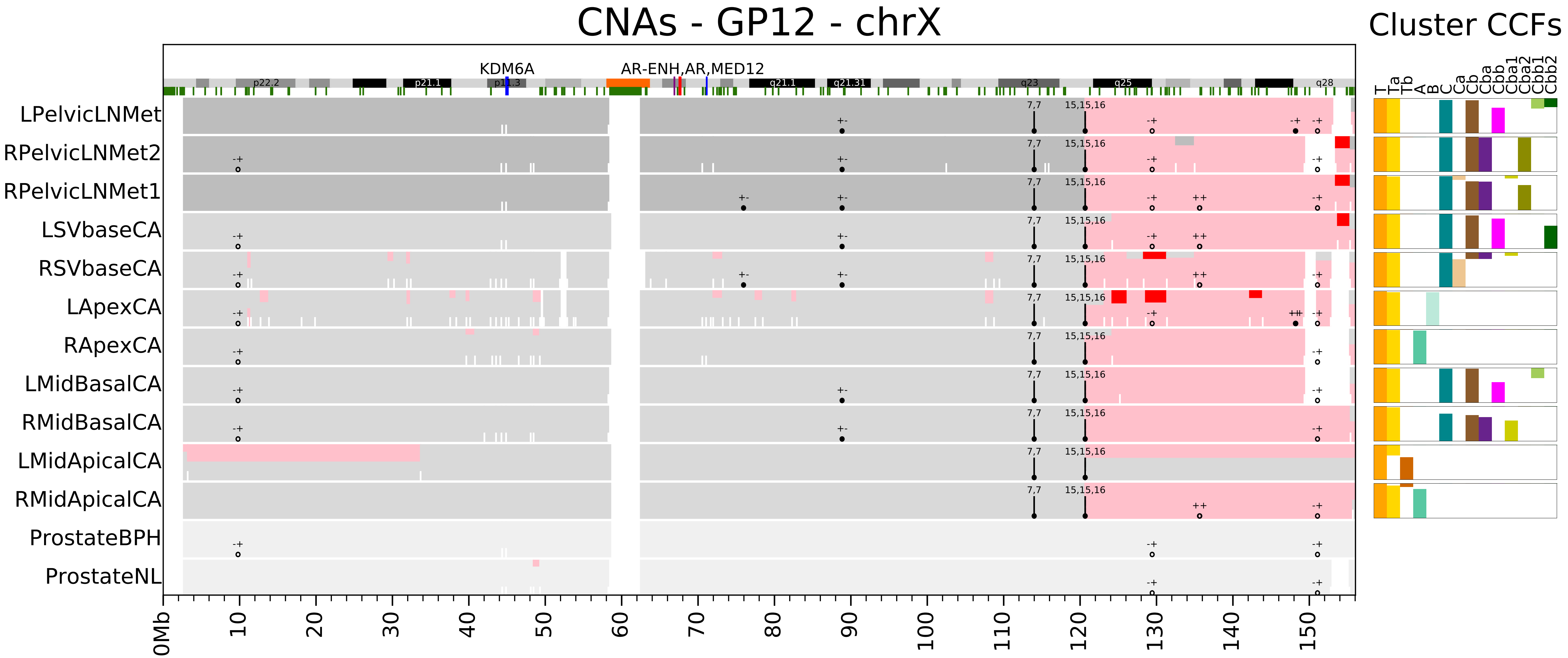

CNAs - GP12 - chrX
Cluster CCFs
KDM6A
AR-ENH,AR,MED12
p22.2
p21.1
p11.3
q21.1
q21.31
q23
q25
q28
LPelvicLNMet
RPelvicLNMet2
RPelvicLNMet1
LSVbaseCA
RSVbaseCA
LApexCA
RApexCA
LMidBasalCA
RMidBasalCA
LMidApicalCA
RMidApicalCA
ProstateBPH
ProstateNL
7,7
15,15,16
0Mb
10
20
30
40
50
60
70
80
90
100
110
120
130
140
150
T
Ta
Tb
A
B
C
Ca
Cb
Cba
Cbb
Cba1
Cba2
Cbb1
Cbb2

Fig. S9 GP12 Somatic Tumor DNA Alteration Whole Chromosome Linear Plots. CNAs and structural variants detected in each sample are visualized in their separate horizontal lanes. The fraction of coloring used for the CNAs inside the lanes is proportional to the fraction of cancer cells having the copy number alteration. Fractions below 50% are displayed at the top of the lane while higher fractions are displayed from the bottom up. Prostate cancer -associated genes are shown at the top of the lanes at their genomic coordinates. Centromeres are displayed in orange on the cytoband information graphic. Below the cytoband, green coloring is used for “blacklisted” genomic regions(6) where CNA and SV calls may have low confidence. Evolutionary clusters CCFs are drawn in a separate table on the right side of each chromosome with colored bars proportional to lane height in each sample, so that a colored bar filling the whole lane equals 100% CCF and no bar meaning that the cluster is not detected in the sample. Black dots that do not have a connecting arc represent intrachromosomal breakpoints that are less than 100Kb apart. Tandem duplications (dots connected with solid arcs) are drawn using the color of the cluster they have been assigned to.

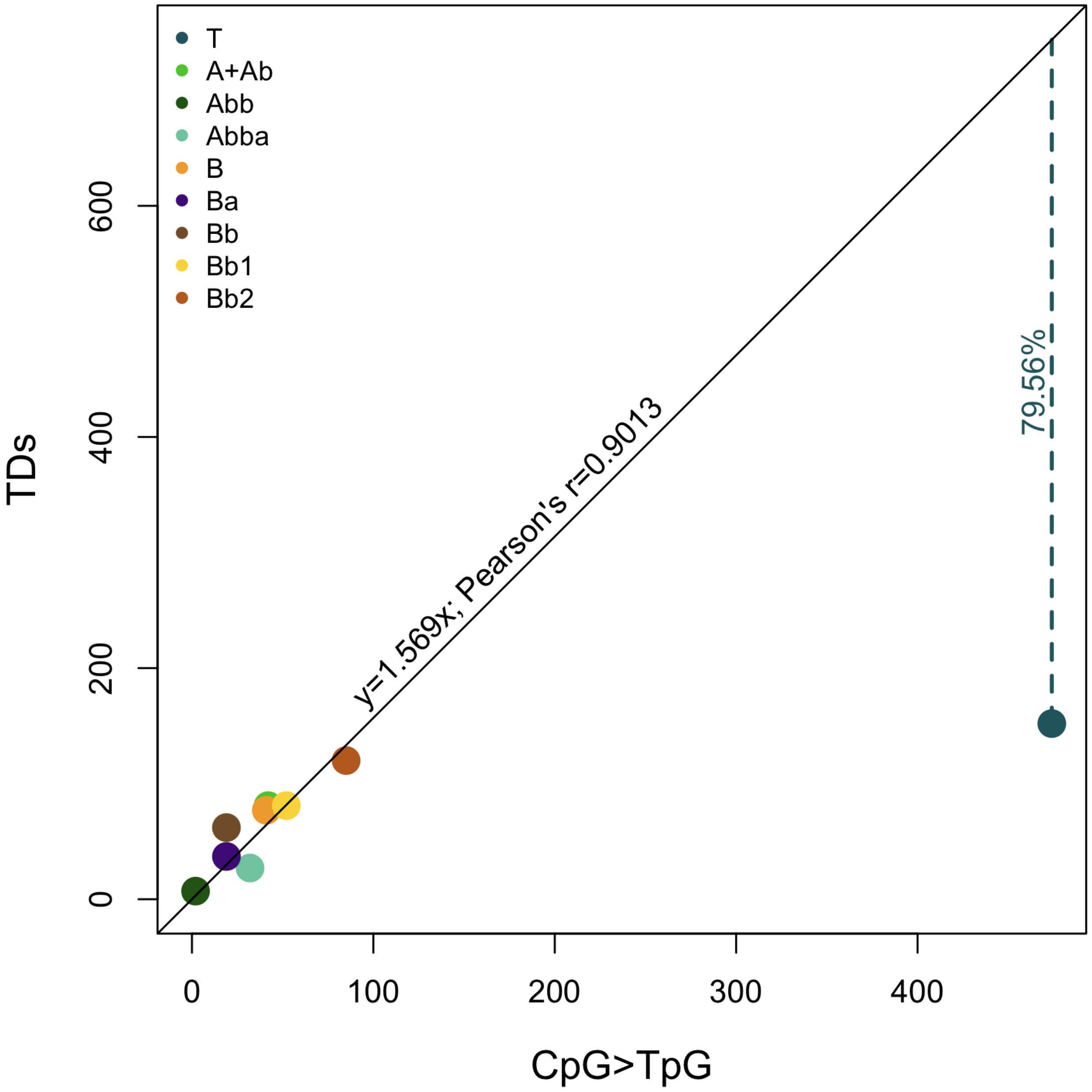

Fig. S10 Correlation of Tandem Duplications (TDs) and CpG>TpG (SBS1) nucleotide mutation per GP5 cancer subclone. Apart from subclone T, we measured a strong linear relationship (Pearson's r = 0.9013), suggesting that TDs occurred at a reasonably constant rate relative to subclonal evolution. The offset of subclone T gives an estimate of when the TD phenotype started as it corresponds to 79.564% of subclone T molecular time (Fig. 7). To obtain robust TD estimates, we only considered subclones with high CCF values and distinguishable TDs, thereby excluding subclones Baa, Bc, Aba and Aa from this analysis. Because TDs in clusters Ab and A can not be distinguished with high confidence based on CNAs fractions alone (similar CCFs, see Fig. 3a), clusters A and Ab were merged for the analysis.

SUPPLEMENTARY METHODS

Cancer volume mapping, laser dissection, and DNA isolation and qualification

This protocol uses both the PAXgene Tissue DNA Kit (Qiagen/PreAnalytix cat no 767134) for the isolation of DNA. During overnight processing of the GP5 tissue samples following the previously published protocol (7), the dedicated PAXgene tissue processor jammed with tissues submerged in 90% ethanol (but after PAXgene fixation) for 12 hours. Despite this, gDNA quality among GP5 and GP12 samples was similar. Fragment Analyzer (Agilent) mean (range) DNA GQN (Genomic DNA Quality Number) was 5.1 (3.9-7), and 5.1 (3.7-6.8) for DNA from LCM dissected samples from GP5 and GP12 respectively.

DNA sequencing, alignment, and variant calls

Whole genome DNA sequencing (PE150, 150 bp paired-end reads) was performed to a median read depth of 58X for the blood normal samples, and 71X for primary tumor, tissue normal, and metastatic samples using Novaseq 6000 machines with S4 reagents (Novogene). Whole genome sequence reads were aligned to human genome reference GRCh38 GCA_000001405.15 using the following tools: BWA-MEM 0.7.17 for alignment, samblaster 0.1.24 for marking duplicate reads, samtools 1.8 for sorting and picard 2.21.8 for assigning the reads to read-groups. Software and reference data sets used in the project are listed in Table S1. The aligned paired-end reads were processed for variant calling according to GATK somatic short variant discovery best-practices guidelines, by running GATK 4.1.8.1 with Base Quality Score Recalibration (BQSR) (BaseRecalibrator and ApplyBQSR). The following resource files from the GATK Resource Bundle were used for the BaseRecalibrator “known-sites” argument: 1000G_phase1_snps, Mills_and_1000G_gold_standard_indels and Homo_sapiens_assembly38_dbsnp138(1).

Germline DNA of both patients was analyzed with GATK/Haplotypecaller (v4.1.3.0) and annotated with Annovar (version 2019Oct24). No PrCa-predisposing genetic variants were identified. Somatic short variants (SSVs: SNVs and indels) were detected with GATK Mutect2 in matched normal mode, using genomic DNA isolated from leukocytes (blood) from each patient as a reference normal. Mutect2 was run with GATK Best Practices Bundle AF-only gnomAD resource file (“germline-resource” argument), containing population allele frequencies of common and rare variants. Mutect2 was run with a panel of normals (PoN) consisting of 99 non-cancerous tissue and blood WGS samples. The PoN consisted of 20 samples from the PELICAN autopsy studies (11 blood, 5 spleen, 3 liver and 1 kidney samples), 6 samples from the Geoprostate study (4 blood and 2 prostate samples) and WGS data from 73 blood samples that were obtained through the ICGC (8). The PoN sequencing data was aligned and prepared in an identical manner to previously described patient WGS data. The aligned and base quality recalibrated WGS data was further processed according to the “GATK how to create panel of normals best-practices guidelines” by running GATK 4.1.8.1 in “tumor-only” mode, and finally combining the variant calls into a single file using GATK CreateSomaticPanelOfNormals.

The detected SSVs were filtered using GATK LearnReadOrientationModel and FilterMutectCall, followed by left-aligning and trimming the indels with LeftAlignAndTrimVariants. A post-filtering step was applied to each sample to rescue some of the filtered-out variants based on the following rules: 1) the call had ≥1 reads in each direction, to cancel "strand_bias" rejection, or 2) the call had AF above 0.1 with >0 ALT reads, to cancel the "slippage" filter. A further exclusive post-filtering phase was applied to reject calls according to the following rules: 1) call did not have reads in both directions, or 2) the call did not have more than 2 reads in at least one direction, and 3) insertion

and deletion calls that had more than 5 repeated bases, but did not have ≥ 5 reads in both directions.

GATK/Mutect2 has a multisample mode that has recently become available, but was not used in the current study. The multisample mode is designed for comparison of samples taken from the same subject at different time points, rather than multiple samples taken at the same time point as is the case in the current study. In comparison to the pairwise tumor-normal mode, the multisample mode produced large amounts of false positive calls in samples where a mutation was present in only a subset of the samples. By applying our post-filtering steps to a deep sequenced dataset from a previous study (4), we found that 0.7% of calls rejected based on DNA polymerase slippage ("slippage") and biased read strand distributions ("strand_bias") could be accepted to improve accuracy. On the other hand, using the exclusive post-filtering steps allowed us to reject 15.1% of calls accepted by Mutect2. Calls that did not exceed the minimum requirements to pass the exclusive post-filtering criteria in any of the 13 samples per patient were removed from further processing. Analysis of the post-filtering step on the deep sequenced validation dataset showed an overall increase of 0.3% and 0.5% in precision and recall respectively.

The SSVs were filtered further by excluding unreliably mapped regions according to the GEM-mappability tool v1.315, resulting in removal of 1758 / 23910 (7.4%) of SSVs for GP5 and 1628 / 23165 (7.0%) for GP12. As a final filtering step, the SSVs were compared with variant population frequencies obtained from the Genome Aggregation Database (gnomAD v3.1). Excluding all germline variants above 1.0% frequency in any population resulted in removal of further 604 / 22152 (2.7%) SSVs for GP5 and 305 / 21537 (1.4%) SSVs for GP12. After all filtering steps, the 11 somatic samples for each patient were found to contain 21548 and 21232 SSVs for GP5 and GP12, respectively. Annovar (version 2019Oct24) was used for the annotation of the SSVs using the following hg38 databases provided by the Annovar website: RefSeqGene 20190929, ExAC 65000 exome allele frequency data v0.3, dbNSFP v4.1c, gnomAD whole-genome data v3.0, and the COSMIC GRCh38 v92 database not included in the Annovar package.

Somatic tumor DNA pipeline variant calling validation

The quality of the somatic short variant calling using the "GP2Men pipeline" used for the current study was evaluated by direct comparison of WGS and deep sequencing data processed by the PCAWG consortium pipeline (9) and the GP2Men pipeline (Figs. S3 and S4). The validation dataset consisted of 10 WGS tumor-normal sample pairs that were sequenced using both Hiseq WGS and a targeted deep sequencing panel (TDSP) with a median depth of 471X (3) Any disagreeing calls between the PCAWG WGS and TDSP data were left out of the comparison as low-confidence calls. The PCAWG variant calls were used as ground truth for assessing the quality of the GP2Men pipeline (based on GATK/Mutect2 4.1.8.1). The GP2Men pipeline achieved precision and recall values of 0.992 and 0.970, respectively (Figs. S3 and S4). An integrated list of variants identified in GP5 and 12 is contained in Table S2.

Somatic tumor DNA structural variant and copy number analysis

Structural variants (SVs) were identified with SvABA v1.1.3 for tissue normal and tumor samples, using the corresponding patient blood DNA as normal (Tables S3 and S4). For the analysis, BQSR BAM files and known indels from the GATK Resource Bundle were used as input. Copy number alterations (CNAs) were analyzed by running Battenberg-hg38 v2.2.9 for matched tumor and blood normal samples, using BQSR BAMs as input (9). CNA burden was calculated for each sample

based on Battenberg copy number segments (Fig. S7). In addition to the default Battenberg hg38 reference data set, alleleCounter v. 4.0.2, Beagle 5.0 v12Jul19, and copynumber v1.26.0 were used. Breakpoints detected by SvABA were included in the Battenberg analysis for initial segmentation. Battenberg estimates of cancer sample purity (aberrant cell fraction) were adjusted slightly in 9 of 11 GP5 cancer samples and in 1 of 11 GP12 cancer samples for the evolutionary clustering analysis. This purity adjustment was done by setting a fixed copy number for a chromosome segment or segments where the copy number was known, and a new purity value was calculated from the logR and B-allele frequency (BAF) values of that segment.

Tumor chromosome X copy number analysis

The *AR*, located on Chr.Xq11-12 is often a focus of evolutionary pressure in prostate cancer. Battenberg can call copy number only in pseudo-autosomal regions (PARs) of the X chromosome (chrX) in males. This is because no heterozygous single nucleotide polymorphisms (SNPs) are present in the paired-normal sample outside of PARs due to haploidy of chrX in male patients. It is thus not possible to use segmentation of BAF of SNPs (which is either at 0 or 1) to infer CNAs for the entire length of chrX. We therefore developed a purely logR-based method to call CNAs of chrX in males, mainly to facilitate the identification of AR locus gain.

This step was implemented after autosomal CNAs were called by the standard tumor-normal mode in Battenberg. Because logR estimates, compared with BAF, tend to be noisy and, to some extent, fluctuate randomly not only across the genome but also from sample to sample, and to inform our logR-based analysis of such noise at the sample-level, we incorporated logR variation of autosomal CNA events in our chrX copy number estimation. This method first implements segmentation of SNP logR estimates across the chromosome using the piecewise constant fitting (PCF) method (10). The logR value of each segment is then corrected for non-zero deviation in regions where copy number is equal in normal and tumor samples. After testing for difference from normal copy state, the most likely total copy number is estimated by comparing the segment logR against purity-based expected logR values for copy loss and copy gains. Finally, a test of clonality informed by logR variation across the autosomal genome is undertaken, and if significant deviation is observed from the clonal state, the CCF and copy number states of the two major subclones are calculated. Finally, adjacent segments with the same CNA status were merged to avoid calling erroneous breakpoints.

Somatic tumor DNA variant clustering

Somatic single nucleotide variants (SNVs) detected in the 11 tumor samples from each patient underwent subclonal reconstruction using DPClust (11) based on the cancer cell fractions (CCFs) of each sample. The resulting clusters were further split into separate clusters if the CCF distribution in the cluster had multiple distinct binomial peaks remaining. The Dirichlet clustering process has a tendency to split clusters with a low CCF to two or more separate clusters (having 0.0 and ≤0.2 cluster median CCF). Such clusters were merged together. Clusters with less than 100 SNVs and a single cluster for each patient with a prominent but unknown trinucleotide signature, putatively signifying non-biological origin (Fig. S5), were excluded from the final clustering results. As a final step in the clustering process, remaining unassigned SNVs and indels were placed into the closest distance cluster using Euclidean distance. Chromosome X SNVs were assigned to existing clusters from DPClust afterwards by comparing the presence and absence profiles of SNVs in the samples followed by manual corrections for more complex cases. DPClust input preparation functions were used to calculate CCFs for indel counts obtained with

samtools v1.8 mpileup command and allelecount rev 4 and they were assigned to the closest clusters using Euclidean distance and penalizing samples where the mutation should not exist.

Cancer phylogenetic tree construction

After evolutionary clusters were identified, they were placed on an evolutionary tree based on non-conflicting median CCF values (Fig. 3a, Fig. 4a, Supplementary Text). Cluster median CCF values were estimated by fitting a left-truncated binomial distribution curve on the cluster SNV CCFs. This method was employed to establish an accurate median value for low CCF clusters (<0.05 CCF) where mutation call quality filtering may have removed low confidence evidence of SNV presence. The median CCF for clusters that had less than 40 non-zero SNV CCF values were set to zero CCF. For both patients, the evolutionary relationships between clusters in all 11 tumor samples were derived using the pigeonhole principle, along which the sum of direct descendant cluster median CCF values must be smaller than the median CCF of ancestral cluster in each sample. SSVs and CNAs matching recently described high risk prostate cancer drivers (12,13) (Table S5) were placed on the phylogenetic trees (Fig. 5b) by comparing the CCFs of the aberration to evolutionary cluster CCFs in every sample. A more detailed view of the annotations is provided in Figs. S8 and S9. “Jawbreaker” plots (Fig. 3c, Fig. 4c, Fig. S6) were generated to show the evolutionary assignment of clonal and subclonal cancer cell populations present in each sample at the time of radical prostatectomy.

Genome-wide DNA somatic copy number, structural variant, and subclone plots

Circos v0.69-9 (14) was used to visualize Battenberg CNAs and separately detected chromosome X CNAs across all samples (Fig. 5a). Total copy number for subclonal segments was calculated as a fractional sum of the two subclone copy numbers. Summed length of Battenberg CNA and chromosome X CNA segments with unambiguously altered copy number below 1.5 or above 2.5 was divided by the total length of autosomes and chromosome X to get the CNA burden estimates in each sample (Fig. S7). Statistical significance between samples containing large (>0.8) metastatic subclone fraction and the remaining samples was estimated using the Wilcoxon rank sum test. For visualizing the genome-wide CNAs and SVs in each chromosome (Figs. S8 and S9), a custom script was developed.

SUPPLEMENTARY RESULTS

Sample quality

DNA quality (average GQN 5.1 for both cases) and sequencing performance was good. Macro tissue cassettes stacked in a processing basket shifted and jammed the processing of GP5 tissues for 12h while in 90% ethanol. Volumes of Interest (VOI) undergoing DNA analysis were selected to obtain a broad anatomic distribution including surgical margins and all discrete metastatic sites (Figs. 1 and 2).

Cancer phylogenetic tree construction

The tumors in both patients were discovered to have a monoclonal origin. The large majority of known oncogenic aberrations were present in the most recent common ancestor cluster (MRCA), and therefore, in all detected cancer cell populations. The reconstruction of the evolutionary trees show a distinct pattern of branching evolution(15). The clustering of SNVs reveals one to seven prominently divergent cancer cell populations being present in each sampled region of the tumor (Figs. 3 and 4). The spatial intermixing of multiple subclonal populations is not a universal phenomenon in all cancer types but has been reported in PrCa previously(3) and corroborated by

our data. Each cluster (node) of the evolutionary tree represents a clonal expansion of a single cell that became the ancestor of a multitude of cells to reach a reliably detectable CCF of ~0.03.

With the exception of one cluster, the lineage and ancestor-daughter relationships of all the clusters could be confidently and unambiguously deduced. Patient GP5 evolutionary cluster Bc had multiple, non-conflicting placement options in the phylogenetic tree. The most-likely placement as daughter of cluster B was chosen based on the similarities of median CCFs between the clusters in the three samples where both clusters are present. For GP5 and GP12, 1229/19590 (6.2%) and 1181/18503 (6.4%) SNVs could not be confidently assigned to any evolutionary cluster.

Examining the trinucleotide contexts of the SNVs revealed both patients to have a cluster that is present in all tissue samples with a distinct mutational signature (Fig. S5). While the signature has a similarity to SBS48(16) ("possible sequencing artifact"), its origin and significance are currently unknown. The trinucleotide contexts of the mutations assigned to these signature clusters in both patients are almost exclusively TCG>TAG, TCA>TAA, CCA>CTA and ACA>ATA. It is possible that two different sources of artifacts are combined and assigned to this cluster. Because they are likely of non-biological origin, the 815 variants in GP5 and 338 in GP12 belonging to this signature cluster were removed from the evolutionary analysis. Further testing is needed to determine the origin of this potentially novel signature not included in the COSMIC SBS database(16). Association of this possibly novel signature with PAXgene fixation cannot be ruled out.

GP5 cancer evolution

Reconstruction of the phylogenetic tree of GP5 cancer the primary tumor and metastases shows 14 clearly distinguishable branching events during the evolution of the cancer (Fig. 5b). The largest fractions of the MRCA cancer cell populations were found in samples 6-LCA1 and 7-LCA2, indicating the left posterior region of the apex as the origin of the cancer. From its point of origin, the carcinoma split in to two evolutionary clusters denoted by clusters A and B. Cluster B cells are present most prominently in the left side of the apex (sample 2-LApexCA), while reaching into the rightmost sampled region as well (1-RApexCA). Cluster A cells occupy the right side of the apical region of the prostate and reach more prominently into the sampled mid-section of the gland (samples 3-8). Markedly, while Cluster A cells represent the larger tumor mass in the sampled regions, only Cluster B cells and its descendants are found in the LN metastases. While the A-branch has 26% less SNVs in comparison to the B-branch (3221 vs. 4361), analysis of the putatively oncogenic driver events shows an even larger imbalance towards the metastatic B-branch. 17 putatively oncogenic driver events were identified in the B-branch, while only 3 events were identified in the A-branch.

One notable difference between the A and B Cluster cell populations is a tandem duplication in the AR gene in chromosome X. The best fit for the duplicated region with the evolutionary data indicates that the duplication of the AR and AR-enhancer regions has happened twice independently during the evolution, in clusters Bb and Baa (Fig. S8). The androgen receptor and pathways affecting it are the most prominent oncogenic drivers in advanced prostate cancer (12). Cluster Bb cells and their descendants are found with low CCFs (≤ 12%) in the primary tumor samples, but nearly clonal (≥ 86%) in all LN metastasis, highlighting their increased metastatic potential.

The LN metastases were found to contain cancer cells representing 5 different evolutionary clusters (Bb2, Bb1, Bb, Ba, Baa) represented by the surface layers of the jawbreaker plots (Fig.

3c). This suggests that there have been multiple extraprostatic spreading events during the evolution of the cancer, or even continuous migration of the cancer cells. The cancer cell population found on the right-side metastasis (9-RPelvicLNMet1) consist of 98% cluster Bb2 cells, which are present only in the primary tumor sample RApexCA, indicating the right apical region as the putative source of the spread. On the left side LN metastasis, the cancer cell populations are markedly different in comparison to the right side, consisting of cluster Bb1 (~75%), Bb (~15%), Ba (~7.5%), Baa (2.5%) cells. The dominant population of Bb1 cells is only detected in the primary tumor in sample 6-LCA1 (CCF 3%) implicating it as the region of left side metastatic spread. Overall, the origin of the extraprostatic spread of the cancer cells can be traced back to multiple regions across the apex of the prostate (Fig. 6).

The left side metastatic LN region 10-LPelvicMet1 is larger (~20mm width) than the right side metastasis (~2mm width). In addition, left pelvic lymph nodes show a prominent peritumoral fibrosis and lymph node effacement absent in the positive right pelvic lymph node (Fig. 1) and the prevalent clone, Bb1, present in sampled region 10-RPelvicLNMet suggest that it is the most advanced lineage of the cancer with the highest metastatic potential. Cluster Bb1 is also one of the three clusters, along with clusters Baa and Bb2, that have undergone the most clonal expansions in the metastatic B-branch.

GP12 cancer evolution

Reconstruction of the phylogenetic tree of the GP12 primary tumor and metastases showed 14 clearly distinguishable branching events during the evolution of the cancer (Fig. 5b). The most ancestral form of the cancer was found in the left side of the tumor, near the apex (sample 2-LMidApical), indicating this region as the origin of the cancer. By combining the evolutionary information with the anatomy of the tumor we were able to reconstruct the path of tumor growth (Fig. 6). From its region of origin, the tumor grew along the posterior side, towards the apex, base and the right side of the gland while accumulating unique genetic aberrations in each direction of growth (Clusters A-D). Interestingly, as the tumor grew over the midline of the gland (left to right) it separated into two distinct populations (clusters Cba and Cbb) and this divide is prominently present in the lymph node metastases as well. The cancer cell populations in sampled region 7-RSVBaseCA closely resemble the cancer cell populations found in the right-side lymph nodes, implicating it as the origin of the right-side LN metastases. Furthermore, sample 7-RSVBaseCA is also the only known primary tumor location for cluster Cba2, which represents the dominant clone (CCF ~90%) in the right-side LN metastases. The closest resemblance of the cancer cell populations found in the left side lymph node metastasis is present in sample 8-LSVBaseCA, strongly implicating it as the region of left side metastatic spread. Sample 8-LSVBaseCA is also the only known location of cluster Cbb2 which is found in the left side lymph node as a subclonal population (CCF <5%).

The lymph node metastases have a total of 8 different cancer cell populations (clusters B,Ca,Cb,Cba1,Cba2,Cbb,Cbb1,Cbb2) present in the three sampled regions indicating multiple spreading events or even continuous spread of the cancer cells, similarly to patient GP5. The basal region of the prostate gland contains the most advanced forms of the cancer and is strongly indicated as the main source of metastatic spread with clear divide between the left and right sides. The only exception to the basal region as the source of extraprostatic spread is represented by cluster B. Cluster B is present in a low CCF of 4% in the left side LN metastases and is only found in the left apex region of the prostate (samples 6-LApexCA and 2-LMidApicalCA).

Value of multiregional sampling
In the case of patient GP5, the primary tumor is made up of two populations that have significantly different genotypes, and the dominant lesion (in terms of size of tumor) represents the less aggressive, non-metastatic form of the cancer (green A-branch). The metastatic and non-metastatic lesions are inseparable based on their histology alone. If the non-metastatic lesion was selected as the single sampled region, 40% (14/35) of the putatively oncogenic cancer driving genetic aberrations would remain completely undetected (Fig. 5b). Furthermore, the independently occurred duplications of the AR and AR-enhancer regions present in the metastatic clusters Baa & Bb would also remain undetected, leaving out crucial information about metastatic potential and possible response to ADT (androgen deprivation therapy).

On the other hand, as new treatment options become available, selecting a treatment that is effective on the largest possible number of cancer cells requires identifying somatic aberrations present in the MRCA. With the single sampling method, identifying somatic aberrations belonging to the MRCA is based on their VAF (or CCF when taking sample aberrant cell fraction into account), so that the mutations with the highest VAF are the most probable of belonging to the MRCA. Our data shows that in 9 out of the 11 sampled GP5 regions mutations belonging to either the A or B cluster, would be indistinguishable from the MRCA due to their >=90% CCF in each of the samples. Selecting any of these A or B cluster mutations as the basis of the treatment, would exclude roughly half of the cancerous cells. In the case of selecting a Cluster A mutation as the basis of the treatment, 100% of the metastatic cells would be unaffected.

The tumor in patient GP12 reaches from the apex to the base of the prostate. Selecting a region for a single sample experiment would most likely include either sample 1-RMidApical or 2-LMidApical as the largest tumor mass is present there. Considering having only one of these regions as the representation of the cancer in the patient would yield significant differences in the results. In 2-LMidApical, a genetic analysis would be able to identify cluster T as the MRCA, but show a grave underrepresentation of clusters Ta (30%) and the metastatic C-branch (5%), while in reality these clusters represent 93% of primary tumor population fractions and 98.6% of the metastatic populations. Using 1-RMidApical for a single sample experiment would find the non-metastatic GP12 Cluster A at a nearly clonal fraction of 0.83, giving way to similar problems as with the selection of GP5 cluster A mutations for the basis of treatment.

Druggability analysis
The majority of the potentially druggable genes were ones where a gain was observed (11 / 15 druggable changes in GP5 and 6 / 9 druggable changes in GP12). The *BRCA2* frameshift + loss of heterozygosity in GP12 is a druggable candidate, although *BRCA2* reversion mutations as observed previously in circulating tumor DNA might have implications for development of resistance to PARP inhibitors (17).

SUPPLEMENTARY REFERENCES

1. McKenna A, Hanna M, Banks E, Sivachenko A, Cibulskis K, Kernytsky A, et al. The Genome Analysis Toolkit: A MapReduce framework for analyzing next-generation DNA sequencing data. Genome Res. 2010 Sep 1;20(9):1297–303.
2. Wala JA, Bandopadhayay P, Greenwald NF, O'Rourke R, Sharpe T, Stewart C, et al. SvABA: genome-wide detection of structural variants and indels by local assembly. Genome Res. 2018;28(4):581–91.
3. Woodcock DJ, Riabchenko E, Taavitsainen S, Kankainen M, Gundem G, Brewer DS, et al. Prostate cancer evolution from multilineage primary to single lineage metastases with implications for liquid biopsy. Nature Communications. 2020 Oct 8;11(1):5070.
4. Gundem G, Van Loo P, Kremeyer B, Alexandrov LB, Tubio JMC, Papaemmanuil E, et al. The evolutionary history of lethal metastatic prostate cancer. Nature. 2015 Apr 16;520(7547):353–7.
5. Gerstung M, Jolly C, Leshchiner I, Dentro SC, Gonzalez S, Rosebrock D, et al. The evolutionary history of 2,658 cancers. Nature. 2020;578(7793):122–8.
6. Amemiya HM, Kundaje A, Boyle AP. The ENCODE Blacklist: Identification of Problematic Regions of the Genome. Sci Rep [Internet]. 2019 Jun 27 [cited 2020 Feb 25];9. Available from: https://www.ncbi.nlm.nih.gov/pmc/articles/PMC6597582/
7. Högnäs G, Kivinummi K, Kallio HML, Hieta R, Ruusuvuori P, Koskenalho A, et al. Feasibility of Prostate PAXgene Fixation for Molecular Research and Diagnostic Surgical Pathology: Comparison of Matched Fresh Frozen, FFPE, and PFPE Tissues. Am J Surg Pathol. 2018 Jan;42(1):103–15.
8. Zhang J, Baran J, Cros A, Guberman JM, Haider S, Hsu J, et al. International Cancer Genome Consortium Data Portal--a one-stop shop for cancer genomics data. Database (Oxford). 2011;2011:bar026.
9. Nik-Zainal S, Van Loo P, Wedge DC, Alexandrov LB, Greenman CD, Lau KW, et al. The life history of 21 breast cancers. Cell. 2012 May 25;149(5):994–1007.
10. Nilsen G, Liestøl K, Van Loo P, Moen Vollan HK, Eide MB, Rueda OM, et al. Copynumber: Efficient algorithms for single- and multi-track copy number segmentation. BMC Genomics. 2012 Nov 4;13(1):591.
11. Bolli N, Avet-Loiseau H, Wedge DC, Van Loo P, Alexandrov LB, Martincorena I, et al. Heterogeneity of genomic evolution and mutational profiles in multiple myeloma. Nature Communications. 2014 Jan 16;5:2997.
12. Robinson D, Van Allen EM, Wu YM, Schultz N, Lonigro RJ, Mosquera JM, et al. Integrative clinical genomics of advanced prostate cancer. Cell. 2015 May 21;161(5):1215–28.
13. Grasso CS, Wu YM, Robinson DR, Cao X, Dhanasekaran SM, Khan AP, et al. The mutational landscape of lethal castration-resistant prostate cancer. Nature. 2012 Jul 12;487(7406):239–43.
14. Krzywinski MI, Schein JE, Birol I, Connors J, Gascoyne R, Horsman D, et al. Circos: An information aesthetic for comparative genomics. Genome Res [Internet]. 2009 Jun 18 [cited 2021 Mar 30]; Available from: https://genome.cshlp.org/content/early/2009/06/15/gr.092759.109
15. Davis A, Gao R, Navin N. Tumor evolution: Linear, branching, neutral or punctuated? Biochimica et Biophysica Acta (BBA) - Reviews on Cancer. 2017 Apr 1;1867(2):151–61.
16. Alexandrov LB, Kim J, Haradhvala NJ, Huang MN, Tian Ng AW, Wu Y, et al. The repertoire of mutational signatures in human cancer. Nature. 2020 Feb;578(7793):94–101.
17. Lin KK, Harrell MI, Oza AM, Oaknin A, Ray-Coquard I, Tinker AV, et al. BRCA Reversion Mutations in Circulating Tumor DNA Predict Primary and Acquired Resistance to the PARP Inhibitor Rucaparib in High-Grade Ovarian Carcinoma. Cancer Discov. 2019 Feb 1;9(2):210–9.

List of Abbreviations in Supplementary Information (alphabetical)

AR: androgen receptor
BAF: B-allele frequency
BQSR: Base Quality Score Recalibration
CCF: cancer cell fraction
ChrX: X chromosome
CNA: copy number alteration
GQN: Genomic DNA Quality Number
GT: ground truth
HE: hematoxylin and eosin
ICGC: International Cancer Genome Consortium
LN: lymph node
MR: magnetic resonance
MRCA: most recent common ancestor
PAR: pseudo-autosomal region
PCAWG: Pan-cancer analysis of whole genomes (project)
PCF: piecewise constant fitting
PET: positron emission tomography
PoN: panel of normals
PrCa: prostate cancer
ROI: region of interest
RP: radical prostatectomy
SBS: single base substitution
SNV: single nucleotide variant
SSV: somatic short variant
SV: structural variant
SV: seminal vesicle
TD: tandem duplication
TDSP: targeted deep sequencing panel
VOI: volume of interest
VST: variance stabilizing transformation
WGS: whole genome sequencing
WSI: whole-slide imaging
